# Mechanisms of motor symptom improvement by long-term Tai Chi training in Parkinson’s disease patients

**DOI:** 10.1186/s40035-022-00280-7

**Published:** 2022-02-07

**Authors:** Gen Li, Pei Huang, Shi-Shuang Cui, Yu-Yan Tan, Ya-Chao He, Xin Shen, Qin-Ying Jiang, Ping Huang, Gui-Ying He, Bin-Yin Li, Yu-Xin Li, Jin Xu, Zheng Wang, Sheng-Di Chen

**Affiliations:** 1 Department of Neurology and Institute of Neurology, Ruijin Hospital Affiliated to Shanghai Jiao Tong University School of Medicine, Shanghai, 200025 China; 2 Institute of Neuroscience, State Key Laboratory of Neuroscience, Key Laboratory of Primate Neurobiology, CAS Center for Excellence in Brain Science and Intelligence Technology, Shanghai Institute for Biological Sciences, Chinese Academy of Sciences, Shanghai, 200031 China; 3 Shanghai Key Laboratory for Bone and Joint Diseases, Shanghai Institute of Orthopedics and Traumatology, Ruijin Hospital Affiliated to Shanghai Jiao Tong University School of Medicine, Shanghai, 200003 China; 4 School of Psychological and Cognitive Sciences, Beijing Key Laboratory of Behavior and Mental Health, IDG/McGovern Institute for Brain Research, Peking-Tsinghua Center for Life Sciences, Peking University, Beijing, 100871 China

**Keywords:** Parkinson’s disease, Tai Chi, Motor symptoms, Mechanism, Brain network

## Abstract

**Background:**

Tai Chi has been shown to improve motor symptoms in Parkinson’s disease (PD), but its long-term effects and the related mechanisms remain to be elucidated. In this study, we investigated the effects of long-term Tai Chi training on motor symptoms in PD and the underlying mechanisms.

**Methods:**

Ninety-five early-stage PD patients were enrolled and randomly divided into Tai Chi (*n* = 32), brisk walking (*n* = 31) and no-exercise (*n* = 32) groups. At baseline, 6 months and 12 months during one-year intervention, all participants underwent motor symptom evaluation by Berg balance scale (BBS), Unified PD rating-scale (UPDRS), Timed Up and Go test (TUG) and 3D gait analysis, functional magnetic resonance imaging (fMRI), plasma cytokine and metabolomics analysis, and blood Huntingtin interaction protein 2 (*HIP2*) mRNA level analysis. Longitudinal self-changes were calculated using repeated measures ANOVA. GEE (generalized estimating equations) was used to assess factors associated with the longitudinal data of rating scales. Switch rates were used for fMRI analysis. False discovery rate correction was used for multiple correction.

**Results:**

Participants in the Tai Chi group had better performance in BBS, UPDRS, TUG and step width. Besides, Tai Chi was advantageous over brisk walking in improving BBS and step width. The improved BBS was correlated with enhanced visual network function and downregulation of interleukin-1β. The improvements in UPDRS were associated with enhanced default mode network function, decreased *L*-malic acid and 3-phosphoglyceric acid, and increased adenosine and *HIP2* mRNA levels. In addition, arginine biosynthesis, urea cycle, tricarboxylic acid cycle and beta oxidation of very-long-chain fatty acids were also improved by Tai Chi training.

**Conclusions:**

Long-term Tai Chi training improves motor function, especially gait and balance, in PD. The underlying mechanisms may include enhanced brain network function, reduced inflammation, improved amino acid metabolism, energy metabolism and neurotransmitter metabolism, and decreased vulnerability to dopaminergic degeneration.

*Trial registration* This study has been registered at Chinese Clinical Trial Registry (Registration number: ChiCTR2000036036; Registration date: August 22, 2020).

**Supplementary Information:**

The online version contains supplementary material available at 10.1186/s40035-022-00280-7.

## Background

Parkinson’s disease (PD) is the second most common neurodegenerative disease globally, characterized by bradykinesia, resting tremor and rigidity [1]. With progression of the disease, patients lose postural stability and have difficulty in gait and balance, causing frequent falls and disability in daily living [2]. Although some motor symptoms, such as tremor and rigidity, can be alleviated by drug therapy, some clinical features such as postural instability are less responsive to medication and need alternative treatments [3].

Physical exercise has been shown to improve mobility, gait, balance and quality of life in PD [4, 5]. Tai Chi, brisk walking and tango dancing have demonstrated the highest level of evidence of efficacy, especially in improving postural stability [6–8]. Tai Chi, a mind–body exercise that utilizes continuous, curved, and spiral body movements with breathing control [9], can improve aerobic capacity, muscle strength, balance, and motor control, as well as reducing stress and anxiety in older adults [10]. Evidence from randomized controlled trials by Fuzhong Li et al. shows improvement of maximal excursion, direction control, gait velocity and quality of life after 6-month Tai Chi training in PD patients [6, 11]. However, previous studies focusing on Tai Chi training only showed short-term (up to 6 months) benefits for PD patients. Owing to the progressive nature of PD, the long-term effects of such interventions should be concerned.

More importantly, the beneficial mechanisms of Tai Chi remain unclear. Evidence based on animal studies of neurodegenerative diseases shows that physical exercise can improve the production of neurotrophic factors, neurotransmitters, and hormones, promoting processes such as synaptic plasticity, neurogenesis, angiogenesis, and autophagy [12]. Several studies focusing on the mechanisms of Tai Chi in older adults have shown improved brain metabolism and muscle energetics using brain ^1^H magnetic resonance spectroscopy (MRS) and muscle ^31^P MRS [13], as well as enhanced default mode network (DMN) connectivity using resting-state functional magnetic resonance imaging (fMRI) [14]. However, the mechanisms of Tai Chi training in PD patients have not been investigated.

fMRI and blood biomarker tests will probably give us a deep insight into the mechanisms of Tai Chi in PD. Resting-state fMRI is widely used to explore brain function and neuroplasticity at the macro level. In addition, molecular biomarkers in the blood of PD patients, which can reflect pathogenesis and disease progression, also provide ways to study mechanisms of Tai Chi [15, 16]. Previous animal studies showed that exercise might benefit PD patients through inhibiting oxidative stress, repairing mitochondrial damage, and promoting the production of growth factors [4]. Huntingtin interaction protein 2 (HIP2) is an E2 ubiquitin-conjugating enzyme associated with neurodegenerative diseases. Decreased expression of *HIP2* has been reported in the blood [17–19] and the substantia nigra of PD patients [20]. Reduction of *HIP2* expression could cause motor function impairment and increase vulnerability to dopaminergic degeneration in PD models [21].

In this study, we conducted a one-year randomized controlled trial to investigate the long-term effect of Tai Chi training on motor symptoms of PD and explore the underlying mechanisms by fMRI and blood biomarker (including cytokines, metabolomics and *HIP2* mRNA) analysis.

## Methods

### Participants

Ninety-five early-stage (Hoehn-Yahr 1–2.5) PD patients (50–80 years old) were recruited. The medication was stable for at least 3 months before recruitment and not changed during follow-up unless necessary due to disease progression. This study was approved by Ruijin Hospital Ethic Committee of Shanghai Jiao Tong University School of Medicine on December 4, 2014. This study has been registered at Chinese Clinical Trial Registry (Registration number: ChiCTR2000036036; Registration date: August 22, 2020). PD was diagnosed by two movement disorder specialists (SDC, YYT). All of the PD patients met both the PD diagnostic criteria of United Kingdom Brain Bank [22] and Movement Disorders Society [23]. All participants provided written informed consents.

### Randomization

After recruitment, participants were randomly assigned to Tai Chi (*n* = 32), brisk walking (*n* = 31) and control (*n* = 32) groups without stratification. The randomization method is to draw lots. Then, 12-month exercise intervention (Tai Chi, brisk walking or non-exercise) with strict quality control was introduced. Details could be seen in Additional file 1.

### Assessments

Berg Balance Scale (BBS), Unified PD rating scale (UPDRS), Time Up and Go test (TUG) and spatial 3D gait analysis were used to assess motor symptoms, gait and balance of PD patients. Assessments were performed at baseline, 6 months and 12 months during the intervention on medication.

### Investigation of mechanisms

fMRI, plasma cytokines and metabolomics, and the blood *HIP2* mRNA level were assessed at baseline, 6 months and 12 months. Details are provided in Additional file 1.

### Statistical analysis

Statistical analysis was performed using R (version 3.5.1), RStudio (version 1.1463) and related packages. fMRI data were analyzed using the MATLAB R2018a (version 9.4.0.813654, MathWorks, Inc.) software.

All analyses about clinical measurements were conducted on an intention-to-treat basis. Analysis of variance (ANOVA) was used to compare numerical demographic information. Pearson Chi-square test or Fisher test was used for analysis of categorical demographic information. Shapiro–Wilk normality test was used to assess the normality of variables. Independent-sample *t*-tests (with 95% confidence intervals) were used to compare group means. Data were presented in the way of between-group differences. The longitudinal self-changes were analyzed with repeated measures ANOVA. AI-Therapy Statistics^BETA^ (https://www.ai-therapy.com/psychology-statistics/power-calculator) was used to calculate the statistical power.

To analyze longitudinal fMRI data, GroupICAT (v4.0a, The MIND Research Network, Atlanta, GA) was used for group-level independent component analysis (ICA) to extract brain networks. Longitudinal changes of fMRI data were reflected by switching rate according to the protocol given by Pedersen et al. [24], in which 25 brain nodes were analyzed and sliding-window analysis was used to see brain network dynamic changes. Thus, functional network dynamic connectivity was analyzed to obtain the switching rate of brain networks. Statistical methods such as LASSO (least absolute shrinkage and selection operator) were used for screening and feature selection of high latitude variables (switching rates of various brain networks). To make the data normally distributed, Log transformation was introduced to selected switching rates. Finally, linear regression (y = ∑x) was performed between the change of rating scales (regarded as y) and the selected switching rates (regarded as x). Bayesian belief network was also introduced to confirm the causality of the associations. Finally, false discovery rate (FDR) correction was adopted.

GEE (generalized estimating equations) was used to assess the association of longitudinal data of rating scales. Mixed effect regression using restricted maximum likelihood method was introduced to analyze cytokine levels, *HIP2* levels and metabolites. Bonferroni correction was used for multiple corrections. Pathway analysis was performed using MetaboAnalyst 4.0 platform. Enrichment analysis was done using MetaboAnalyst 4.0 platform referenced with the small molecule pathway database (SMPDB). FDR correction was used for multiple correction. Detailed information is provided in Additional file 1.

## Results

### Baseline characteristics of participants

**Fig. 1:**
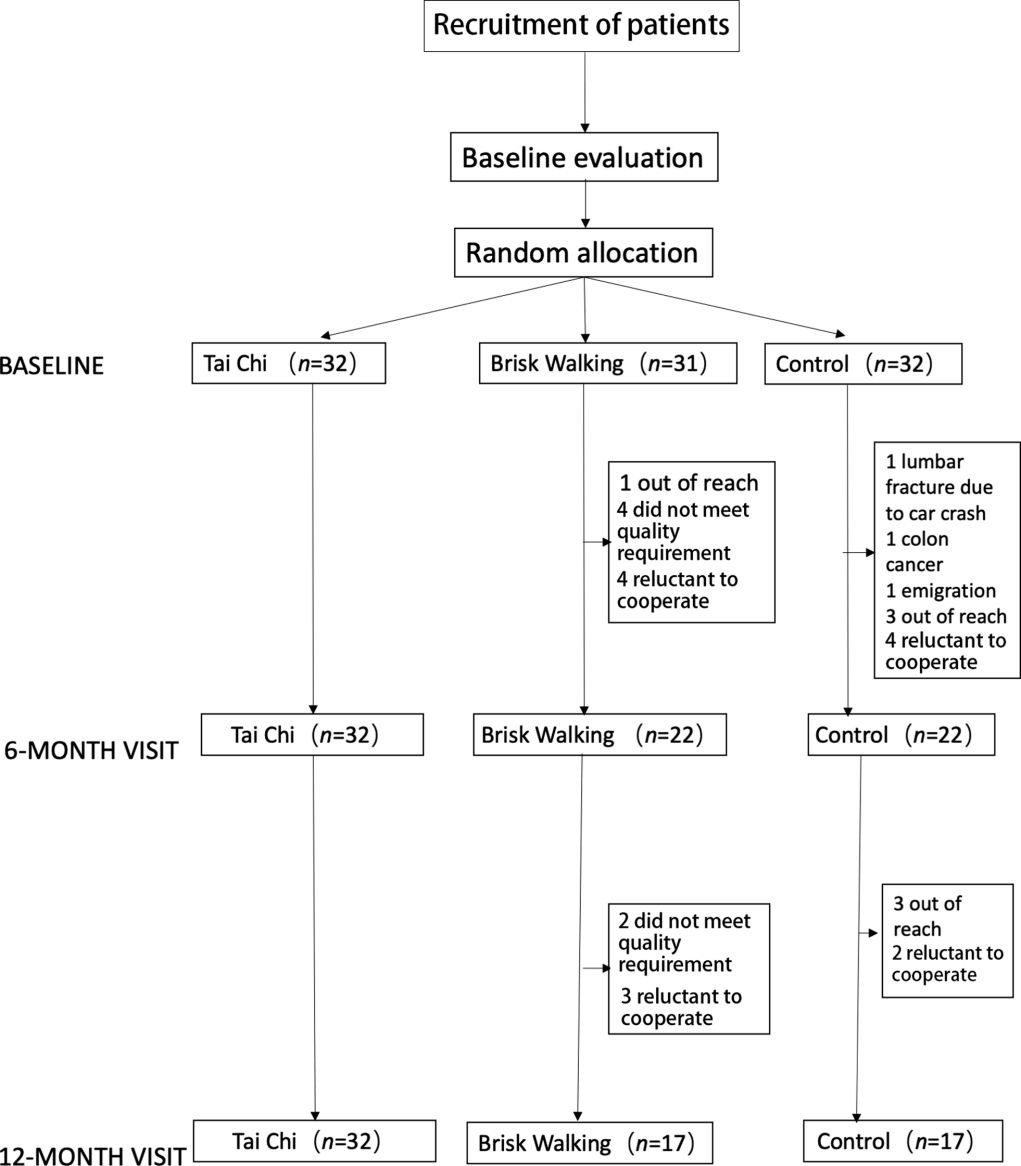
Flow chart of patient recruitment and follow-ups

Ninety-five PD patients were recruited in this study, including 32 in the Tai Chi group, 31 in the brisk walking group, and 32 in the control group (see Fig. 1 for a flowchart of recruitment). All of them are of Han ethnicity, and none presented either on–off phenomenon or dyskinesias. The three groups were matched in age, sex, disease duration and education. Sixty-six patients finished 6-month and 12-month follow-ups. Detailed demographic information is shown in Table 1. There were no differences in the levodopa equivalent daily dosage (LEDD) at baseline and at 12-month visit among the groups.

**Table 1:** Demographic information of participants

	Tai Chi Group (*n* = 32)	Brisk Walking Group (*n* = 31)	Control Group (*n* = 32)	*P* value
Sex, female, *n* (%)	15 (46.88)	9 (29.41)	13 (41.18)	0.500
Age at baseline (mean ± SD)	62.7 (5.51)	61.9 (5.64)	61.9 (6.76)	0.400
Education, years (mean ± SD)	13.60 (2.71)	13.10 (2.57)	12.40 (2.83)	0.472
History of hypertension, *n* (%)	7 (21.88)	3 (11.76)	5 (17.65)	0.800
History of diabetes mellitus, *n* (%)	1 (3.13)	0 (0.00)	1 (3.13)	1.000
History of smoking, *n* (%)	2 (6.25)	2 (6.25)	2 (6.25)	0.600
Family history, *n* (%)	8 (25.00)	5 (17.65)	3 (11.76)	0.600
Tremor dominant, *n* (%)	22 (68.75)	22 (64.71)	17 (58.82)	0.800
Disease duration (mean ± SD)	5.91 (4.01)	3.82 (1.87)	4.32 (2.46)	0.082
Hoehn-Yahr staging, *n* (%)				0.097
1.0	9 (28.13)	8 (25.81)	1 (3.13)	
1.5	5 (15.63)	7 (22.58)	11 (34.38)	
2.0	13 (40.63)	13 (41.94)	13 (40.63)	
2.5	5 (15.63)	3 (9.68)	7 (21.88)	
LEDD at baseline (mean ± SD)	326 (197)	260 (174)	347 (109)	0.800
ΔLEDD (mean ± SD)	56.33 (91.68)	39.71 (83.30)	57.21 (107.24)	0.939

*LEDD* levodopa equivalent daily dosage; *N* number; *SD* standard deviation

*ΔLEDD* LEDD at 12 months minus LEDD at baseline

### Clinical improvements

**Fig. 2:**
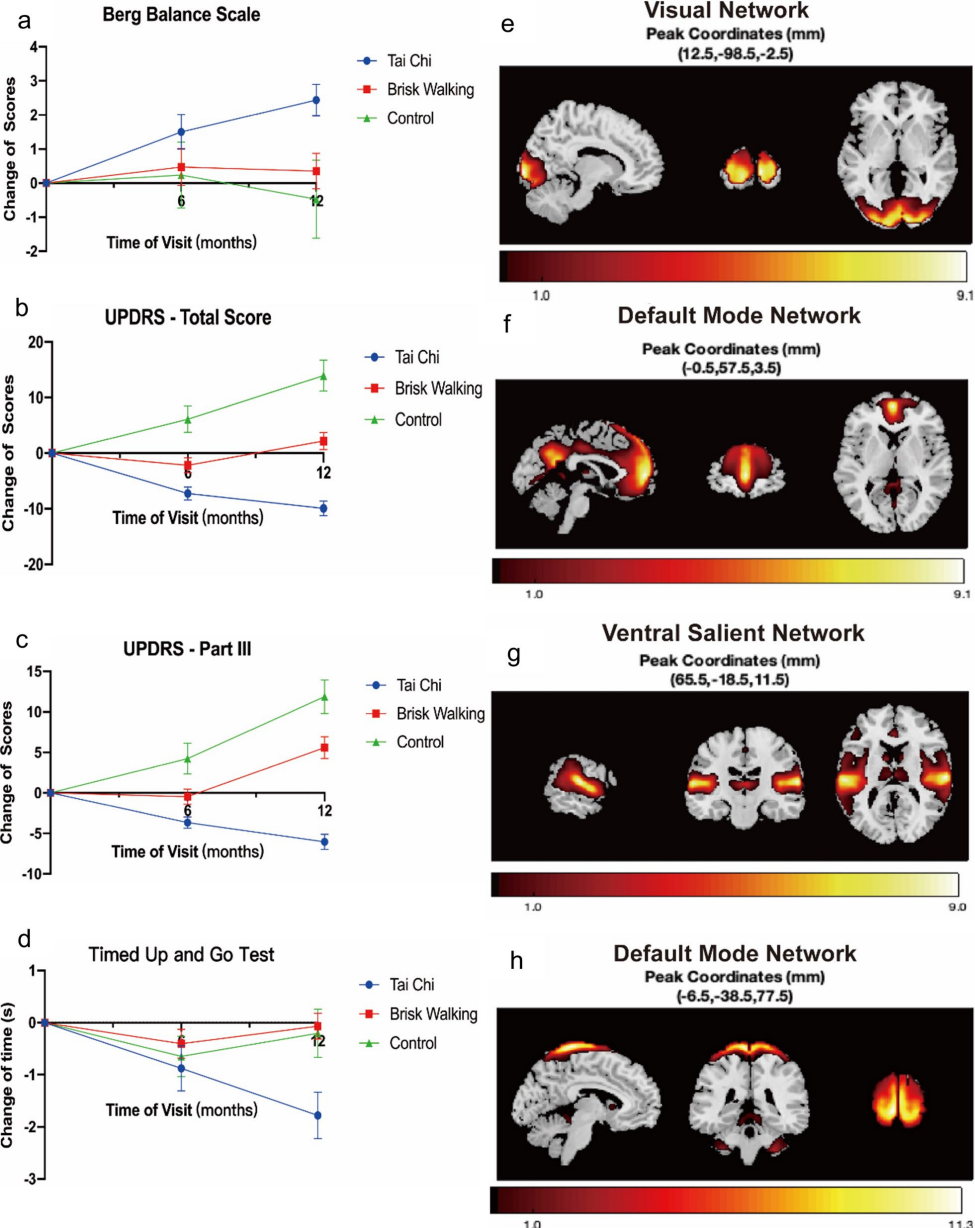
Changes of motor symptoms between groups and related fMRI changes. **a**–**d** Changes of rating scales in **a** Berg Balance Scale, **b** UPDRS total Score, **c** UPDRS Part III, and **d** Timed Up and Go Test in the 3 groups. Data are shown as mean ± SEM. **e–h** Neural networks associated with the longitudinal changes of rating scales from 12-month to baseline after Tai Chi training. Bright yellow indicates more positive association between neural networks and changes of rating scales. **e** Visual network associated with Berg Balance scale; **f** default mode network associated with UPDRS total score; **g** Ventral salient network associated with UPDRS total score, but the association was not significant; **h** default mode network associated with UPDRS Part III

The PD patients in the Tai Chi group showed significant improvements in BBS at 6 months and 12 months (6 months: *P* = 0.006, 12 months: *P* = 0.044, *P* values were based on between-group differences in longitudinal mean changes of score), compared to the control group. Compared to brisk walking, Tai Chi had more advantages in improving BBS scores (at 6 months: *P* = 0.005, 12 months: *P* = 0.022) (Additional File 2: Table S1; Fig. 2a).

Regarding UPDRS and TUG, the Tai Chi group showed significantly greater improvement in UPDRS at 12 months compared to controls (UPDRS total score: *P* = 0.015, UPDRS-III: *P* < 0.001), as well as significantly greater improvement in TUG at 6 and 12 months compared to control (6 months: *P* = 0.017, 12 months: *P* = 0.011). Tai Chi also significantly improved patients’ step width compared to controls at 6 and 12 months (severer side, 6 months: *P* = 0.002, 12 months: *P* < 0.001; milder side, 6 months: *P* < 0.001, 12 months: *P* < 0.001). When compared to brisk walking, the Tai Chi group also showed greater improvement in step width (severer side, 6 months: *P* = 0.03, 12 months: *P* = 0.03; milder side, 6 months: *P* = 0.004, 12 months: *P* = 0.111). Statistical powers were above 0.05 (BBS: 0.052; UPDRS total score: 0.059; UPDRS-III: 0.050; TUG: 0.061). (Additional file 2: Table S1, Fig. 2b–d).

### fMRI switch rates

The better performance in BBS in the Tai Chi group compared with the control was associated with the change of visual network (VN) (*P* = 0.044). The improvements in UPDRS total score (*P* = 0.023) and UPDRS-III (*P* = 0.006) of PD patients were associated with enhancement of DMN connectivity after Tai Chi training (Additional File 2: Table S2; Fig. 2e–h).

### Cytokines

The levels of interleukin (IL)-1β (6 months: *P* = 0.013, 12 months: *P* = 0.028), IL-5 (6 months: *P* = 0.028, 12 months: *P* = 0.248), IL-7 (6 months: *P* = 0.032, 12 months: *P* = 0.016) and IL-9 (6 months: *P* = 0.037, 12 months: *P* = 0.019) were significantly downregulated in the Tai Chi group compared with the control group (Additional file 2: Table S3).

The levels of IL-13 (6 months: *P* = 0.002, 12 months: *P* = 0.005 *vs* control), platelet-derived growth factor-BB (6 months: *P* = 0.022, 12 months: *P* = 0.011 *vs* control), macrophage inflammatory protein-1β (MIP-1β) (6 months: *P* = 0.003, 12 months: *P* = 0.008 *vs* control) and monocyte chemoattractant protein-1 (MCP-1) (6 months: *P* = 0.032, 12 months: *P* = 0.06 *vs* control) were stable in the Tai Chi group, but significantly increased in controls. Relatively stable levels of IL-13 (6 months: *P* = 0.019, 12 months: *P* = 0.021 *vs* brisk walking group) and MCP-1 (6 months: *P* = 0.0007, 12 months: *P* = 0.022 *vs* brisk walking group) were seen in the Tai Chi group compared with the brisk walking group (Additional file 2: Table S3). In addition, at 6 months, upregulation and downregulation of MIP-1α were seen in the control group and brisk walking group, respectively, while the MIP-1α level did not change in the Tai Chi group (*P* = 0.006 control *vs* Tai Chi, *P* = 0.029 brisk walking group *vs* Tai Chi). But MIP-1α returned to the baseline level in the three groups at 12 months (Additional file 2: Table S3).

Granulocyte–macrophage colony stimulating factor (GM-CSF) was upregulated in the Tai Chi group while being not changed in the control group or the brisk walking group (Tai Chi *vs* control, 6 months: *P* = 0.005, 12 months: *P* = 0.011; Tai Chi *vs* brisk walking, 6 months: *P* = 0.006, 12 months: *P* = 0.014) (Additional file 2: Table S3).

Furthermore, by analyzing the association between changes of cytokines and changes of clinical presentation, we found that the downregulation of IL-1β was positively related to the improved BBS score (*P* ≤ 0.031) (Additional file 2: Table S4).

### Metabolomics

We tested 123 metabolites, of which 27 metabolites showed a statistically significant change after Tai Chi training. After Bonferroni correction, 11 metabolites were left significant. Fumaric acid, *L*-aspartic acid and pyroglutamic acid were decreased after Tai Chi training only at 6 months (*P* ≤ 0.033). Downregulation of homocysteine and methionine sulfoxide, and upregulation of azelaic acid were seen in the Tai Chi group both at 6 months (*P* ≤ 0.005) and at 12 months (*P* ≤ 0.032). *L*-malic acid and 3-phosphoglyceric acid were downregulated, while *L*-fucose, adenosine and pipecolic acid were upregulated after Tai Chi training at 12 months (*P* ≤ 0.028) (Additional file 2: Table S5).

We also found several associations between metabolites and clinical presentations. The levels of *L*-malic acid, 3-phosphoglyceric acid and adenosine were associated with the changes of UPDRS total score (*P* ≤ 0.043). The levels of *L*-malic acid, *L*-fucose, and pipecolic acid were associated with the changes of UPDRS-III (*P* ≤ 0.041) (Additional file 2: Table S6).

Pathway analysis showed group differences in arginine biosynthesis both at 6 months (Tai Chi *vs* control: *P* = 0.007; Tai Chi *vs* brisk walking: *P* = 0.006) and at 12 months (Tai Chi *vs* brisk walking: *P* < 0.001) (Additional file 2: Table S7, Additional file 3: Fig. S1-S3). In enrichment analysis, significant group differences were found in urea cycle both at 6 months (Tai Chi *vs* control: *P* = 0.009; Tai Chi *vs* brisk walking: *P* = 0.05) and at 12 months (Tai Chi *vs* brisk walking: *P* < 0.001) (Additional file 2: Table S8, Additional file 3: Fig. S4-S6).

In association analysis, the tricarboxylic acid (TCA) cycle was correlated with BBS (*P* = 0.037), UPDRS total score (*P* = 0.002), and UPDRS-III (*P* = 0.014). Beta oxidation of  very-long-chain fatty acids was relevant to UPDRS total score (*P* = 0.033) and UPDRS-III (*P* = 0.033) (Additional file 2: Table S9).

### HIP2 mRNA levels

In the control group, expression of *HIP2* mRNA showed a tendency of downregulation (*P* = 0.697). Compared to the control group, the *HIP2* mRNA level was elevated after Tai Chi training for 6 months (*P* < 0.001) and 12 months (*P* < 0.001). Tai Chi training showed a tendency to be better in upregulating *HIP2* mRNA level than brisk walking (*P* = 0.277). (Additional file 3: Fig. S7).

We found that the differences in UPDRS total score and UPDRS-III score were associated with the change of *HIP2* mRNA level after Bonferroni correction (*P* < 0.005). The association between the difference in BBS score and the change of *HIP2* mRNA level did not survive after Bonferroni correction (Additional file 2: Table S10).

## Discussion

In this study, we found long-term beneficial effects of Tai Chi in improving balance and other motor symptoms in PD. Tai Chi improved BBS, UPDRS, TUG and step width, indicating its beneficial effects on motor symptoms (including gait and balance). Tai Chi performed better than brisk walking in improving BBS and step width.

More importantly, we explored the mechanisms underlying improvement of motor symptoms of PD after Tai Chi training. fMRI test revealed association between changes of BBS score and the switch of VN, and a positive relationship between improvement of UPDRS and the function of DMN. Plasma cytokines IL-1β, IL-5, IL-7, IL-9, IL-13, MCP-1, MIP-1a, and MIP-1β were relatively downregulated and GM-CSF was upregulated after Tai Chi training. Among them, the downregulation of IL-1β was positively related to the improved BBS scores. The decreased *L*-malic acid and 3-phosphoglyceric acid, and increased adenosine were associated with changes of UPDRS total score in PD after Tai Chi training, while downregulation of *L*-malic acid, and upregulation of *L*-fucose and pipecolic acid were related to changes of UPDRS-III. Arginine biosynthesis, urea cycle, TCA cycle and beta oxidation of very-long-chain fatty acids were also affected by Tai Chi. The *HIP2* mRNA level was significantly elevated after Tai Chi training, and its change was correlated with the changes of UPDRS total score and UPDRS-III score in PD after Tai Chi training.

Our study found significant improvement of motor function (especially gait and balance) in PD patients after Tai Chi training, which was consistent with the results of previous studies [6, 11]. The mechanisms underlying the beneficial effects might be associated with the improved brain network function in PD patients. The VN is composed of bilateral striate and extrastriate visual areas [25]. Visual proprioceptive sensory conflict could influence gait and balance [26]. Visual cues can lessen the vestibular noise and improve personal balance in environment [26]. PD patients with freezing of gait display reduced network connections in VN [27, 28]. Thus, the improved VN function may explain the better performance in BBS in PD after Tai Chi training. We also observed that changes of DMN connectivity were related to the improvement in UPDRS total score and UPDRS-III score. The DMN includes the hippocampus, parahippocampal, fusiform and angular gyrus, the precuneus and the middle temporal gyrus [25]. Precuneus is one of the functional hub regions of DMN, and its interactions with sensorimotor network are positively associated with motor performances [29]. Thus, the improvement of DMN connectivity may explain the improved motor function of PD patients after Tai Chi training, since the connection of precuneus to motor areas might be associated with processes of motor mental imagery and planning [29].

In this study, proinflammatory cytokines were downregulated after Tai Chi training. Among them, the decreased IL-1β was correlated with improved BBS score. Inflammation plays an important role in the pathogenesis and disease progression of PD [15]. A meta-analysis study of inflammatory cytokines in PD has demonstrated significantly higher blood levels of IL-1β compared with healthy controls [30]. IL-1β plays an important role in different neurobiological processes, such as neuroinflammation, neurotoxicity, and host defense. Therefore, this cytokine has been linked to both acute and chronic neurodegenerative conditions [31]. Lipopolysaccharide (LPS) induces PD symptoms by stimulating IL-1β in wild-type animals, suggesting that IL-1β may contribute to the initiation or progression of PD [31]. Decreased IL-1β, which indicates reduced inflammation, may explain the improved BBS performance of PD patients after Tai Chi training. The tendency of decreased IL-1β by Tai Chi training but increased IL-1β by brisk walking training (6-month, *P* = 0.053; 12-month, *P* = 0.096; Additional file 2: Table S3) may explain the superiority of Tai Chi over brisk walking in improving the balance of PD patients.

In addition, the dysregulation of metabolites and metabolic pathways in PD revealed here was mainly associated with amino acid metabolism (pipecolic acid, *L*-fucose, and arginine biosynthesis), energy metabolism (*L*-malic acid, 3-phosphoglyceric acid, urea cycle, TCA cycle and beta oxidation of very-long-chain fatty acids) and neurotransmitter metabolism (adenosine) [32]. *L*-arginine participates in the synthesis of nitric oxide and can affect oxidative stress and energy metabolism, playing a key role in the pathogenesis of PD [33]. Deficiency of TCA cycle enzymes and dysfunction of mitochondria, which regulate neuroinflammation and neurodegeneration, have also been observed in PD [34]. The coupling of adenosine with its specific receptors acts as an upstream neuromodulator for neurotransmitters such as acetylcholine, glutamate, γ-amino-butyric acid, and dopamine that is implicated in the modulation of multiple body functions [35]. Our results indicated improved amino acid metabolism, energy metabolism and neurotransmitter metabolism in PD patients after Tai Chi training.

HIP2 is an E2 ubiquitin-conjugating enzyme related to protein cleavage via the ubiquitin–proteasome system (UPS) pathway [36]. Impaired UPS system is related to protein aggregation, causing inflammation and abnormal oxidation [37]. Reduction of *HIP2* expression leads to impairment of spontaneous motor function and increased vulnerability to dopaminergic degeneration in PD models [21]. In our previous study, *HIP2* mRNA expression was downregulated in 20 PD patients and then elevated after one-year Tai Chi training with improved motor function [21]. Here, the results further confirmed that Tai Chi training could reverse the downregulation of *HIP2* mRNA in a larger PD cohort, and this change was correlated with the improvement of motor function in PD patients after Tai Chi training, suggesting that Tai Chi training can decrease the vulnerability to dopaminergic degeneration in PD.

These lines of fMRI and blood biomarker evidence suggest enhanced brain network function, reduced inflammation, improved amino acid metabolism, energy metabolism and neurotransmitter metabolism, as well as decreased vulnerability to dopaminergic degeneration in PD after Tai Chi training.

To our interest, one-year Tai Chi training decreased the UPDRS-III score compared to baseline (baseline 25.20 ± 17.50 *vs* one-year 19.10 ± 9.56), while the motor symptoms became worse in the brisk walking group (baseline 17.50 ± 7.01 *vs* one-year 23.10 ± 7.81) and the control group (baseline 19.30 ± 4.87 *vs* one-year 30.70 ± 7.35). Since there was no statistical difference in the change of LEDD among the three groups (*P* = 0.939), which could exclude the impact of LEDD, this result indicated that Tai Chi training may have disease-modifying effects on PD.

### Limitations

There were some limitations in our study. First, the number of participants in our study was not large enough. Validity might be lost because of the small sample size. Therefore, larger-size cohort studies are warranted. Second, the dropout rate in the brisk walking and control groups could not be ignored. Since our study observed long-term effects of Tai Chi for one year, the follow-up duration is relatively too long to keep a low drop-out rate. Besides, patients in the Tai Chi class were willing to stick to Tai Chi training since they had benefited from it. Third, we recruited mainly early-stage PD patients. Tai Chi training has a high demand for strength and balance of the lower limbs. If patients have remarkably impaired balance, they would have an increased risk of falling. Thus, early-stage PD patients were recruited to protect them from falling and injuries during training. Besides, early-stage PD patients have less difficulty in mobility and are more likely to meet the requirements for training, which could ensure the quality of training. To further confirm the positive effects of Tai Chi on balance, future studies of Tai Chi training in moderate PD patients are warranted.

## Conclusions

Our study revealed that long-term Tai Chi training improves motor function, especially gait and balance, in PD patients. Enhanced brain network function, reduced inflammation, improved amino acid metabolism, energy metabolism and neurotransmitter metabolism, as well as decreased vulnerability to dopaminergic degeneration may be mechanisms underlying the effects of Tai Chi training.

## Supplementary Information

**Additional file 1**: Protocol.

**Additional file 2: Table S1-S10. Table S1** Clinical assessments of motor symptoms among Tai Chi, Brisk Walking and Control group; **Table S2** The association between clinical improvements and the switch rate of brain networks; **Table S3** Intergroup comparison of cytokines among Tai Chi group, Brisk Walking group and Control group; **Table S4** The association between changes of cytokines and rating scales; **Table S5** Intergroup comparison of metabolites among Tai Chi group, Brisk Walking group and Control group; **Table S6** Multivariate analysis of metabolomics and rating scale; **Table S7** Pathway analysis of metabolites; **Table S8** Enrichment analysis of metabolites; **Table S9** Associations between Pathway/Enrichment Analysis of Metabolomics and clinical presentations among 3 Groups; **Table S10** Associations between *HIP2* mRNA level and clinical presentations in Tai Chi group.

**Additional file 3: Fig. S1**. Pathway analysis in Tai Chi group relative to the Control group from baseline to six-month visit; **Fig. S2** Pathway analysis in Tai Chi group relative to the Control group from six-month visit to one-year visit; **Fig. S3** Pathway analysis in Tai Chi group relative to the Control group from baseline to one-year visit; **Fig. S4** Enrichment analysis in Tai Chi group relative to the Control group from baseline to six-month visit; **Fig. S5** Enrichment analysis in Tai Chi group relative to the Control group from six-month visit to one-year visit; **Fig. S6** Enrichment analysis in Tai Chi group relative to the Control group from baseline to one-year visit; **Fig. S7** Comparison of the change of *HIP2* mRNA level in the 3 groups.

## Data Availability

The datasets used and analyzed during the current study are available from the corresponding author on reasonable request.

## Abbreviations

BBS: Berg Balance scale
DMN: Default mode network
FDR: False discovery rate
fMRI: Functional magnetic resonance imaging
GM-CSF: Granulocyte–macrophage colony stimulating factor
*HIP2*: Huntingtin interaction protein 2
IL: Interleukin
LEDD: Levodopa equivalent daily dosage
MIP: Macrophage inflammatory protein
PD: Parkinson's disease
TUG: Timed up and go
UPDRS: Unified Parkinson’s Disease Rating Scale
UPS: Ubiquitin proteasome system
VN: Visual network
