# Supplementary material for: Mechanisms of motor symptom improvement by long-term Tai Chi training in Parkinson’s disease patients
Source: Transl Neurodegener. 2022 Feb 7;11:6. doi: 10.1186/s40035-022-00280-7 (PMC8819852; doi:10.1186/s40035-022-00280-7)
Supplement: Supplementary file 1 — Additional file 1: Protocol. [file 40035_2022_280_MOESM1_ESM.docx]

**Additional File 1**

**PROTOCOL**

The components in this Supplementary Material document are as follows:

**Part I:** Research Plan

**Part II:** Flow charts and demographics of patients through this research.

**Part III:** Modifications to the Research Plan with description

**Part I: Research Plan**

**Overview**

Base on the aim of our study, we proposed a 1-year randomized controlled single-blind clinical trial with the discovery of mechanism.

This study is a randomized controlled single-blind clinical trial with three intervention arms: Tai Chi, brisk walking and control. PD patients were recruited at baseline, with 1-year intervention (Tai Chi, brisk walking or non-exercise) with 3 timepoint of visit (baseline, 6 months and 12 months), including clinical evaluation, venous blood collection and MRI scan. This study is registered at Chinese Clinical Trial Registry (Registration number: ChiCTR2000036306; Registration date: August 22, 2020).

1. **Participants**

We recruited PD patients whose age were 50-80 years old with Hoehn -Yahr stage 1-2.5. The medication was stable at least 3 months before recruiting and not changed during follow-up unless the change of medication is required according to the disease severity. Our study was approved by Ruijin Hospital Ethic Committee of Shanghai Jiao Tong University School of Medicine at December 04, 2014. PD was diagnosed by movement disorder specialists (SDC, YYT) based on diagnostic criteria brought up by United Kingdom Brain Bank of Parkinson’s disease. During the recruitment period, the new clinical diagnostic criteria of Parkinson’s disease from Movement Disorders Society was brought out at 2015 year. These enrolled patients met both of the criteria. Patients who fitted any following item were excluded:

1) Secondary causes, such as inflammatory, drug-induced, vascular and toxin-induced parkinsonism.

2) Parkinsonism with other neurodegenerative diseases, such as progressive supranuclear palsy, multiple system atrophy, Wilson's disease.

3) Other neurological diseases, such as stroke.

4) Patients who were receiving any other clinical trials or regular exercise protocols.

5) Patients who had fall incidents in the 6 months before recruiting due to safety considerations.

6) Patients whose Mini-Mental State Examination (MMSE) scores were less than 24.

7) Patients who had medical history that did not fit to exercise, such as orthopedics diseases or cardiopulmonary dysfunction.

8) Patients who received education less than 6 years.

9) Patients who could not walk and live independently.

All participants were fully informed and signed written consent forms. This study was approved by the ethic committee of Ruijin Hospital affiliated to Shanghai Jiao Tong University School of Medicine.

1. **Calculation of Sample Size**

G*power software was used to calculate the sample size. The model of “F tests, repeated measures” was introduced. The parameters were: groups = 1, levels = 3, interclass correlation coefficient = 0, spherical hypothesis ε=0.5. So the sample size is 86. The amount of each sample size was 86/3 = 28.7 ≈ 29.

1. **Randomization, blinding and Follow-Up**

After the recruitment, participants were randomly assigned to Tai Chi group, brisk walking group and control group without stratification. The method of randomization is to draw lots.

Since this is an interventional study of movement, we cannot to perform the blinding on patients. Thus, the blinding was to the assessors and the statistician. The ethic committee monitored the whole process of blinding.

12-month exercise intervention was introduced. Assessment was done at baseline, 6 months and 12 months, including clinical evaluation, blood collection and MRI scan. The flow chart of recruitment and follow up was shown in Part II, Figure 1.

1. **Exercise Interventions and Quality Control**

**4.1 Tai Chi**

As for Tai Chi training, standardized Yi Tai Chi was taught by professional Tai Chi coaches in classes: *Qishi* (“Starting Posture”), *Shangsanbu* (“Twist Step”), *Yema Fenzong* (“Part the Wild Horse's Mane on Both Side”), *Jingang Daozhui* (“Buddha’s warrior attendant pounds mortar”), *Shoushi* (“Closing Posture”). Patients participated in this class were trained for one year, twice a week, 60 min per time. PD patients whose attendance rate less than 75% were excluded.

The detailed Tai Chi protocols are as follows:

***Qishi* (“Starting Posture”)**: The two legs stand side by side. Put hands beside with thighs. The weight is in the middle. Then move the weight to the right leg, meanwhile, bend the right knee slowly and lift the right knee slowly. Step to the left, let the toes touching the ground. Then heel down. Stand the right leg slowly and shift the weight from the right leg to the center. Keep the abdomen in, sink the body and keep the chest in with twisting arms inward. Keep sinking, relax shoulders and elbows. Keep the fingers to the knee, with the fingertips downward and perpendicular to the ground. Keep the abdomen in, sink and inhale, with lifting the hands slowly in front of the body. Move the shoulders to the backward, then sink, relax shoulders and elbows. Slightly lean the body forward. Keep sinking with fall the hands slowly to the thighs. Then back to the first position.

***Shangsanbu* (“Twist Step”)**: Sink the body with falling the palm, turn right, twist the left hand inward and the right hand outward. Move the weight from the center to the left leg. Keep sinking. Swing the toes of the left foot 90 degrees. Turn right, keep twisting the left hand inward and twisting the right hand outward. Draw an arc in the air to the upward and keep sinking the body. Shift the left hip inward, sink and slightly tilt the body to the left. Keep sinking and move the hands upward. Turn left, make the right leg one step forward. The left heel moves beside the right foot, with the sinking palm facing forward and upward. Keep sinking, turn left, twist the right hand inward and twisting the left hand outward, move the weight to the right leg. Keep sinking, and turn left slightly, twisting the right hand inward and the left hand outward. Draw an arc in the air to the upward and keep sinking the body. Shift the right hip inward, sink and slightly tilt the body to the right. Keep sinking and move the hands upward. Turn right, make the left leg one step forward, with the sinking palm facing forward and upward. Keep sinking, turn right, twist the left hand inward the right hand outward, move the weight to the left leg. Keep sinking, and turn right slightly, twisting the left hand inward and the right hand outward. Draw an arc in the air to the upward and keep sinking the body. Shift the left hip inward, sink and slightly tilt the body to the left. Keep sinking and move the hands upward. Turn left, make the right leg one step forward with the sinking palm facing forward and upward.

***Yema Fenzong* (“Part the Wild Horse's Mane on Both Side”)**: Draw the abdomen in and fall the both hands. Inhale with both hands move upward. Draw the chest in, make the elbows open to both sides of the body. Keep sinking, open the palms facing backwards, tilt the body. Twist both hands inwards and draw arcs forward. Make the right foot one step backward. Put both hands together, back-to-back. Relax, twist both hands outwards to the palms facing each other. Turn right slightly, with twisting the right hand outwards and the left hand inwards. Hook the left foot, keep the wrist together, and the body goes back to the straight. Turn right, keep the right foot inward with both hands falling down to the inner side of the right knee. Sink, turn left, move the weight to the right leg, and put both hands in front of the left hip. Sink, turn right slightly. Twist the left hand inwards and the right arm outwards. Separate both hands and keep sinking. Sink, turn left slightly, make the left palm facing up. Sink, turn right slightly, keep the chest in. Twist both hands inward. Keep turning right, and make the left hand go for the right hand. Sink, and bring hands down. Turn right slightly, and twist the left hand inwards, with moving the weight to the left leg. Sink, turn left. The body goes back to the straight, with both hands moving to the left. Sink, turn right slightly, and face the right palm forward. Make both hands ward off to the left front. Relax the left arms and elbows. Turn both hands facing to each other. Sink, turn left. Move the wright to the right leg and keep flipping both hands. Sink, turn right, move the left hand to the right armpit. Sink, turn right. Move the weight to the left leg. Twisting the falling left hands outwards to the front of the right hip. Sink, turn left slightly. Twist the left arm outwards. Separate both hands and sink.

***Jingang Daozhui* (“Buddha’s warrior attendant pounds mortar”)**: Sink, turn right slightly. Twist the left arm inwards and the right hand outwards. Move the body to the straight position. Flip the right hand facing upwards. The left hand put to the chest, perpendicular to the ground. Keep sinking the body, with the falling of the left hand. Turn left, Twist the falling left hand inwards, with swinging the right heel outwards and flip the right hand inwards. Sink the body, turn left and put the left toes to the 45 degrees. Make the right leg one step forward. Put the right hand in front of the right hip. The left hand falls down to the inner left thigh. Sink the body, make the right hand fall. Turn left and flip the left palm forward. Turn right, draw arcs with both hands left, upwards and forwards. Make both hands to the chest, perpendicular to the ground. Sink the body, push the right palm forward, and make left palm to the chest, perpendicular to the ground. Sink the body, move the weight to the right leg. Make the left leg one step forward and push. Make the body go straight. Let the right elbow hit to the right. Turn right and put the right left inwards. Twisting the right hand inwards till the palm facing outwards. Fall the left hand to the palm facing downwards. Sink the body, turn right, and draw the right hand backwards. Sink the body, turn left, and push the right hand to the right backwards. Relax the shoulder and the elbow with moving outwards, till the palms draw arcs to the outer side of the right thigh. Move the right foot one step forward. Bring the right hand forward, and the left hand meets to the below of the right arm. Sink the body, the right forearm lifts upwards. Tilt forwards slightly, and make the left hand to the body, flip the hand. The right meets the left hand slightly inwards. Sink the body, both hands relax and fall, with moving the right foot inwards. Lift the right foot, make the right palm to the fist, and lift. Hit the right foot to the ground, meanwhile the right fist hits the left palm.

***Shoushi* (“Closing Posture”)**: Sink the body and meanwhile open both hands to the body side, with the palm facing each other. Move the body upwards, with both hands facing down and moving to the same height with the shoulder. Sinking the body and flip both hands to the front. Draw an arc to above the head with palms facing each other. Keep sinking. Relax shoulders and elbows. Open the chest with elbows stretching to both sides. Keep sinking with palms moving and facing downwards. Move the body upwards, and keep palms moving and facing downwards to the abdomen. Draw the abdomen in and keep sinking. Make both hands fall down and move the palms inwards. Inhale and move both hands outwards. Move the weight to the right leg and keep sinking. Draw the chest in. Draw arcs of both hands forward, with moving the left foot inwards. Relax, closing both hands to the pelvis. Get up slowly. And put both hands naturally to both sides of the body. Relax.

**4.2 Brisk Walking**

As for brisk walking training, a moderate intensity aerobic exercise method was adopted: twice a week, 60 min/time walk monitored by sport watches in heart rate, distance and speed etc.. The heart rate during walking should be 50% - 60% maximum heart rate. We used the following calculation to get maximum heart rate: 220 – age. Data from sport watches were used to monitor the quality of brisk walking.

**4.3 Control**

We did not give any exercise interventions to the control group.

1. **Evaluation**

**5.1 Motor symptom assessment by rating scales**

The following rating scales were used to assess the status of patients at “on” periods at movement disorders clinic.

(1) Overall assessment of PD: Unified Parkinson's Disease Rating Scale (UPDRS) and Hoehn -Yahr stage;

(2) Motor assessment: Berg Balance scale (BBS), UPDRS-III and Timed Up and Go test (TUG) were used to assess severity of balance, gait and motor symptoms.

All patients were assessed by experienced doctors (PH, YHC, GL, SSC), who all passed the tests for proving the interrater reliability.

**5.2 Three-dimensional gait test**

Gait was tested by T40 10 motion capture cameras (Vicon Inc.) at Shanghai Key Laboratory for Bone and Joint Diseases of Shanghai Institute of Orthopedics and Traumatology. The following parameters were recorded: cadence, double support, foot off, limp index, opposite foot contact, opposite foot off, single support, step length, step time, step width, stride length, stride time and walking speed for both sides. We recorded 6 times of walking.

1. **Outcomes**

We regarded total score of BBS of PD as the primary outcome. Total score of UPDRS, score of UPDRS-III, mean time of TUG, and spatiotemporal parameters of gait analysis were also acquired (including cadence, step length, step width, stride length, and velocity for both sides).

**6.1 FMRI data acquisition and processing**

FMRI data was collected by the 3.0T MRI machine (Siemens Inc., Munich, Germany) at Institute of Neuroscience of Shanghai Institute for Biological Sciences, Chinese Academy of Sciences. During the scan, subjects were asked to remain motionless and awake with their eyes closed. For each participant, 300 functional images were collected. The repetition time (TR) of T1 image was 2300ms, the echo time (TE) of T1 image was 3ms, flip angle=9 degrees, 176 slices, matrix=256×256, voxel size was 1.0×1.0×1.0mm^3^. The parameters for BOLD image were as follows: TR: 2000ms, TE: 30ms, flip angle=62 degrees, 56 slices, matrix=80×80, voxel size: 3.0×3.0×3.0mm^3^.

DPABI (version 4.3_200301) was used for the preprocessing of fMRI data. GroupICAT (v4.0a, The MIND Research Network) was used for group-level independent component analysis. Functional network dynamic connectivity was analyzed and switch rates of brain networks were calculated according to the pervious methods [24].

The first 10 frames were discarded, and the remaining volumes were slice-timing corrected. Then they were realigned to the first image to correct for head motion. Individuals with the translation and rotation head motion parameters larger than 3 mm or 3 o were excluded. Then functional images were co-registered to the corresponding T1 images. The co-registered T1 images were segmented, then the Diffeomorphic Anatomical Registration Through Exponentiated Lie algebra (DARTEL) tool was employed to spatially normalize both the T1 images and the functional images into MNI space. Finally，functional images were resampled to 3 × 3 × 3 mm^3^ voxels. Smooth was done using 4mm-full-width at half-maximum (FWHM).

**6.2 Cytokine detection**

Plasma cytokines were tested by Luminex (MAGPIX, Luminex Corporation) with kits from Bio Rad (#M500KCAF0Y). Results are expressed as pg/ml. We tested the following cytokines:

1. Interleukin (IL) family: IL-1β, IL-1RA, IL-2, IL-4, IL-5, IL-6, IL-7, IL-9, IL-10, IL-12, IL-13, IL-15, IL-17A ;
2. Colony stimulating factor (CSF) family: granulocyte-macrophage CSF (GM-CSF), granulocyte CSF (G-CSF);
3. Interferon (IFN) family: IFN-γ;
4. Chemokines: CXCL8/IL-8, CXCL10/IFN-induced protein-10 (IP-10), CCL2/ monocyte chemoattractant protein-1 (MCP-1), CCL3/ macrophage inflammatory protein-1α (MIP-1α), CCL4/ macrophage inflammatory protein-1β (MIP-1β), CCL5/ regulated upon activation normal T cell expressed and secreted factor (RANTES), CCL11/eotaxin;
5. Tumor necrosis factor (TNF) family: TNF-α;
6. Other cytokines: vascular endothelial growth factor (VEGF), fibroblast growth factor-basic (Basic FGF), platelet derived growth factor– BB (PDGF-BB).

**6.3 Metabolomics**

The non-targeted metabolomics was performed, aiming to assess global metabolite profiles in PD. The XploreMET™ platform (Metabo-Profile, Shanghai, China) was used to perform the non-targeted metabolomics.

**6.4 The detection of Huntingtin interaction protein 2 (*HIP2*) mRNA**

QIAamp® RNA Blood Mini Kit was used to extract mRNA from patient’s blood samples. The RNA was reversed and detected by qPCR method according to the method provided by Su et al. [21].

1. **The Discovery of Mechanisms**

We also collected the neuroimaging data and venous blood of patients for discovering the possible mechanisms. We assessed the changes of PD patients after exercise training by functional magnetic resonance imaging (fMRI), *HIP2* mRNA levels, plasma cytokines and metabolomics, and then analyzed the relationship between those changes and the clinical symptoms, trying to find the mechanism of brain network, cytokines and metabolomics associated with exercise.

1. **Statistical Analysis**

R (version 3.5.1) and RStudio (version 1.1463) were used to perform statistical analysis. Packages stats (version 3.5.1), base (version 3.5.1), spinds (version 2.2.0), lars (version 1.2), nlme (version 3.1-145), plyr (version 1.8.5), PGEE (version 1.5), ggplot2 (version 3.2.1), gee (version 4.13-20), bnlearn (version 4.4), elasticnet (version 1.1.1), survival (version 2.43-3) and survminer (version 0.4.3) were introduced into statistical analysis. MATLAB R2018a (version 9.4.0.813654, MathWorks, Inc.) was used into the analysis of fMRI.

All analysis about primary and secondary outcomes were conducted on an intention-to-treat basis. Analysis of variance (ANOVA) were used into comparing at numerical demographic information. Pearson Chisq-Square test or Fisher test were used into analysis at categorical demographic information. Shapiro-Wilk normality test was used to assess the normality of variables. Independent-sample t-tests (with 95% confidence intervals) were used to compare group means. Data were presented in the way of between-group differences. The longitudinal effects of self-changes were calculated using repeated measures ANOVA.

**7.1 Clinical data analysis**

Generalized estimating equations (GEE) was used to assess the association of longitudinal data of rating scales. Data after log transformation were used to perform GEE because of they did not follow Gaussian distribution. We adjusted age, gender and disease duration when analysis. Education was also adjusted when MMSE and PDCRS were analyzed. LEDD and Hoehn -Yahr stage were not into adjusted due to collinearity with disease duration. Bonferroni correction were used in performing multiple corrections.

**7.2 FMRI analysis**

In the analysis of fMRI, we calculated the associations between the changes of rating scales (UPDRS, TUG, BBS) and the changes of switch rates. Firstly, the least absolute shrinkage and selection operator (LASSO) was used to the selection of switch rates to avoid overfitting. Then, log transform was introduced to the selected switch rates which did not obey normality. Lastly, linear regression was performed among the selected switch rates. Besides, Bayesian belief network (BBN) was also introduced to confirm the causality of the associations. FDR correction was adopted. We regarded alpha as 0.05 for statistically significant.

**7.3 Plasma Cytokine analysis**

We analyzed cytokine levels with mixed effect regression using restricted maximum likelihood method because personal heterogeneity influenced cytokine levels. Bonferroni correction were used in performing multiple corrections. The penalized generalized estimating equation (PGEE) was adopted for the feature selection of three-group comparison with 3 visits.

**7.4 Plasma metabolomics analysis**

The ratio was calculated between each 2 visits (six-month visit/baseline, one-year visit/six-month visit, one-year visit/baseline), aiming to investigate the change of the metabolites. The principal component analysis and orthogonal partial least squares discrimination analysis were used using SIMCA-P software (Umetrics, version 14.1). Those metabolites whose VIP score was above 1.5 were selected into analysis. PGEE was adopted for the feature selection of three-group comparison with 3 visits. Pathway analysis were performed using MetaboAnalyst 4.0 platform. Enrichment analysis were performed using MetaboAnalyst 4.0 platform referenced with the small molecule pathway database (SMPDB). False discovery rate (FDR) correction was used to perform multiple correction. Since the personal heterogeneity influenced the metabolite levels, the mixed effect regression using restricted maximum likelihood method was adopted to the selected metabolites.

**7.5 The analysis of *HIP2* mRNA changes**

The relative expression of *HIP2* mRNA in Tai Chi group and brisk walking group was calculated compared with controls. Mixed effect regression using restricted maximum likelihood method was used to calculate the changes among each visit. The relative ratio to baseline was adopted to plot.

**Part II: Flow charts and demographics of patients through this research**


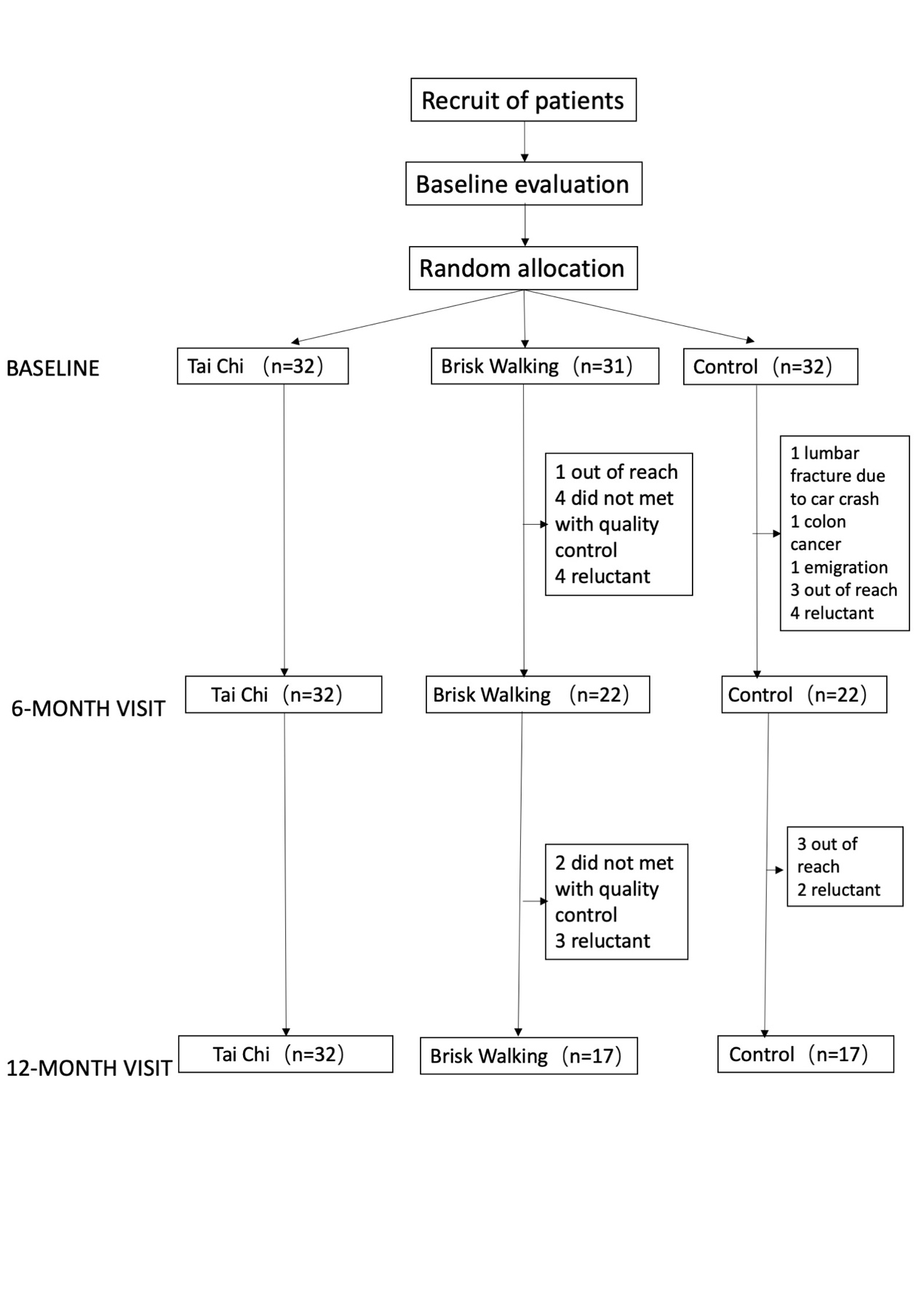


**Figure 1 The flow chart of the recruitment among Tai Chi, brisk walking and control group**

**Table 1 Demographic information of all the subjects**

|  | Tai Chi Group  (N = 32) | Brisk Walking Group  (N = 31) | Control Group  (N = 32) | *p* value |
| --- | --- | --- | --- | --- |
| Gender, Female (N (%)) | 15 (46.88) | 9 (29.03) | 13 (40.63) | 0.339 |
| Age at baseline (mean ± SD) | 62.7 (5.51) | 61.5 (5.53) | 62.8 (6.14) | 0.948 |
| Education, years (mean ± SD) | 13.60 (2.71) | 12.9 (2.51) | 12.40 (3.30) | 0.099 |
| History of Hypertension (N (%)) | 7 (21.88) | 3 (9.68) | 5 (15.63) | 0.414 |
| History of Diabetes mellitus (N (%)) | 1 (3.13) | 0 (0.00) | 1 (3.13) | 1.000 |
| History of smoking (N (%)) | 2 (6.25) | 2 (6.25) | 2 (6.25) | 1.000 |
| Family History (N(%)) | 8 (25.00) | 5 (16.13) | 6 (18.75) | 0.663 |
| Tremor dominant (N (%)) | 22 (68.75) | 22 (70.97) | 17 (53.12) | 0.271 |
| Disease duration (mean ± SD) | 5.91 (4.01) | 5.13 (3.11) | 5.44 (3.96) | 0.615 |
| Hoehn – Yahr staging (N (%))  1.0  1.5  2.0  2.5 | 9 (28.13)  5 (15.63)  13 (40.63)  5 (15.63) | 8 (25.81)  7 (22.58)  13 (41.94)  3 (9.68) | 1 (3.13)  11 (34.38)  13 (40.63)  7 (21.88) | 0.113 |
| LEDD at baseline (mean ± SD) | 326 (197) | 260 (174) | 347 (109) | 0.800 |
| $\Delta$LEDD (mean ± SD) | 56.33 (91.68) | 39.71 (83.30) | 57.21 (107.24) | 0.939 |
| LEDD, levodopa equivalent daily dosage; N, number; SD, standard deviation  $\Delta$LEDD: LEDD at 12 months minus LEDD at baseline | | | | |

**Part III: Modifications**

Basically, there were no significant changes made in the original research protocol as we reported. The following changes were done. All modifications were approved by the ethics committee and the meeting of authors.

**Change 1: (Jan. 1^st^, 2016)**

Patients met both UK brain bank diagnostic criteria [22] and MDS diagnostic criteria [23]. The MDS diagnostic criteria were not published when we got the approval from Ruijin Hospital Ethic Committee, Shanghai Jiao Tong University School of Medicine at December 04, 2014. However, when we put the protocol into effect, the MDS diagnostic criteria of PD were published at 2015 year. Thus, we enrolled PD patients both met UK brain bank diagnostic criteria and MDS diagnostic criteria from 2016 year.

**Change 2: (Aug. 22^nd^, 2020)**

We noticed the inappropriate information were submitted due to inappropriate operation in 2016 (register number: ChiCTR-OPC-16008074). We mis-clicked “interventional” as “observational” when we registered at the Chinese Clinical Trial website. We noticed this incorrect information in 2020. So we re-registered a new project called “A prospective cohort study of exercise rehabilitation in the treatment of Parkinson's disease and its mechanism” after the approval of our ethic committee (register number: ChiCTR2000036306).
