# Supplementary material for: Mechanisms of motor symptom improvement by long-term Tai Chi training in Parkinson’s disease patients
Source: Transl Neurodegener. 2022 Feb 7;11:6. doi: 10.1186/s40035-022-00280-7 (PMC8819852; doi:10.1186/s40035-022-00280-7)
Supplement: Supplementary file 2 — Additional file 2: Table S1-S10. Table S1 Clinical assessments of motor symptoms among Tai Chi, Brisk Walking and Control group; Table S2 The association between clinical improvements and the switch rate of brain networks; Table S3 Intergroup comparison of cytokines among Tai Chi group, Brisk Walking group and Control group; Table S4 The association between changes of cytokines and rating scales; Table S5 Intergroup comparison of metabolites among Tai Chi group, Brisk Walking group and Control group; Table S6 Multivariate analysis of metabolomics and rating scale; Table S7 Pathway analysis of metabolites; Table S8 Enrichment analysis of metabolites; Table S9 Associations between Pathway/Enrichment Analysis of Metabolomics and clinical presentations among 3 Groups; Table S10 Associations between HIP2 mRNA level and clinical presentations in Tai Chi group. [file 40035_2022_280_MOESM2_ESM.docx]

**Table S1 Clinical assessments of motor symptoms among Tai Chi, Brisk Walking and Control groups**

| Measure | Tai Chi * | Brisk Walking * | Control * | Between-group difference in longitudinal mean change# | | | | | | | |  |
| --- | --- | --- | --- | --- | --- | --- | --- | --- | --- | --- | --- | --- |
|  |  |  |  |  | Tai Chi v.s. Control | *P* value | Brisk Walking v.s. Control | *P* value | Tai Chi v.s. Brisk walking | *P* value | *P* value of 3-group comparison | |
| **BBS** |  |  |  |  |  |  |  |  |  |  |  | |
| Baseline | 49.20 (3.64) | 52.80 (2.54) | 52.90 (3.23) |  |  |  |  |  |  |  |  | |
| 6-month visit | 50.80 (4.91) | 53.20 (2.22) | 53.20 (3.83) | 6-month visit – baseline | -1.26 (-3.26, 0.73) | 0.006 | -1.03 (-2.65, 0.59) | 0.96 | -0.24 (-2.5, 2.03) | 0.005 | 0.005 | |
| 1-year visit | 51.70 (4.27) | 53.10 (1.54) | 52.50 (3.28) | 1-year visit – 6-month visit | -1.64 (-2.98, -0.31) | 0.29 | -1.06 (-1.98, -0.13) | 0.63 | -0.59 (-2.13, 0.95) | 0.13 | 0.24 | |
|  |  |  |  | 1-year visit – baseline | -2.91 (-5.01, -0.81) | 0.044 | -2.08 (-3.57, -0.6) | 0.75 | -0.82 (-3.38, 1.74) | 0.022 | 0.035 | |
|  |  |  |  | *P* value among 3 visits |  | 0.018 |  | 0.55 |  | 0.005 | 0.016 | |
| **UPDRS - total score** |  |  |  |  |  |  |  |  |  |  |  | |
| Baseline | 40.00 (16.90) | 29.10 (8.33) | 28.10 (6.94) |  |  |  |  |  |  |  |  | |
| 6-month visit | 32.80 (13.70) | 26.80 (9.35) | 34.20 (5.91) | 6-month visit – baseline | 13.37 (8.63, 18.11) | 0.861 | 5.07 (1.29, 8.86) | 0.14 | 8.29 (2.72, 13.87) | 0.402 | 0.98 | |
| 1-year visit | 30.10 (13.70) | 31.20 (8.16) | 41.70 (8.21) | 1-year visit – 6-month visit | 10.48 (7.64, 13.32) | <0.001 | 7.01 (4.49, 9.53) | 0.001 | 3.47 (0.05, 6.89) | 0.417 | <0.001 | |
|  |  |  |  | 1-year visit – baseline | 23.85 (18.46, 29.23) | 0.015 | 12.08 (7.84, 16.32) | 0.022 | 11.76 (5.32, 18.21) | 0.921 | 0.03 | |
|  |  |  |  | *P* value among 3 visits |  | 0.030 |  | 0.010 |  | 0.956 | 0.035 | |
| **UPDRS Part III** |  |  |  |  |  |  |  |  |  |  |  | |
| Baseline | 25.20 (12.50) | 17.50 (7.01) | 19.30 (4.87) |  |  |  |  |  |  |  |  | |
| 6-month visit | 21.50 (10.30) | 17.00 (7.11) | 23.50 (5.52) | 6-month visit – baseline | 7.89 (4.54, 11.24) | 0.227 | 3.19 (0.85, 5.52) | 0.013 | 4.71 (0.4, 9.01) | 0.369 | 0.33 | |
| 1-year visit | 19.10 (9.56) | 23.10 (7.81) | 30.70 (7.35) | 1-year visit – 6-month visit | 10.02 (7.78, 12.26) | <0.001 | 8.43 (6.23, 10.64) | 0.004 | 1.59 (-1.42, 4.6) | 0.24 | <0.001 | |
|  |  |  |  | 1-year visit – baseline | 17.91 (13.96, 21.87) | <0.001 | 11.62 (8.4, 14.84) | 0.008 | 6.29 (1.29, 11.3) | 0.711 | 0.002 | |
|  |  |  |  | *P* value among 3 visits |  | <0.001 |  | <0.001 |  | 0.786 | <0.001 | |
| **TUG (s)** |  |  |  |  |  |  |  |  |  |  |  | |
| Baseline | 12.23 (2.74) | 9.38 (1.51) | 10.56 (1.98) |  |  |  |  |  |  |  |  | |
| 6-month visit | 11.35 (3.43) | 8.98 (1.59) | 9.91 (1.52) | 6-month visit – baseline | 0.23 (-1.1, 1.56) | 0.017 | 0.47 (-0.79, 1.74) | 0.018 | -0.24 (-1.21, 0.72) | <0.001 | 0.066 | |
| 1-year visit | 10.45 (3.18) | 9.31 (1.00) | 10.30 (1.00) | 1-year visit – 6-month visit | 1.35 (0.54, 2.15) | 0.42 | 1.24 (0.52, 1.96) | 0.005 | 0.11 (-0.75, 0.96) | 0.008 | 0.29 | |
|  |  |  |  | 1-year visit – baseline | 1.58 (0.17, 2.98) | 0.011 | 1.71 (0.43, 2.99) | 0.004 | -0.14 (-1.21, 0.94) | <0.001 | 0.066 | |
|  |  |  |  | *P* value among 3 visits |  | 0.047 |  | 0.006 |  | <0.001 | 0.040 | |
| **GAIT PARAMETERS** | | | | | | | | | | | |  |
| **Cadence (step/min), severer part** |  |  |  |  |  |  |  |  |  |  |  | |
| Baseline | 103.35 (17.16) | 105.80 (11.00) | 105.86 (12.64) |  |  |  |  |  |  |  |  | |
| 6-month visit | 102.91 (19.83) | 103.80 (10.91) | 107.45 (10.82) | 6-month visit – baseline | -0.69 (-9.5, 8.12) | 0.759 | 0.48 (-7.55, 8.52) | 0.896 | -1.18 (-8.48, 6.12) | 0.792 | 0.672 | |
| 1-year visit | 102.86 (18.88) | 102.20 (12.55) | 112.00 (6.56) | 1-year visit – 6-month visit | 3.38 (-16.83, 23.6) | 0.546 | -4.48 (-17.93, 8.97) | 0.31 | 7.86 (-9.99, 25.71) | 0.645 | 0.593 | |
|  |  |  |  | 1-year visit – baseline | 8.08 (-8.26, 24.43) | 0.708 | -4.84 (-15.01, 5.32) | 0.611 | 12.93 (-13.77, 39.62) | 0.926 | 0.645 | |
| **Step length (m), severer part** |  |  |  |  |  |  |  |  |  |  |  | |
| Baseline | 0.55 (0.05) | 0.57 (0.06) | 0.52 (0.12) |  |  |  |  |  |  |  |  | |
| 6-month visit | 0.56 (0.05) | 0.57 (0.06) | 0.56 (0.06) | 6-month visit – baseline | 0 (-0.05, 0.04) | 0.426 | -0.01 (-0.06, 0.03) | 0.158 | 0.01 (-0.04, 0.07) | 0.166 | 0.785 | |
| 1-year visit | 0.57 (0.05) | 0.58 (0.05) | 0.59 (0.06) | 1-year visit – 6-month visit | 0.01 (-0.08, 0.1) | 0.931 | 0 (-0.06, 0.06) | 0.415 | 0.01 (-0.09, 0.11) | 0.756 | 0.794 | |
|  |  |  |  | 1-year visit – baseline | -0.02 (-0.11, 0.06) | 0.487 | -0.01 (-0.06, 0.05) | 0.173 | -0.02 (-0.15, 0.11) | 0.317 | 0.882 | |
| **Step width (m), severer part** |  |  |  |  |  |  |  |  |  |  |  | |
| Baseline | 0.13 (0.04) | 0.14 (0.02) | 0.15 (0.04) |  |  |  |  |  |  |  |  | |
| 6-month visit | 0.12 (0.03) | 0.15 (0.03) | 0.16 (0.03) | 6-month visit – baseline | 0.01 (-0.01, 0.04) | 0.002 | 0 (-0.02, 0.02) | 0.101 | 0.01 (-0.01, 0.03) | 0.03 | 0.0004 | |
| 1-year visit | 0.13 (0.03) | 0.14 (0.03) | 0.12 (0.08) | 1-year visit – 6-month visit | -0.02 (-0.06, 0.02) | 0.022 | -0.02 (-0.04, 0.01) | 0.06 | 0 (-0.08, 0.07) | 0.115 | 0.001 | |
|  |  |  |  | 1-year visit – baseline | 0 (-0.05, 0.04) | 0.0003 | 0 (-0.03, 0.02) | 0.041 | 0 (-0.08, 0.08) | 0.031 | 0.0002 | |
| **Stride length (m), severer part** |  |  |  |  |  |  |  |  |  |  |  | |
| Baseline | 1.08 (0.1) | 1.15 (0.09) | 1.05 (0.21) |  |  |  |  |  |  |  |  | |
| 6-month visit | 1.13 (0.1) | 1.19 (0.11) | 1.12 (0.08) | 6-month visit – baseline | -0.06 (-0.14, 0.02) | 0.219 | -0.03 (-0.11, 0.04) | 0.111 | -0.03 (-0.13, 0.07) | 0.074 | 0.935 | |
| 1-year visit | 1.1 (0.1) | 1.18 (0.11) | 1.18 (0.01) | 1-year visit – 6-month visit | 0.04 (-0.07, 0.15) | 0.915 | 0.04 (-0.03, 0.12) | 0.143 | 0 (-0.12, 0.11) | 0.115 | 0.545 | |
|  |  |  |  | 1-year visit – baseline | -0.07 (-0.2, 0.05) | 0.191 | 0.03 (-0.05, 0.12) | 0.123 | -0.11 (-0.3, 0.09) | 0.076 | 0.795 | |
| **Velocity (m/s), severer part** |  |  |  |  |  |  |  |  |  |  |  | |
| Baseline | 0.94 (0.21) | 1.01 (0.12) | 0.93 (0.23) |  |  |  |  |  |  |  |  | |
| 6-month visit | 0.98 (0.21) | 1.02 (0.12) | 1 (0.13) | 6-month visit – baseline | -0.06 (-0.15, 0.03) | 0.51 | -0.03 (-0.11, 0.05) | 0.15 | -0.03 (-0.11, 0.04) | 0.441 | 0.602 | |
| 1-year visit | 0.95 (0.23) | 1.01 (0.14) | 1.1 (0.06) | 1-year visit – 6-month visit | 0.08 (-0.06, 0.22) | 0.73 | 0.01 (-0.1, 0.11) | 0.823 | 0.07 (-0.11, 0.26) | 0.852 | 0.627 | |
|  |  |  |  | 1-year visit – baseline | 0.03 (-0.16, 0.21) | 0.572 | -0.02 (-0.14, 0.1) | 0.205 | 0.04 (-0.16, 0.25) | 0.608 | 0.671 | |
| **Cadence (step/min), milder part** |  |  |  |  |  |  |  |  |  |  |  | |
| Baseline | 103.04 (16.55) | 104.56 (9.25) | 105.31 (10.88) |  |  |  |  |  |  |  |  | |
| 6-month visit | 104.78 (17.50) | 104.15 (8.78) | 107.71 (11.93) | 6-month visit – baseline | -2.02 (-9.57, 5.54) | 0.947 | -0.63 (-7.32, 6.06) | 0.711 | -1.39 (-9.32, 6.54) | 0.869 | 0.895 | |
| 1-year visit | 103.83 (17.78) | 102.91 (12.56) | 114.00 (5.29) | 1-year visit – 6-month visit | 6.96 (-4.33, 18.25) | 0.65 | -2.39 (-10.41, 5.64) | 0.311 | 9.34 (-7.69, 26.37) | 0.505 | 0.703 | |
|  |  |  |  | 1-year visit – baseline | 7.68 (-5.73, 21.09) | 0.894 | -4.33 (-13.37, 4.71) | 0.473 | 12.01 (-9.78, 33.8) | 0.678 | 0.851 | |
| **Step length (m), milder part** |  |  |  |  |  |  |  |  |  |  |  | |
| Baseline | 0.52 (0.07) | 0.58 (0.05) | 0.55 (0.07) |  |  |  |  |  |  |  |  | |
| 6-month visit | 0.56 (0.09) | 0.6 (0.05) | 0.56 (0.04) | 6-month visit – baseline | -0.03 (-0.1, 0.03) | 0.268 | -0.03 (-0.09, 0.04) | 0.006 | 0 (-0.06, 0.06) | 0.066 | 0.236 | |
| 1-year visit | 0.54 (0.06) | 0.58 (0.04) | 0.59 (0.06) | 1-year visit – 6-month visit | 0.01 (-0.09, 0.1) | 0.421 | 0.02 (-0.04, 0.09) | 0.124 | -0.02 (-0.12, 0.09) | 0.166 | 0.388 | |
|  |  |  |  | 1-year visit – baseline | -0.01 (-0.1, 0.07) | 0.486 | -0.01 (-0.07, 0.04) | 0.013 | 0 (-0.06, 0.05) | 0.073 | 0.358 | |
| **Step width (m), milder part** |  |  |  |  |  |  |  |  |  |  |  | |
| Baseline | 0.12 (0.03) | 0.14 (0.03) | 0.15 (0.04) |  |  |  |  |  |  |  |  | |
| 6-month visit | 0.12 (0.03) | 0.15 (0.03) | 0.16 (0.02) | 6-month visit – baseline | 0.02 (-0.01, 0.04) | <0.001 | 0.01 (-0.02, 0.03) | 0.096 | 0.01 (-0.02, 0.03) | 0.004 | 0.0004 | |
| 1-year visit | 0.13 (0.03) | 0.15 (0.03) | 0.11 (0.07) | 1-year visit – 6-month visit | -0.03 (-0.08, 0.01) | 0.03 | -0.01 (-0.04, 0.02) | 0.32 | -0.02 (-0.1, 0.05) | 0.11 | 0.0002 | |
|  |  |  |  | 1-year visit – baseline | -0.01 (-0.06, 0.04) | 0.0002 | 0 (-0.03, 0.03) | 0.011 | -0.01 (-0.08, 0.05) | 0.111 | 0.0001 | |
| **Stride length (m), milder part** |  |  |  |  |  |  |  |  |  |  |  | |
| Baseline | 1.07 (0.13) | 1.18 (0.09) | 1.03 (0.29) |  |  |  |  |  |  |  |  | |
| 6-month visit | 1.14 (0.12) | 1.17 (0.08) | 1.12 (0.06) | 6-month visit – baseline | -0.07 (-0.17, 0.04) | 0.211 | -0.08 (-0.18, 0.02) | 0.015 | 0.02 (-0.07, 0.1) | 0.012 | 0.308 | |
| 1-year visit | 1.09 (0.15) | 1.17 (0.07) | 1.13 (0.07) | 1-year visit – 6-month visit | 0.03 (-0.14, 0.2) | 0.987 | 0.07 (-0.04, 0.18) | 0.079 | -0.04 (-0.2, 0.11) | 0.425 | 0.92 | |
|  |  |  |  | 1-year visit – baseline | -0.09 (-0.26, 0.08) | 0.186 | -0.02 (-0.13, 0.1) | 0.012 | -0.08 (-0.24, 0.08) | 0.05 | 0.275 | |
| **Velocity (m/s), milder part** |  |  |  |  |  |  |  |  |  |  |  | |
| Baseline | 0.93 (0.23) | 1.03 (0.14) | 0.94 (0.19) |  |  |  |  |  |  |  |  | |
| 6-month visit | 1.00 (0.20) | 1.01 (0.12) | 1.01 (0.12) | 6-month visit – baseline | -0.06 (-0.16, 0.03) | 0.679 | -0.07 (-0.16, 0.02) | 0.118 | 0.01 (-0.07, 0.08) | 0.312 | 0.832 | |
| 1-year visit | 0.95 (0.24) | 1 (0.11) | 1.08 (0.04) | 1-year visit – 6-month visit | 0.08 (-0.06, 0.22) | 0.797 | 0.03 (-0.08, 0.13) | 0.908 | 0.06 (-0.15, 0.27) | 0.983 | 0.718 | |
|  |  |  |  | 1-year visit – baseline | -0.01 (-0.18, 0.17) | 0.705 | -0.06 (-0.17, 0.06) | 0.15 | 0.05 (-0.11, 0.21) | 0.468 | 0.845 | |
| BBS, Berg balance scale; TUG, timed up and go test; UPDRS, Unified Parkinson's disease rating scale  * Shown as mean (standard deviation). ^#^Between-group difference in mean change from baseline with 95% CI | | | | | | | | | | | |  |

**Table S2 The association between clinical improvements and the switch rate of brain networks**

|  | Node code | Estimation^#^ | *P* values (Tai Chi-Control) | P values (Tai Chi-Brisk Walking) |
| --- | --- | --- | --- | --- |
| Berg balance scale | Frontal parietal control network, mainly left | -1.36 | 0.125 | 0.438 |
|  | Out of brain, noise | 1.51 | 0.063 | 0.077 |
|  | Ventral attention network | -1.21 | 0.185 | 0.736 |
|  | Dorsal attention network | 1.48 | 0.126 | 0.766 |
|  | Visual network* | 1.91 | 0.044 | 0.082 |
|  | Frontal parietal control network | -0.97 | 0.245 | 0.838 |
| UPDRS | Limbic network | 5.26 | 0.094 | 0.103 |
|  | Visual network | -2.73 | 0.242 | 0.377 |
|  | Ventral salient network* | -3.67 | 0.168 | 0.264 |
|  | Default mode network* | -5.69 | 0.023 | 0.044 |
| UPDRS – Part III | Limbic network | 3.02 | 0.229 | 0.926 |
|  | Visual network | -2.20 | 0.272 | 0.904 |
|  | Ventral attention network | -0.93 | 0.668 | 0.407 |
|  | Default mode network* | -6.26 | 0.006 | 0.015 |
|  | Frontoparietal network | 4.06 | 0.040 | 0.443 |
| Timed up and Go test | Ventral salient network | -0.09 | 0.893 | 0.838 |
|  | Frontal parietal control network | 0.57 | 0.217 | 0.936 |
|  | Default mode network | 0.81 | 0.326 | 0.133 |
|  | Ventral attention network | 0.83 | 0.176 | 0.290 |
|  | Dorsal attention network | -1.10 | 0.152 | 0.716 |
| UPDRS, Unified Parkinson's disease rating scale  * The association was also validated using Bayesian belief network  ^#^ Higher estimation value means more positive association between neural networks and the changes of rating scales | | | | |

**Table S3 Intergroup comparison of cytokines among Tai Chi group, Brisk Walking group and Control group**

| Measure | Tai Chi | Brisk Walking | Control | Between-group differences in longitudinal mean changes | | | | | |
| --- | --- | --- | --- | --- | --- | --- | --- | --- | --- |
|  |  |  |  |  | Tai Chi v.s. Control | P value | Tai Chi v.s. Brisk walking | P value | P value of 3 group comparison |
| **INTERLEUKINS** | | | | | | | | | |
| **IL-1β** |  |  |  |  |  |  |  |  |  |
| Baseline | 2.78 (0.85) | 1.70 (0.39) | 1.42 (2.13) |  |  |  |  |  |  |
| 6-month visit | 2.31 (1.07) | 1.97 (0.67) | 1.76 (1.31) | 6 month visit – baseline | 0.82 (0.09, 1.54) | 0.013 | 0.08 (-0.68, 0.84) | 0.053 | 0.005 |
| 12-month visit | 2.12 (1.71) | 1.96 (0.77) | 1.94 (1.22) | 1 year visit – baseline | 1.18 (0.18, 2.19) | 0.028 | 0.28 (-0.77, 1.32) | 0.096 | 0.017 |
| *P* value ^#^ | 0.051 |  | 0.047 |  |  |  |  |  |  |
| **IL-1RA** |  |  |  |  |  |  |  |  |  |
| Baseline | 424.00 (275.81) | 382.96 (63.25) | 190.65 (489.92) |  |  |  |  |  |  |
| 6-month visit | 242.51 (87.64) | 282.29 (100.54) | 275.38 (80.06) | 6 month visit – baseline | 266.23 (22.74, 509.72) | 0.123 | 185.4 (21.17, 349.63) | 0.872 | 0.110 |
| 12-month visit | 249.26 (220.40) | 327.14 (143.85) | 276.57 (77.72) | 1 year visit – baseline | 260.67 (19.8, 501.54) | 0.244 | 141.75 (-33.69, 317.19) | 0.721 | 0.260 |
| *P* value ^#^ | 0.072 |  | 0.026 |  |  |  |  |  |  |
| **IL-2** |  |  |  |  |  |  |  |  |  |
| Baseline | 6.39 (3.64) | 8.12 (2.30) | 5.11 (4.19) |  |  |  |  |  |  |
| 6-month visit | 6.58 (2.48) | 6.96 (3.11) | 6.05 (2.74) | 6 month visit – baseline | 0.74 (-1.2, 2.69) | 0.277 | 2.1 (-0.81, 5.02) | 0.810 | 0.230 |
| 12-month visit | 5.94 (3.70) | 6.29 (3.14) | 6.62 (1.98) | 1 year visit – baseline | 1.95 (-0.2, 4.11) | 0.575 | 3.34 (0.35, 6.33) | 0.778 | 0.537 |
| *P* value ^#^ | 0.135 |  | 0.044 |  |  |  |  |  |  |
| **IL-4** |  |  |  |  |  |  |  |  |  |
| Baseline | 8.88 (2.76) | 10.77 (2.39) | 6.56 (2.79) |  |  |  |  |  |  |
| 6-month visit | 8.68 (2.20) | 9.85 (2.36) | 8.29 (2.47) | 6 month visit – baseline | 1.93 (0.51, 3.36) | 0.060 | 2.65 (0.28, 5.02) | 0.102 | 0.100 |
| 12-month visit | 8.82 (3.13) | 8.65 (2.80) | 9.67 (2.38) | 1 year visit – baseline | 3.17 (1.53, 4.81) | 0.378 | 5.23 (2.7, 7.76) | 0.250 | 0.448 |
| *P* value ^#^ | 0.899 |  | 0.037 |  |  |  |  |  |  |
| **IL-5** |  |  |  |  |  |  |  |  |  |
| Baseline | 31.14 (32.08) | 38.38 (10.36) | 16.89 (20.06) |  |  |  |  |  |  |
| 6-month visit | 29.98 (49.68) | 41.71 (10.35) | 23.37 (17.06) | 6 month visit – baseline | 7.5 (0.19, 14.82) | 0.028 | 3.01 (-13.17, 19.19) | 0.117 | 0.309 |
| 12-month visit | 34.88 (20.04) | 32.88 (13.62) | 26.66 (55.91) | 1 year visit – baseline | 6.41 (-17.14, 29.95) | 0.248 | 15.97 (-3.28, 35.22) | 0.293 | 0.394 |
| P value ^#^ | 0.297 |  | 0.016 |  |  |  |  |  |  |
| **IL-6** |  |  |  |  |  |  |  |  |  |
| Baseline | 2.45 (1.51) | 2.74 (1.24) | 2.19 (1.62) |  |  |  |  |  |  |
| 6-month visit | 2.30 (1.06) | 1.86 (0.79) | 1.86 (1.04) | 6 month visit – baseline | -0.18 (-0.94, 0.57) | 0.312 | 0.55 (-0.56, 1.66) | 0.632 | 0.271 |
| 12-month visit | 2.42 (1.97) | 2.73 (1.29) | 1.96 (2.54) | 1 year visit – baseline | -0.28 (-1.8, 1.24) | 0.301 | -0.29 (-1.56, 0.97) | 0.876 | 0.311 |
| *P* value ^#^ | 0.415 |  | 0.062 |  |  |  |  |  |  |
| **IL-7** |  |  |  |  |  |  |  |  |  |
| Baseline | 21.61 (6.63) | 22.8 (13.25) | 24.05 (8.27) |  |  |  |  |  |  |
| 6-month visit | 22.56 (4.92) | 22.33 (9.23) | 29.30 (5.63) | 6 month visit – baseline | 4.3 (-2.32, 10.92) | 0.032 | 5.71 (-3.65, 15.07) | 0.800 | 0.026 |
| 12-month visit | 20.76 (9.89) | 21.54 (11.17) | 25.07 (4.77) | 1 year visit – baseline | 1.87 (-3.54, 7.27) | 0.016 | 2.27 (-6.17, 10.71) | 0.981 | 0.012 |
| *P* value ^#^ | 0.281 |  | 0.327 |  |  |  |  |  |  |
| **IL-9** |  |  |  |  |  |  |  |  |  |
| Baseline | 378.96 (123.74) | 413.81 (87.32) | 409.81 (126.00) |  |  |  |  |  |  |
| 6-month visit | 380.24 (170.78) | 422.14 (97.44) | 462.74 (83.06) | 6 month visit – baseline | 51.65 (-24.6, 127.9) | 0.037 | 44.59 (-53.52, 142.7) | 0.245 | 0.061 |
| 12-month visit | 354.22 (136.16) | 382.55 (96.33) | 398.28 (79.19) | 1 year visit – baseline | 13.21 (-66.72, 93.14) | 0.019 | 19.73 (-77.21, 116.67) | 0.280 | 0.039 |
| *P* value ^#^ | 0.079 |  | 0.319 |  |  |  |  |  |  |
| **IL-10** |  |  |  |  |  |  |  |  |  |
| Baseline | 9.90 (2.73) | 10.37 (5.95) | 9.19 (6.05) |  |  |  |  |  |  |
| 6-month visit | 9.86 (2.66) | 9.66 (5.84) | 8.70 (5.34) | 6 month visit – baseline | -0.45 (-3.95, 3.05) | 0.558 | 0.22 (-3.82, 4.26) | 0.698 | 0.504 |
| 12-month visit | 11.01 (3.52) | 8.27 (4.25) | 9.91 (15.09) | 1 year visit – baseline | -0.39 (-8.29, 7.51) | 0.639 | 2.81 (-1.78, 7.41) | 0.558 | 0.551 |
| *P* value ^#^ | 0.371 |  | 0.354 |  |  |  |  |  |  |
| **IL-12** |  |  |  |  |  |  |  |  |  |
| Baseline | 6.28 (2.28) | 3.64 (2.08) | 2.65 (10.69) |  |  |  |  |  |  |
| 6-month visit | 5.54 (2.94) | 4.03 (3.30) | 3.52 (6.91) | 6 month visit – baseline | 1.48 (-1.18, 4.14) | 0.218 | 0.5 (-2.01, 3.01) | 0.435 | 0.133 |
| 12-month visit | 6.26 (5.17) | 5.10 (2.63) | 3.88 (16.31) | 1 year visit – baseline | 1.06 (-2.65, 4.78) | 0.370 | -0.21 (-3.41, 2.98) | 0.713 | 0.290 |
| *P* value ^#^ | 0.621 |  | 0.031 |  |  |  |  |  |  |
| **IL-13** |  |  |  |  |  |  |  |  |  |
| Baseline | 20.75 (11.41) | 17.05 (2.88) | 12.35 (12.61) |  |  |  |  |  |  |
| 6-month visit | 21.62 (7.94) | 15.14 (3.53) | 13.01 (10.59) | 6 month visit – baseline | -0.21 (-4, 3.58) | 0.002 | 3.04 (-3.42, 9.5) | 0.019 | <0.001 |
| 12-month visit | 19.24 (7.91) | 14.52 (5.80) | 14.20 (11.50) | 1 year visit – baseline | 3.36 (-0.92, 7.64) | 0.005 | 4.63 (-3.09, 12.35) | 0.021 | 0.002 |
| *P* value ^#^ | 0.362 |  | 0.041 |  |  |  |  |  |  |
| **IL-15** |  |  |  |  |  |  |  |  |  |
| Baseline | 256.14 (85.88) | 234.74 (37.99) | 192.54 (140.58) |  |  |  |  |  |  |
| 6-month visit | 146.91 (60.49) | 195.15 (66.53) | 220.26 (110.92) | 6 month visit – baseline | 129.49 (16.09, 242.89) | 0.603 | 31.75 (-123.84, 187.33) | 0.502 | 0.530 |
| 12-month visit | 236.55 (127.70) | 291.66 (52.60) | 209.49 (260.91) | 1 year visit – baseline | 5.96 (-311.65, 323.57) | 0.855 | -29.96 (-188.16, 128.25) | 0.334 | 0.947 |
| *P* value ^#^ | 0.465 |  | 0.128 |  |  |  |  |  |  |
| **IL-17A** |  |  |  |  |  |  |  |  |  |
| Baseline | 13.06 (4.70) | 14.84 (4.54) | 8.96 (4.97) |  |  |  |  |  |  |
| 6-month visit | 12.97 (3.93) | 14.22 (6.32) | 13.46 (3.10) | 6 month visit – baseline | 4.59 (1.37, 7.81) | 0.147 | 5.12 (0.47, 9.77) | 0.519 | 0.147 |
| 12-month visit | 11.94 (7.39) | 13.94 (5.23) | 13.31 (2.28) | 1 year visit – baseline | 5.47 (2.26, 8.68) | 0.501 | 5.25 (-0.07, 10.57) | 0.181 | 0.603 |
| *P* value ^#^ | 0.132 |  | 0.087 |  |  |  |  |  |  |
| **COLONY STIMULATING FACTORS** | | | | | | | | | |
| **G-CSF** |  |  |  |  |  |  |  |  |  |
| Baseline | 250.08 (153.31) | 328.46 (53.73) | 199.07 (89.81) |  |  |  |  |  |  |
| 6-month visit | 252.50 (59.98) | 258.30 (189.45) | 302.81 (61.97) | 6 month visit – baseline | 101.32 (24.92, 177.72) | 0.926 | 173.9 (55.38, 292.43) | 0.407 | 0.961 |
| 12-month visit | 253.90 (177.99) | 276.29 (81.69) | 254.46 (68.39) | 1 year visit – baseline | 51.58 (6.14, 97.02) | 0.904 | 107.56 (18.17, 196.95) | 0.443 | 0.943 |
| *P* value ^#^ | 0.767 |  | 0.197 |  |  |  |  |  |  |
| **GM-CSF** |  |  |  |  |  |  |  |  |  |
| Baseline | 14.06 (3.33) | 6.77 (1.78) | 3.81 (16.82) |  |  |  |  |  |  |
| 6-month visit | 22.94 (3.71) | 5.89 (1.68) | 4.89 (16.35) | 6 month visit – baseline | -5.48 (-17.34, 6.38) | 0.005 | 1.71 (-2.85, 6.26) | 0.006 | <0.001 |
| 12-month visit | 31.37 (5.61) | 6.28 (2.79) | 5.80 (41.29) | 1 year visit – baseline | -8.24 (-36.22, 19.75) | 0.011 | 1.27 (-6.05, 8.6) | 0.014 | 0.002 |
| *P* value ^#^ | <0.001 |  | 0.082 |  |  |  |  |  |  |
| **CHEMOKINES** | | | | | | | | | |
| **IL-8** |  |  |  |  |  |  |  |  |  |
| Baseline | 9.36 (65.35) | 35.38 (3.38) | 7.26 (6.25) |  |  |  |  |  |  |
| 6-month visit | 10.35 (9.15) | 11.90 (19.28) | 14.93 (4.82) | 6 month visit – baseline | 6.69 (-0.67, 14.05) | 0.527 | 31.16 (-3.26, 65.58) | 0.181 | 0.838 |
| 12-month visit | 10.23 (61.82) | 31.09 (3.77) | 8.62 (8.47) | 1 year visit – baseline | 0.5 (-4.65, 5.66) | 0.862 | 5.66 (-25.66, 36.98) | 0.089 | 0.936 |
| *P* value ^#^ | 0.271 |  | 0.113 |  |  |  |  |  |  |
| **Eotaxin** |  |  |  |  |  |  |  |  |  |
| Baseline | 169.78 (61.49) | 221.75 (70.89) | 121.37 (54.66) |  |  |  |  |  |  |
| 6-month visit | 155.22 (49.02) | 197.75 (81.94) | 178.65 (43.51) | 6 month visit – baseline | 71.83 (30.49, 113.17) | 0.499 | 81.28 (19.71, 142.84) | 0.002 | 0.716 |
| 12-month visit | 168.89 (76.86) | 175.46 (91.38) | 216.78 (57.93) | 1 year visit – baseline | 96.29 (52.08, 140.5) | 0.536 | 141.7 (76.62, 206.77) | 0.023 | 0.438 |
| *P* value ^#^ | 0.449 |  | 0.014 |  |  |  |  |  |  |
| **IP-10** |  |  |  |  |  |  |  |  |  |
| Baseline | 1692.98 (1264.09) | 2116.27 (436.48) | 1296.59 (1094.90) |  |  |  |  |  |  |
| 6-month visit | 1538.01 (533.78) | 1516.24 (577.20) | 1483.19 (805.68) | 6 month visit – baseline | 341.57 (-253.53, 936.67) | 0.220 | 786.63 (23.14, 1550.13) | 0.534 | 0.298 |
| 12-month visit | 1424.93 (1055.63) | 1637.67 (423.54) | 1536.92 (509.35) | 1 year visit – baseline | 508.38 (-74.73, 1091.49) | 0.440 | 718.93 (-178.94, 1616.81) | 0.287 | 0.600 |
| *P* value ^#^ | 0.042 |  | 0.019 |  |  |  |  |  |  |
| **MCP-1** |  |  |  |  |  |  |  |  |  |
| Baseline | 40.79 (26.65) | 49.68 (11.33) | 28.48 (21.22) |  |  |  |  |  |  |
| 6-month visit | 36.86 (13.82) | 39.71 (9.30) | 31.88 (12.04) | 6 month visit – baseline | 7.33 (-3.52, 18.19) | 0.032 | 13.37 (-2.51, 29.26) | <0.001 | 0.126 |
| 12-month visit | 38.49 (23.12) | 44.56 (12.61) | 34.31 (14.01) | 1 year visit – baseline | 8.13 (-4.23, 20.5) | 0.060 | 10.95 (-9.22, 31.13) | 0.022 | 0.179 |
| *P* value ^#^ | 0.165 |  | 0.032 |  |  |  |  |  |  |
| **MIP-1α** |  |  |  |  |  |  |  |  |  |
| Baseline | 2.29 (4.28) | 4.80 (0.56) | 2.40 (0.63) |  |  |  |  |  |  |
| 6-month visit | 2.40 (1.44) | 2.92 (3.92) | 4.30 (0.54) | 6 month visit – baseline | 1.79 (0.37, 3.22) | 0.006 | 3.78 (1.08, 6.47) | 0.029 | 0.053 |
| 12-month visit | 2.47 (4.66) | 4.07 (0.81) | 2.41 (1.23) | 1 year visit – baseline | -0.17 (-0.82, 0.48) | 0.017 | 0.74 (-1.25, 2.73) | 0.036 | 0.188 |
| *P* value ^#^ | 0.077 |  | 0.463 |  |  |  |  |  |  |
| **PDGF-BB** |  |  |  |  |  |  |  |  |  |
| Baseline | 1167.49 (3243.85) | 2576.19 (1669.29) | 910.98 (826.06) |  |  |  |  |  |  |
| 6-month visit | 1349.14 (2484.49) | 2238.46 (2956.70) | 3249.65 (936.42) | 6 month visit – baseline | 2157.02 (1087.72, 3226.33) | 0.022 | 2676.4 (823.02, 4529.79) | 0.096 | 0.094 |
| 12-month visit | 1372.58 (2602.10) | 2328.19 (4202.82) | 2184.44 (1069.74) | 1 year visit – baseline | 1068.36 (-674.39, 2811.12) | 0.011 | 1521.46 (-934.71, 3977.63) | 0.088 | 0.079 |
| *P* value ^#^ | 0.085 |  | 0.406 |  |  |  |  |  |  |
| **MIP-1β** |  |  |  |  |  |  |  |  |  |
| Baseline | 105.60 (39.76) | 124.31 (24.12) | 119.99 (25.10) |  |  |  |  |  |  |
| 6-month visit | 113.33 (29.89) | 108.72 (39.05) | 142.99 (25.47) | 6 month visit – baseline | 15.26 (-5.52, 36.04) | 0.003 | 38.58 (8.35, 68.82) | 0.451 | 0.006 |
| 12-month visit | 108.41 (53.52) | 110.07 (25.40) | 112.80 (23.37) | 1 year visit – baseline | -10.01 (-28.59, 8.57) | 0.008 | 7.04 (-23.53, 37.61) | 0.591 | 0.032 |
| *P* value ^#^ | 0.105 |  | 0.417 |  |  |  |  |  |  |
| **RANTES** |  |  |  |  |  |  |  |  |  |
| Baseline | 3987.63 (3594.76) | 4841.67 (3152.83) | 4599.46 (3508.03) |  |  |  |  |  |  |
| 6-month visit | 3830.91 (2587.73) | 4175.96 (3919.65) | 5928.71 (1594.37) | 6 month visit – baseline | 1485.96 (-738.42, 3710.35) | 0.138 | 1994.95 (-381.76, 4371.67) | 0.958 | 0.178 |
| 12-month visit | 3418.11 (4097.64) | 4755.37 (2811.50) | 4327.75 (1711.64) | 1 year visit – baseline | 297.81 (-2092.79, 2688.42) | 0.062 | -185.42 (-3109.06, 2738.23) | 0.612 | 0.123 |
| *P* value ^#^ | 0.076 |  | 0.373 |  |  |  |  |  |  |
| **OTHER CYTOKINES** | | | | | | | | | |
| **IFN-γ** |  |  |  |  |  |  |  |  |  |
| Baseline | 14.18 (9.52) | 16.44 (6.15) | 13.23 (8.87) |  |  |  |  |  |  |
| 6-month visit | 13.91 (15.74) | 16.10 (4.50) | 12.05 (7.69) | 6 month visit – baseline | -0.91 (-5.18, 3.35) | 0.426 | -0.85 (-6.67, 4.97) | 0.304 | 0.736 |
| 12-month visit | 15.45 (10.72) | 17.20 (5.53) | 13.00 (21.83) | 1 year visit – baseline | -1.51 (-10.99, 7.98) | 0.594 | -1 (-6.21, 4.22) | 0.307 | 0.766 |
| *P* value ^#^ | 0.152 |  | 0.387 |  |  |  |  |  |  |
| **Basic FGF** |  |  |  |  |  |  |  |  |  |
| Baseline | 39.58 (8.16) | 41.08 (8.99) | 36.2 (10.18) |  |  |  |  |  |  |
| 6-month visit | 42.76 (8.85) | 42.49 (9.17) | 43.43 (7.01) | 6 month visit – baseline | 4.05 (-2.11, 10.2) | 0.591 | 5.82 (-2.24, 13.88) | 0.886 | 0.532 |
| 12-month visit | 37.41 (14.83) | 42.78 (9.57) | 39.83 (6.23) | 1 year visit – baseline | 5.81 (-0.6, 12.22) | 0.987 | 1.95 (-7.26, 11.15) | 0.41 | 0.934 |
| *P* value ^#^ | 0.316 |  | 0.318 |  |  |  |  |  |  |
| **TNF-α** |  |  |  |  |  |  |  |  |  |
| Baseline | 19.52 (7.16) | 23.95 (6.25) | 20.00 (6.14) |  |  |  |  |  |  |
| 6-month visit | 19.91 (4.34) | 20.74 (6.96) | 21.57 (4.22) | 6 month visit – baseline | 1.18 (-2.32, 4.67) | 0.600 | 4.78 (-1.29, 10.85) | 0.243 | 0.525 |
| 12-month visit | 20.27 (9.64) | 21.53 (7.36) | 20.85 (16.60) | 1 year visit – baseline | 0.1 (-9.1, 9.29) | 0.622 | 3.26 (-4.12, 10.65) | 0.330 | 0.545 |
| *P* value ^#^ | 0.114 |  | 0.214 |  |  |  |  |  |  |
| **VEGF** |  |  |  |  |  |  |  |  |  |
| Baseline | 288.01 (79.49) | 295.86 (55.88) | 208.69 (178.43) |  |  |  |  |  |  |
| 6-month visit | 290.20 (87.40) | 254.69 (63.52) | 238.45 (119.15) | 6 month visit – baseline | 27.57 (-25.73, 80.87) | 0.052 | 70.93 (-3.46, 145.33) | 0.261 | 0.024 |
| 12-month visit | 273.02 (154.78) | 316.68 (61.91) | 242.67 (115.79) | 1 year visit – baseline | 48.97 (-17.3, 115.24) | 0.083 | 13.17 (-85.58, 111.92) | 0.698 | 0.061 |
| *P* value ^#^ | 0.277 |  | 0.143 |  |  |  |  |  |  |
| # *P* value shows the self-change among longitudinal visits.  Between-group differences were analyzed using the (12 months or 6 months – baseline) data. | | | | | | | | | |

**Table S4** The association between changes of cytokines and rating scales

| Rating Scales | Cytokines * | *t* value ^#^ | *P* value ^#^ |
| --- | --- | --- | --- |
| Berg Balance Scale | IL1β | -2.377403 | 0.0214 |
|  | IL2 | -0.998827 | 0.3228 |
|  | IL5 | -1.389722 | 0.1709 |
|  | IL6 | -1.413487 | 0.1638 |
|  | IL8 | 1.764465 | 0.0839 |
|  | IL10 | -0.101376 | 0.9197 |
|  | IL12 | -0.994439 | 0.3249 |
|  | IL13 | 1.068653 | 0.2905 |
|  | GCSF | 0.475694 | 0.6364 |
|  | GMCSF | 1.186510 | 0.2411 |
|  | IFNγ | 2.223425 | 0.0308 |
|  | MIP1A | -1.150457 | 0.2555 |
|  | TNFα | 0.913004 | 0.3657 |
| UPDRS | IL1β | 0.702115 | 0.4840 |
|  | IL12 | 2.214635 | 0.0287 |
|  | IL13 | -1.628823 | 0.1060 |
|  | EOTAXIN | 3.200525 | 0.0018 |
|  | IFNγ | -2.649302 | 0.0092 |
| UPDRS – Part III | IL1β | 0.863909 | 0.3894 |
|  | IL12 | 3.320386 | 0.0012 |
|  | IL13 | -1.468674 | 0.1446 |
|  | EOTAXIN | 4.616695 | 0.0000 |
|  | IFNγ | -1.840140 | 0.0682 |
|  | TNFα | -2.714048 | 0.0076 |
| Timed Up and Go test | IL1β | 1.135559 | 0.2584 |
|  | IL2 | 2.282091 | 0.0243 |
|  | IL4 | -2.823321 | 0.0056 |
|  | IL8 | -0.670741 | 0.5037 |
|  | IL9 | 0.250724 | 0.8025 |
|  | IL13 | -1.520994 | 0.1309 |
|  | IL17α | 0.996626 | 0.3210 |
|  | BASICFGF | -0.974698 | 0.3317 |
|  | GCSF | 0.762200 | 0.4474 |
|  | MIP1α | -0.569598 | 0.5700 |
| * Cytokines were selected by penalized generalized estimating equations.  # Results were calculated by mixed effect regression using restricted maximum likelihood method. | | | |

**Table S5 Intergroup comparison of metabolites among Tai Chi group, Brisk Walking group and Control group**

| Measure | Tai Chi | Brisk Walking | Control | Between - group difference | | | | | | | |
| --- | --- | --- | --- | --- | --- | --- | --- | --- | --- | --- | --- |
|  |  |  |  |  | Tai Chi v.s. Control | *P* value | Brisk Walking v.s. Control | *P* value | Tai Chi v.s. Brisk walking | *P* value | *P* value of 3-group comparison |
| **Pyruvic acid** |  |  |  |  |  |  |  |  |  |  |  |
| Baseline | 2515.42 (2590.57) | 1337 (645.9) | 2511.59 (2213.34) |  |  |  |  |  |  |  |  |
| 6-month visit | 3730.94 (4464.27) | 1848.65 (3302.85) | 4071.65 (4592.21) | 6-month visit – baseline | -703.87 (-3570.97, 2163.23) | 0.687 | 1048.41 (-2290.91, 4387.73) | 0.02 | 344.54 (-2993.22, 3682.3) | 0.153 | 0.918 |
| 1-year visit | 5930.65 (6430.81) | 2924.53 (3483.23) | 4661.24 (5745.81) | 1-year visit – 6-month visit | -1123.83 (-5429.05, 3181.4) | 0.788 | -486.29 (-4733.21, 3760.62) | 0.058 | -1610.12 (-6497, 3276.76) | 0.157 | 0.594 |
|  |  |  |  | 1-year visit – baseline | -1827.7 (-5520.37, 1864.98) | 0.713 | 562.12 (-2739.88, 3864.11) | 0.166 | -1265.58 (-5231.01, 2699.86) | 0.214 | 0.553 |
| **L-Lactic acid** |  |  |  |  |  |  |  |  |  |  |  |
| Baseline | 1444622.65 (246096.02) | 1437455.35 (376928.69) | 1380850.71 (418709.04) |  |  |  |  |  |  |  |  |
| 6-month visit | 1398867.58 (386762.82) | 1516151.94 (316811.15) | 1568853.71 (276518.66) | 6-month visit – baseline | 124451.65 (-172055.36, 420958.67) | 0.468 | 109306.41 (-216672.59, 435285.41) | 0.836 | 233758.06 (-79333.12, 546849.24) | 0.903 | 0.528 |
| 1-year visit | 1521934.16 (356790.58) | 1477779.24 (445099.24) | 1423222.65 (366990.16) | 1-year visit – 6-month visit | -161439.29 (-461875.24, 138996.66) | 0.569 | -107258.35 (-479477.76, 264961.05) | 0.893 | -268697.64 (-567967.43, 30572.15) | 0.413 | 0.538 |
|  |  |  |  | 1-year visit – baseline | -36987.63 (-342388.29, 268413.02) | 0.558 | 2048.06 (-397629.19, 401725.31) | 0.293 | -34939.57 (-294401.49, 224522.34) | 0.585 | 0.602 |
| **Alpha-Hydroxyisobutyric acid** |  |  |  |  |  |  |  |  |  |  |  |
| Baseline | 1111.87 (346.91) | 1928.29 (1126.17) | 1800.12 (1123.7) |  |  |  |  |  |  |  |  |
| 6-month visit | 1502.32 (766.53) | 1543.35 (944.99) | 1622.94 (850.52) | 6-month visit – baseline | -775.39 (-1396.52, -154.26) | 0.019 | 207.76 (-633.79, 1049.32) | 0.822 | -567.63 (-1187.88, 52.63) | 0.068 | 0.037 |
| 1-year visit | 1409.06 (590.23) | 1812.94 (1112.87) | 1605.06 (1025.87) | 1-year visit – 6-month visit | 362.85 (-285.83, 1011.52) | 0.292 | -287.47 (-1175.61, 600.66) | 0.589 | 75.38 (-701.16, 851.92) | 0.355 | 0.313 |
|  |  |  |  | 1-year visit – baseline | -412.55 (-922.23, 97.13) | 0.01 | -79.71 (-1088.52, 929.11) | 0.608 | -492.25 (-1172.43, 187.93) | 0.021 | 0.026 |
| **Glycolic acid** |  |  |  |  |  |  |  |  |  |  |  |
| Baseline | 16710.13 (3575.74) | 13353.24 (2749.23) | 18186.65 (5505.05) |  |  |  |  |  |  |  |  |
| 6-month visit | 14065.32 (2978.46) | 11319.94 (2433.26) | 14986.82 (4147.31) | 6-month visit – baseline | 611.51 (-2114.77, 3337.8) | 0.224 | -1166.53 (-4867.94, 2534.89) | 0.001 | -555.02 (-3920.71, 2810.68) | 0 | 0.367 |
| 1-year visit | 15816.94 (5309.94) | 16074.82 (3955.42) | 16354.29 (12907.67) | 1-year visit – 6-month visit | 3003.27 (-358.09, 6364.63) | 0.615 | -3387.41 (-10419.78, 3644.95) | 0.292 | -384.14 (-6008.97, 5240.69) | 0.142 | 0.682 |
|  |  |  |  | 1-year visit – baseline | 3614.78 (734.63, 6494.93) | 0.523 | -4553.94 (-9846.68, 738.8) | 0.309 | -939.16 (-5114.75, 3236.43) | 0.261 | 0.54 |
| **L-Alanine** |  |  |  |  |  |  |  |  |  |  |  |
| Baseline | 241610 (90759.4) | 329084.65 (143055.14) | 317502.94 (136059.27) |  |  |  |  |  |  |  |  |
| 6-month visit | 332012.26 (119603.43) | 399407.94 (103465.3) | 310560.88 (133251.89) | 6-month visit – baseline | -20078.96 (-114198.62, 74040.7) | 0.435 | -77265.35 (-201951.06, 47420.35) | 0.152 | -97344.32 (-186051.11, -8637.53) | 0.058 | 0.334 |
| 1-year visit | 338747.68 (115827.27) | 356205.47 (125320.76) | 391546.82 (123227.09) | 1-year visit – 6-month visit | -49937.89 (-142197.07, 42321.29) | 0.675 | 124188.41 (5179.04, 243197.79) | 0.583 | 74250.52 (-24745.6, 173246.65) | 0.508 | 0.676 |
|  |  |  |  | 1-year visit – baseline | -70016.85 (-163668.28, 23634.57) | 0.024 | 46923.06 (-60531.93, 154378.05) | 0.746 | -23093.8 (-112532.8, 66345.21) | 0.199 | 0.029 |
| **Alpha-ketoisovaleric acid** |  |  |  |  |  |  |  |  |  |  |  |
| Baseline | 2201.94 (1070.55) | 1327.06 (563.39) | 1964.82 (1299.36) |  |  |  |  |  |  |  |  |
| 6-month visit | 2678.84 (1613.34) | 1778.71 (1205.32) | 2411 (1337.84) | 6-month visit – baseline | -25.26 (-1054.21, 1003.7) | 0.505 | -5.47 (-1201.69, 1190.75) | 0.023 | -30.73 (-1205.66, 1144.2) | 0.014 | 0.304 |
| 1-year visit | 2823.65 (1720.91) | 2707.59 (1285.75) | 2362.59 (1190.73) | 1-year visit – 6-month visit | 784.08 (-337.06, 1905.21) | 0.34 | -977.29 (-1954.6, 0.01) | 0.483 | -193.22 (-1415.98, 1029.54) | 0.334 | 0.26 |
|  |  |  |  | 1-year visit – baseline | 758.82 (-275.16, 1792.8) | 0.418 | -982.76 (-1982.23, 16.7) | 0.698 | -223.94 (-1301.34, 853.45) | 0.371 | 0.312 |
| **2-Hydroxybutyric acid** |  |  |  |  |  |  |  |  |  |  |  |
| Baseline | 32325.81 (13319.34) | 33667.35 (18091.84) | 37807.82 (15908.36) |  |  |  |  |  |  |  |  |
| 6-month visit | 36141.84 (13043.27) | 33136.53 (16591.09) | 37946.12 (20396.33) | 6-month visit – baseline | -4346.86 (-14230.35, 5536.63) | 0.321 | 669.12 (-14626.17, 15964.41) | 0.323 | -3677.74 (-14277.9, 6922.42) | 0.485 | 0.444 |
| 1-year visit | 33906.42 (13132.55) | 37884.76 (15017.09) | 35209.82 (10689.67) | 1-year visit – 6-month visit | 6983.65 (-3955.35, 17922.66) | 0.582 | -7484.53 (-22205.18, 7236.12) | 0.895 | -500.87 (-11331.84, 10330.09) | 0.786 | 0.644 |
|  |  |  |  | 1-year visit – baseline | 2636.8 (-7080.18, 12353.78) | 0.311 | -6815.41 (-17274.2, 3643.38) | 0.853 | -4178.61 (-14602.8, 6245.57) | 0.716 | 0.378 |
| **Oxalic acid** |  |  |  |  |  |  |  |  |  |  |  |
| Baseline | 92915.35 (18226.47) | 87845 (22690.68) | 105838.24 (47577.2) |  |  |  |  |  |  |  |  |
| 6-month visit | 88292.26 (46847.2) | 60163.24 (13636.03) | 106386.41 (42825.43) | 6-month visit – baseline | -23058.67 (-46134.27, 16.93) | 0.133 | 28229.94 (838.13, 55621.75) | 0.003 | 5171.27 (-22739.17, 33081.72) | 0.06 | 0.175 |
| 1-year visit | 90912.19 (23537.72) | 100604.65 (42291.38) | 67008.24 (47536.63) | 1-year visit – 6-month visit | 37821.48 (8542.47, 67100.49) | 0.759 | -79819.59 (-117430.5, -42208.68) | 0.46 | -41998.11 (-77325.55, -6670.67) | 0.325 | 0.637 |
|  |  |  |  | 1-year visit – baseline | 14762.81 (-7117.75, 36643.37) | 0.483 | -51589.65 (-94247.34, -8931.96) | 0.488 | -36826.84 (-65359.32, -8294.36) | 0.649 | 0.498 |
| **2-Hydroxy-3-methylbutyric acid** |  |  |  |  |  |  |  |  |  |  |  |
| Baseline | 9760.42 (6715.55) | 12231.94 (16311.57) | 13189.29 (6931.44) |  |  |  |  |  |  |  |  |
| 6-month visit | 8738 (3907.46) | 9039.71 (3424.92) | 13348.76 (9185.39) | 6-month visit – baseline | -2169.82 (-9323.93, 4984.3) | 0.009 | 3351.71 (-6589.08, 13292.5) | 0.473 | 1181.89 (-3696.26, 6060.04) | 0.43 | 0.018 |
| 1-year visit | 10292.16 (6023.46) | 11567.71 (7068.96) | 10454.65 (7058.49) | 1-year visit – 6-month visit | 973.84 (-2815.78, 4763.46) | 0.126 | -5422.12 (-11018.73, 174.5) | 0.478 | -4448.28 (-8483.1, -413.46) | 0.53 | 0.123 |
|  |  |  |  | 1-year visit – baseline | -1195.98 (-9034.54, 6642.59) | 0.183 | -2070.41 (-12230.24, 8089.42) | 0.699 | -3266.39 (-8235.27, 1702.49) | 0.298 | 0.22 |
| **L-Alpha-aminobutyric acid** |  |  |  |  |  |  |  |  |  |  |  |
| Baseline | 16218.23 (7463.18) | 18176.12 (7900.15) | 18384.76 (6263.44) |  |  |  |  |  |  |  |  |
| 6-month visit | 21329.16 (6325.42) | 21458 (7406.75) | 21117.94 (10941.92) | 6-month visit – baseline | -1829.05 (-7722.71, 4064.6) | 0.66 | -548.71 (-7544.84, 6447.42) | 0.93 | -2377.76 (-8281.69, 3526.17) | 0.826 | 0.647 |
| 1-year visit | 20233.77 (7444.17) | 20851 (7966.48) | 20466.12 (7852.79) | 1-year visit – 6-month visit | 488.39 (-5150, 6126.77) | 0.914 | -44.82 (-8553.23, 8463.58) | 0.96 | 443.56 (-5945.4, 6832.52) | 0.814 | 0.896 |
|  |  |  |  | 1-year visit – baseline | -1340.67 (-6890.6, 4209.27) | 0.598 | -593.53 (-7155.98, 5968.92) | 0.872 | -1934.2 (-7197.72, 3329.33) | 0.873 | 0.602 |
| **Malonic acid** |  |  |  |  |  |  |  |  |  |  |  |
| Baseline | 2654.87 (623.44) | 2304.71 (685.34) | 2599.53 (895.52) |  |  |  |  |  |  |  |  |
| 6-month visit | 2337.58 (807.4) | 2273 (987.66) | 2489.76 (791.66) | 6-month visit – baseline | 285.58 (-384.08, 955.25) | 0.635 | -78.06 (-952.3, 796.18) | 0.431 | 207.53 (-447.41, 862.47) | 0.602 | 0.622 |
| 1-year visit | 2850.9 (933.05) | 2307 (786.03) | 2330.41 (705.15) | 1-year visit – 6-month visit | -479.32 (-1221.68, 263.04) | 0.333 | -193.35 (-1092.84, 706.14) | 0.563 | -672.68 (-1348.65, 3.3) | 0.157 | 0.263 |
|  |  |  |  | 1-year visit – baseline | -193.74 (-876.74, 489.26) | 0.111 | -271.41 (-1031.49, 488.66) | 0.332 | -465.15 (-1201.86, 271.56) | 0.036 | 0.072 |
| **Glyceraldehyde** |  |  |  |  |  |  |  |  |  |  |  |
| Baseline | 3602.29 (1522.32) | 5435.47 (1534.21) | 4885.12 (1430.9) |  |  |  |  |  |  |  |  |
| 6-month visit | 5303.84 (1521.26) | 6536.47 (1856.47) | 5112.12 (2187.38) | 6-month visit – baseline | -600.55 (-1999.65, 798.55) | 0.206 | -874 (-2790.36, 1042.36) | 0.05 | -1474.55 (-2833.75, -115.34) | 0.001 | 0.121 |
| 1-year visit | 5370.16 (2090.62) | 5333.06 (1836.37) | 6285.88 (1115.46) | 1-year visit – 6-month visit | -1269.73 (-3002.16, 462.7) | 0.381 | 2377.18 (446.74, 4307.61) | 0.751 | 1107.44 (-541.88, 2756.77) | 0.492 | 0.408 |
|  |  |  |  | 1-year visit – baseline | -1870.28 (-3247.33, -493.23) | 0.012 | 1503.18 (8.62, 2997.73) | 0.399 | -367.11 (-1667.5, 933.29) | 0.174 | 0.009 |
| **L-Valine** |  |  |  |  |  |  |  |  |  |  |  |
| Baseline | 248378.61 (80016.94) | 271612.35 (91675.99) | 308793.53 (89010.97) |  |  |  |  |  |  |  |  |
| 6-month visit | 303164.45 (86002.13) | 312207.65 (88919.44) | 288486.88 (119353.66) | 6-month visit – baseline | -14190.54 (-75723.22, 47342.13) | 0.395 | -60901.94 (-142425.19, 20621.3) | 0.763 | -75092.49 (-142074.73, -8110.25) | 0.929 | 0.409 |
| 1-year visit | 289671.19 (87498.84) | 294032.41 (75796.37) | 296854.94 (113621.36) | 1-year visit – 6-month visit | -4681.98 (-71081.58, 61717.63) | 0.785 | 26543.29 (-79577.71, 132664.3) | 0.733 | 21861.32 (-66830.55, 110553.19) | 0.607 | 0.713 |
|  |  |  |  | 1-year visit – baseline | -18872.52 (-83199.01, 45453.96) | 0.155 | -34358.65 (-117368.92, 48651.62) | 0.349 | -53231.17 (-122722.58, 16260.24) | 0.887 | 0.156 |
| **Hydroxyurea** |  |  |  |  |  |  |  |  |  |  |  |
| Baseline | 277.68 (61.04) | 327.18 (118.71) | 410.94 (189.35) |  |  |  |  |  |  |  |  |
| 6-month visit | 295.87 (113.83) | 284.59 (173.02) | 413.82 (91.91) | 6-month visit – baseline | -60.78 (-151.7, 30.13) | 0 | 45.47 (-99.06, 190) | 0.012 | -15.31 (-116.57, 85.94) | 0.737 | 0 |
| 1-year visit | 338.68 (109.86) | 349.06 (153.7) | 257.76 (101.74) | 1-year visit – 6-month visit | 21.66 (-80.95, 124.28) | 0.364 | -220.53 (-333.36, -107.7) | 0.57 | -198.87 (-294.96, -102.77) | 0.832 | 0.553 |
|  |  |  |  | 1-year visit – baseline | -39.12 (-133.48, 55.24) | 0.278 | -175.06 (-298.67, -51.45) | 0.982 | -214.18 (-304.43, -123.92) | 0.597 | 0.34 |
| **Urea** |  |  |  |  |  |  |  |  |  |  |  |
| Baseline | 97994.35 (47926) | 139554 (70222.39) | 170530 (44560.49) |  |  |  |  |  |  |  |  |
| 6-month visit | 169912.9 (61695.34) | 197758.59 (65466.52) | 138846.82 (72224.59) | 6-month visit – baseline | -13713.96 (-62191.37, 34763.45) | 0.236 | -89887.76 (-148597.1, -31178.43) | 0.499 | -103601.72 (-144004.25, -63199.2) | 0.121 | 0.179 |
| 1-year visit | 161937.06 (45982.51) | 130049.41 (50667.21) | 186244.71 (65911.47) | 1-year visit – 6-month visit | -59733.34 (-106852.93, -12613.75) | 0.64 | 115107.06 (45130.79, 185083.33) | 0.955 | 55373.72 (621.71, 110125.73) | 0.739 | 0.663 |
|  |  |  |  | 1-year visit – baseline | -73447.3 (-113940.44, -32954.15) | 0 | 25219.29 (-31780.07, 82218.65) | 0.003 | -48228 (-87751.44, -8704.57) | 0.967 | 0.001 |
| **Caprylic acid** |  |  |  |  |  |  |  |  |  |  |  |
| Baseline | 335.48 (389.75) | 438.12 (513.84) | 378.06 (120.25) |  |  |  |  |  |  |  |  |
| 6-month visit | 275.58 (99.77) | 309.88 (144.97) | 308.88 (184.88) | 6-month visit – baseline | -68.33 (-320.91, 184.24) | 0.434 | 59.06 (-196.62, 314.74) | 0.752 | -9.27 (-215.68, 197.14) | 0.623 | 0.585 |
| 1-year visit | 401.06 (169.73) | 426.53 (226.35) | 304.53 (210.65) | 1-year visit – 6-month visit | -8.84 (-136.44, 118.76) | 0.61 | -121 (-298.35, 56.35) | 0.157 | -129.84 (-266.06, 6.39) | 0.543 | 0.632 |
|  |  |  |  | 1-year visit – baseline | -77.17 (-359.12, 204.78) | 0.938 | -61.94 (-338.51, 214.63) | 0.302 | -139.11 (-360.86, 82.64) | 0.466 | 0.864 |
| **Ethanolamine** |  |  |  |  |  |  |  |  |  |  |  |
| Baseline | 4984.9 (1042.62) | 7803.06 (4321.77) | 6596.06 (1347.07) |  |  |  |  |  |  |  |  |
| 6-month visit | 5673.35 (1098.37) | 6246.71 (2272.78) | 6927.41 (2353.75) | 6-month visit – baseline | -2244.8 (-3843, -646.6) | 0 | 1887.71 (-357.74, 4133.15) | 0.781 | -357.1 (-1527.61, 813.41) | 0.022 | 0.007 |
| 1-year visit | 5686.16 (1402.88) | 7863.41 (4358.82) | 6388.76 (1446.96) | 1-year visit – 6-month visit | 1603.9 (290.46, 2917.34) | 0.001 | -2155.35 (-4264.72, -45.98) | 0.723 | -551.45 (-1896.83, 793.93) | 0.086 | 0.067 |
|  |  |  |  | 1-year visit – baseline | -640.91 (-2047.74, 765.93) | 0 | -267.65 (-2177.02, 1641.73) | 0.241 | -908.55 (-2001.39, 184.28) | 0.007 | 0.067 |
| **Glycerol** |  |  |  |  |  |  |  |  |  |  |  |
| Baseline | 68090.84 (42883.03) | 75318.71 (36216.38) | 82650.53 (54512.66) |  |  |  |  |  |  |  |  |
| 6-month visit | 76258.84 (40745.83) | 74495.82 (36728.81) | 85424.71 (47086.33) | 6-month visit – baseline | -8990.88 (-45019.87, 27038.1) | 0.127 | 3597.06 (-38325.68, 45519.8) | 0.6 | -5393.82 (-44839.94, 34052.29) | 0.523 | 0.124 |
| 1-year visit | 68340.13 (46292.45) | 72954.47 (40284.89) | 71425.24 (33322.96) | 1-year visit – 6-month visit | 6377.36 (-27277.52, 40032.24) | 0.391 | -12458.12 (-48412.08, 23495.84) | 0.727 | -6080.76 (-36378.35, 24216.83) | 0.85 | 0.451 |
|  |  |  |  | 1-year visit – baseline | -2613.53 (-37394.88, 32167.83) | 0.31 | -8861.06 (-51002.83, 33280.71) | 0.744 | -11474.58 (-49546.41, 26597.24) | 0.753 | 0.347 |
| **DL O Phosphoserine** |  |  |  |  |  |  |  |  |  |  |  |
| Baseline | 464511.58 (233867.02) | 557577.82 (201898.9) | 597713.82 (214427.41) |  |  |  |  |  |  |  |  |
| 6-month visit | 606928.97 (300392.76) | 599317.24 (286525.67) | 537582.59 (291514.71) | 6-month visit – baseline | -100677.98 (-328773.57, 127417.62) | 0.845 | -101870.65 (-329540.81, 125799.52) | 0.982 | -202548.62 (-437045.44, 31948.19) | 0.775 | 0.766 |
| 1-year visit | 637032.26 (288330.52) | 556207.82 (303300.14) | 529708.18 (266863.53) | 1-year visit – 6-month visit | -73212.7 (-302865.74, 156440.34) | 0.222 | 35235 (-230451.75, 300921.75) | 0.67 | -37977.7 (-259971.79, 184016.39) | 0.36 | 0.159 |
|  |  |  |  | 1-year visit – baseline | -173890.68 (-410525.94, 62744.59) | 0.911 | -66635.65 (-331935.19, 198663.89) | 0.945 | -240526.32 (-468134.38, -12918.27) | 0.701 | 0.955 |
| **Phosphoric acid** |  |  |  |  |  |  |  |  |  |  |  |
| Baseline | 604982.23 (190542.53) | 639964.59 (127183.26) | 652658.65 (168359.91) |  |  |  |  |  |  |  |  |
| 6-month visit | 674095.29 (243901.23) | 640958.53 (220646.62) | 632153.82 (188467.6) | 6-month visit – baseline | -68119.12 (-225770.22, 89531.97) | 0.797 | -21498.76 (-180282.58, 137285.05) | 0.872 | -89617.89 (-246613.19, 67377.41) | 0.842 | 0.865 |
| 1-year visit | 691646.87 (275655.85) | 661341.65 (252224.04) | 661524.29 (191108.44) | 1-year visit – 6-month visit | 2831.54 (-191044.06, 196707.13) | 0.505 | 8987.35 (-214764, 232738.71) | 0.907 | 11818.89 (-164295.07, 187932.85) | 0.497 | 0.414 |
|  |  |  |  | 1-year visit – baseline | -65287.59 (-271485.91, 140910.74) | 0.968 | -12511.41 (-197953.46, 172930.64) | 0.684 | -77799 (-281011.66, 125413.67) | 0.771 | 0.925 |
| **L-Alloisoleucine** |  |  |  |  |  |  |  |  |  |  |  |
| Baseline | 1239.68 (583.85) | 1366.29 (586.75) | 1588.59 (526.6) |  |  |  |  |  |  |  |  |
| 6-month visit | 1535.29 (667.23) | 1485.06 (444.83) | 1642.88 (757.65) | 6-month visit – baseline | -176.85 (-626.11, 272.41) | 0.134 | -64.47 (-599.76, 470.82) | 0.386 | -241.32 (-717.83, 235.19) | 0.995 | 0.123 |
| 1-year visit | 1419.48 (655.02) | 1431.82 (481.74) | 1603.24 (653.66) | 1-year visit – 6-month visit | 62.57 (-375.87, 501.01) | 0.352 | 13.59 (-438.03, 465.2) | 0.496 | 76.16 (-408.32, 560.64) | 0.912 | 0.354 |
|  |  |  |  | 1-year visit – baseline | -114.28 (-568.23, 339.68) | 0.079 | -50.88 (-547.82, 446.05) | 0.463 | -165.16 (-605.4, 275.08) | 0.805 | 0.057 |
| **L-Isoleucine** |  |  |  |  |  |  |  |  |  |  |  |
| Baseline | 67210.55 (30702.89) | 69601.94 (20333.3) | 86533.71 (41164.81) |  |  |  |  |  |  |  |  |
| 6-month visit | 75912.26 (27788.25) | 76077.71 (24644.53) | 72036.29 (38074.66) | 6-month visit – baseline | -2225.94 (-22183.93, 17732.04) | 0.456 | -20973.18 (-51429.92, 9483.56) | 0.347 | -23199.12 (-49180.7, 2782.45) | 0.794 | 0.438 |
| 1-year visit | 73500.16 (25682.05) | 77529.71 (22133.11) | 80852.71 (44691.65) | 1-year visit – 6-month visit | 3864.1 (-17142.07, 24870.27) | 0.924 | 7364.41 (-29627.46, 44356.28) | 0.998 | 11228.51 (-17731.02, 40188.04) | 0.686 | 0.952 |
|  |  |  |  | 1-year visit – baseline | 1638.15 (-19314.14, 22590.45) | 0.146 | -13608.76 (-44102.31, 16884.78) | 0.202 | -11970.61 (-39458.36, 15517.14) | 0.854 | 0.115 |
| **L-Proline** |  |  |  |  |  |  |  |  |  |  |  |
| Baseline | 273577 (130820.68) | 265532.88 (119270.77) | 295204.35 (142872.19) |  |  |  |  |  |  |  |  |
| 6-month visit | 346450.32 (165134.26) | 318976.94 (79515.22) | 270806 (119176.37) | 6-month visit – baseline | -19429.26 (-127120.04, 88261.51) | 0.352 | -77842.41 (-193918.45, 38233.62) | 0.948 | -97271.68 (-208573.25, 14029.9) | 0.428 | 0.269 |
| 1-year visit | 351707.23 (145101.81) | 377408.24 (171442.79) | 296039.88 (124550.95) | 1-year visit – 6-month visit | 53174.39 (-54110.46, 160459.24) | 0.057 | -33197.41 (-161096.63, 94701.81) | 0.08 | 19976.98 (-84513.72, 124467.67) | 0.885 | 0.05 |
|  |  |  |  | 1-year visit – baseline | 33745.13 (-66670.89, 134161.14) | 0.609 | -111039.82 (-236458.9, 14379.25) | 0.554 | -77294.7 (-180360.13, 25770.74) | 0.72 | 0.66 |
| **Glycine** |  |  |  |  |  |  |  |  |  |  |  |
| Baseline | 223604.42 (74704.85) | 227894.18 (69299.54) | 272635.18 (107277.58) |  |  |  |  |  |  |  |  |
| 6-month visit | 249852.97 (95366.05) | 197904.41 (44002.03) | 288623.94 (84891.58) | 6-month visit – baseline | -56238.31 (-106925.82, -5550.81) | 0.036 | 45978.53 (-41016.94, 132974) | 0.001 | -10259.78 (-79374.73, 58855.16) | 0.619 | 0.042 |
| 1-year visit | 221856.48 (63899.69) | 299155.88 (109534.27) | 251073.35 (85399.54) | 1-year visit – 6-month visit | 129247.95 (74920.2, 183575.71) | 0.065 | -138802.06 (-209416.81, -68187.31) | 0.31 | -9554.1 (-68883.3, 49775.09) | 0.291 | 0.08 |
|  |  |  |  | 1-year visit – baseline | 73009.64 (24542.2, 121477.08) | 0.063 | -92823.53 (-165950.64, -19696.42) | 0.787 | -19813.89 (-69412.34, 29784.57) | 0.031 | 0.064 |
| **Succinic acid** |  |  |  |  |  |  |  |  |  |  |  |
| Baseline | 38388.9 (8287.64) | 47042.29 (13553.04) | 41721.35 (20614.37) |  |  |  |  |  |  |  |  |
| 6-month visit | 29313.16 (5804.39) | 30258.06 (9205.03) | 36533.94 (8889.83) | 6-month visit – baseline | -7708.49 (-14760.2, -656.79) | 0.054 | 11596.82 (1185.69, 22007.96) | 0.866 | 3888.33 (-3770.07, 11546.73) | 0.073 | 0.04 |
| 1-year visit | 41750.58 (12907.26) | 41583.76 (8579.82) | 30883.47 (12004.6) | 1-year visit – 6-month visit | -1111.71 (-9063.14, 6839.71) | 0.63 | -16976.18 (-24305.55, -9646.8) | 0.446 | -18087.89 (-24892.02, -11283.76) | 0.809 | 0.54 |
|  |  |  |  | 1-year visit – baseline | -8820.21 (-17670.29, 29.88) | 0.367 | -5379.35 (-16538.14, 5779.43) | 0.029 | -14199.56 (-23594.59, -4804.53) | 0.086 | 0.453 |
| **Glyceric acid** |  |  |  |  |  |  |  |  |  |  |  |
| Baseline | 16774.16 (7937.39) | 19686.29 (9511.46) | 16576.88 (11099.06) |  |  |  |  |  |  |  |  |
| 6-month visit | 15464.94 (6515.18) | 17172.06 (7261.56) | 15458.59 (8814.3) | 6-month visit – baseline | -1205.01 (-6324.31, 3914.29) | 0.979 | 1395.94 (-7428.14, 10220.03) | 0.367 | 190.93 (-6473.34, 6855.21) | 0.537 | 0.979 |
| 1-year visit | 13221.65 (7201.89) | 18714.35 (7825.09) | 13075.76 (5753.9) | 1-year visit – 6-month visit | 3785.58 (-1675.44, 9246.61) | 0.892 | -3925.12 (-10399.22, 2548.98) | 0.078 | -139.53 (-6299.91, 6020.84) | 0.165 | 0.871 |
|  |  |  |  | 1-year visit – baseline | 2580.57 (-3666.02, 8827.17) | 0.911 | -2529.18 (-9780.27, 4721.91) | 0.143 | 51.4 (-6873.76, 6976.56) | 0.156 | 0.982 |
| **Picolinic acid** |  |  |  |  |  |  |  |  |  |  |  |
| Baseline | 3743.48 (2823.27) | 2395.18 (623.31) | 2843.47 (800.06) |  |  |  |  |  |  |  |  |
| 6-month visit | 2060.19 (853.54) | 1554.76 (573.84) | 2641.18 (1013.09) | 6-month visit – baseline | 842.88 (-685.28, 2371.04) | 0.755 | 638.12 (-174.8, 1451.04) | 0.002 | 1481 (-92.11, 3054.1) | 0.051 | 0.561 |
| 1-year visit | 3872.16 (4212.08) | 2525.76 (1041.4) | 1859.35 (857.76) | 1-year visit – 6-month visit | -840.97 (-3169.84, 1487.91) | 0.322 | -1752.82 (-2654.93, -850.72) | 0.473 | -2593.79 (-4914.39, -273.19) | 0.429 | 0.197 |
|  |  |  |  | 1-year visit – baseline | 1.91 (-2661.76, 2665.58) | 0.05 | -1114.71 (-2021.45, -207.96) | 0.517 | -1112.8 (-3767.86, 1542.27) | 0.196 | 0.016 |
| **Citraconic acid** |  |  |  |  |  |  |  |  |  |  |  |
| Baseline | 6259.32 (3915.79) | 5864.41 (2293.51) | 6728.71 (3345.17) |  |  |  |  |  |  |  |  |
| 6-month visit | 5541.52 (2058.45) | 4481.18 (2464.98) | 5586.06 (2800.48) | 6-month visit – baseline | -665.43 (-3108.68, 1777.82) | 0.785 | 240.59 (-2504.4, 2985.58) | 0.254 | -424.84 (-2924.33, 2074.65) | 0.051 | 0.923 |
| 1-year visit | 6855 (2862.26) | 8087.88 (2139.94) | 5265.24 (2506) | 1-year visit – 6-month visit | 2293.22 (439.78, 4146.67) | 0.198 | -3927.53 (-6223.66, -1631.4) | 0.178 | -1634.31 (-3761.49, 492.88) | 0.693 | 0.199 |
|  |  |  |  | 1-year visit – baseline | 1627.79 (-993.52, 4249.11) | 0.407 | -3686.94 (-5922.87, -1451.01) | 0.227 | -2059.15 (-4755.29, 637) | 0.575 | 0.334 |
| **Fumaric acid** |  |  |  |  |  |  |  |  |  |  |  |
| Baseline | 2065.71 (279.13) | 2405.35 (519.51) | 2380.94 (397.75) |  |  |  |  |  |  |  |  |
| 6-month visit | 1696.52 (486.1) | 1445.71 (386.15) | 2259.82 (619.84) | 6-month visit – baseline | -590.45 (-919.71, -261.2) | 0 | 838.53 (367.19, 1309.87) | 0.013 | 248.08 (-104.08, 600.24) | 0.747 | 0.001 |
| 1-year visit | 2368.77 (532.39) | 2369.59 (445.48) | 1494 (435.54) | 1-year visit – 6-month visit | 251.62 (-146.14, 649.39) | 0.277 | -1689.71 (-2125.92, -1253.5) | 0.954 | -1438.08 (-1840.88, -1035.28) | 0.515 | 0.216 |
|  |  |  |  | 1-year visit – baseline | -338.83 (-675.21, -2.45) | 0.026 | -851.18 (-1157.86, -544.49) | 0.004 | -1190.01 (-1555.49, -824.53) | 0.13 | 0.031 |
| **Pyrrole-2-carboxylic acid** |  |  |  |  |  |  |  |  |  |  |  |
| Baseline | 708.45 (277.25) | 546.71 (133.87) | 665.59 (164.62) |  |  |  |  |  |  |  |  |
| 6-month visit | 414.03 (193.57) | 329.06 (132.65) | 607.41 (274.1) | 6-month visit – baseline | 76.77 (-89.52, 243.06) | 0.166 | 159.47 (-11.8, 330.74) | 0.001 | 236.24 (55.19, 417.29) | 0.077 | 0.238 |
| 1-year visit | 720.42 (470.21) | 612.71 (142.33) | 431.29 (380.36) | 1-year visit – 6-month visit | -22.74 (-293.72, 248.24) | 0.673 | -459.76 (-721.97, -197.56) | 0.498 | -482.5 (-797.03, -167.98) | 0.544 | 0.547 |
|  |  |  |  | 1-year visit – baseline | 54.03 (-248.36, 356.42) | 0.065 | -300.29 (-533.69, -66.9) | 0.488 | -246.26 (-571.24, 78.72) | 0.226 | 0.026 |
| **L-Serine** |  |  |  |  |  |  |  |  |  |  |  |
| Baseline | 63795.61 (18807.58) | 90748.29 (29791.63) | 85480.88 (30108.03) |  |  |  |  |  |  |  |  |
| 6-month visit | 76088.29 (20010.01) | 76121.59 (17622.23) | 87412 (26495.81) | 6-month visit – baseline | -26919.38 (-44513.2, -9325.57) | 0.003 | 16557.82 (-9435.19, 42550.84) | 0.653 | -10361.56 (-30186.75, 9463.63) | 0.024 | 0.002 |
| 1-year visit | 79621.45 (24395.84) | 96427.76 (27544.72) | 80484.06 (25296.23) | 1-year visit – 6-month visit | 16773.02 (-1837.84, 35383.87) | 0.214 | -27234.12 (-53324.12, -1144.12) | 0.94 | -10461.1 (-31955.2, 11033) | 0.193 | 0.226 |
|  |  |  |  | 1-year visit – baseline | -10146.37 (-28784.6, 8491.86) | 0.062 | -10676.29 (-31251.59, 9899) | 0.359 | -20822.66 (-38523.39, -3121.93) | 0.002 | 0.063 |
| **Pipecolic acid** |  |  |  |  |  |  |  |  |  |  |  |
| Baseline | 1058.32 (678.98) | 1300.94 (1126.21) | 2278.35 (2454.83) |  |  |  |  |  |  |  |  |
| 6-month visit | 1611.55 (830.39) | 1741.71 (710.21) | 1717.53 (1297.75) | 6-month visit – baseline | -112.46 (-770.12, 545.2) | 0.032 | -1001.59 (-2571.49, 568.32) | 0.428 | -1114.05 (-2172.19, -55.91) | 0.785 | 0.019 |
| 1-year visit | 1703.45 (720.99) | 1986.29 (1991.76) | 1993 (2284.84) | 1-year visit – 6-month visit | 152.69 (-694.62, 999.99) | 0.657 | 30.88 (-1571.16, 1632.92) | 0.864 | 183.57 (-890.27, 1257.4) | 0.856 | 0.659 |
|  |  |  |  | 1-year visit – baseline | 40.22 (-808.03, 888.47) | 0.025 | -970.71 (-2992.18, 1050.77) | 0.362 | -930.48 (-2279.32, 418.35) | 0.742 | 0.032 |
| **L-Threonine** |  |  |  |  |  |  |  |  |  |  |  |
| Baseline | 60145.52 (14985.59) | 69151.76 (24331.94) | 65216.88 (24154.44) |  |  |  |  |  |  |  |  |
| 6-month visit | 64914.06 (13829.53) | 66609.53 (25318.19) | 66508.76 (20157.58) | 6-month visit – baseline | -7310.78 (-22984.8, 8363.24) | 0.573 | 3834.12 (-17451.59, 25119.83) | 0.725 | -3476.67 (-15824.85, 8871.52) | 0.303 | 0.503 |
| 1-year visit | 64365.26 (14497.21) | 77399.94 (17182.99) | 66852.94 (22956.42) | 1-year visit – 6-month visit | 11339.22 (-3826.9, 26505.33) | 0.646 | -10446.24 (-33954.4, 13061.93) | 0.413 | 892.98 (-14440.99, 16226.95) | 0.111 | 0.579 |
|  |  |  |  | 1-year visit – baseline | 4028.43 (-10729.3, 18786.16) | 0.485 | -6612.12 (-24736.85, 11512.62) | 0.367 | -2583.68 (-16672.43, 11505.07) | 0.024 | 0.379 |
| **Glutaric acid** |  |  |  |  |  |  |  |  |  |  |  |
| Baseline | 977.32 (525.11) | 1545.94 (991.65) | 1070.76 (495.44) |  |  |  |  |  |  |  |  |
| 6-month visit | 895.42 (510.46) | 1108.29 (628.49) | 1193.82 (667.94) | 6-month visit – baseline | -355.74 (-785.6, 74.11) | 0.103 | 560.71 (95.06, 1026.35) | 0.317 | 204.96 (-226.3, 636.23) | 0.071 | 0.174 |
| 1-year visit | 1149.87 (779.72) | 1384.53 (789.61) | 1070.71 (493.65) | 1-year visit – 6-month visit | 21.78 (-410.44, 454.01) | 0.357 | -399.35 (-900.95, 102.24) | 0.59 | -377.57 (-867.33, 112.19) | 0.281 | 0.417 |
|  |  |  |  | 1-year visit – baseline | -333.96 (-820.23, 152.31) | 0.682 | 161.35 (-311.15, 633.86) | 0.088 | -172.61 (-689.2, 343.99) | 0.047 | 0.704 |
| **Methylcysteine** |  |  |  |  |  |  |  |  |  |  |  |
| Baseline | 1746.74 (924.88) | 1714.29 (1405.53) | 2876.47 (1911.44) |  |  |  |  |  |  |  |  |
| 6-month visit | 2574.48 (1077.15) | 1575.59 (900.68) | 1695.65 (643.85) | 6-month visit – baseline | -966.45 (-1904.45, -28.44) | 0.476 | -1042.12 (-2326.32, 242.09) | 0.141 | -2008.57 (-2931.79, -1085.34) | 0.09 | 0.604 |
| 1-year visit | 2168.03 (1415.34) | 1754.94 (1157.67) | 2117.82 (1820.46) | 1-year visit – 6-month visit | 585.8 (-253.71, 1425.32) | 0.126 | 242.82 (-835.6, 1321.25) | 0.293 | 828.63 (-178.74, 1835.99) | 0.061 | 0.085 |
|  |  |  |  | 1-year visit – baseline | -380.64 (-1444.81, 683.52) | 0.1 | -799.29 (-2374.03, 775.44) | 0.064 | -1179.94 (-2312.59, -47.28) | 0.716 | 0.1 |
| **Hydrocinnamic acid** |  |  |  |  |  |  |  |  |  |  |  |
| Baseline | 974.58 (1342.28) | 671.53 (510.98) | 1885.65 (2438.76) |  |  |  |  |  |  |  |  |
| 6-month visit | 866.77 (850.38) | 586.41 (293.36) | 862.18 (1270.4) | 6-month visit – baseline | 22.69 (-480.37, 525.75) | 0.131 | -938.35 (-2075.13, 198.43) | 0.048 | -915.66 (-1834.36, 3.03) | 0.283 | 0.149 |
| 1-year visit | 733.74 (420.92) | 748.76 (325.16) | 610.47 (414.4) | 1-year visit – 6-month visit | 295.39 (-169.66, 760.43) | 0.853 | -414.06 (-977.94, 149.83) | 0.731 | -118.67 (-701.17, 463.83) | 0.378 | 0.726 |
|  |  |  |  | 1-year visit – baseline | 318.07 (-369.62, 1005.77) | 0.114 | -1352.41 (-2598.61, -106.21) | 0.093 | -1034.34 (-2141.38, 72.7) | 0.455 | 0.106 |
| **Beta-Alanine** |  |  |  |  |  |  |  |  |  |  |  |
| Baseline | 1355.16 (902.31) | 1245.76 (490.42) | 1279.18 (546.43) |  |  |  |  |  |  |  |  |
| 6-month visit | 1026.1 (559.65) | 999.88 (592.09) | 1173.71 (765.18) | 6-month visit – baseline | 83.18 (-432.67, 599.04) | 0.9 | 140.41 (-402.12, 682.94) | 0.276 | 223.59 (-299.63, 746.82) | 0.308 | 0.808 |
| 1-year visit | 1034.55 (566.34) | 1195.47 (623.34) | 1300.18 (945.4) | 1-year visit – 6-month visit | 187.14 (-333.14, 707.42) | 0.264 | -69.12 (-898.08, 759.84) | 0.382 | 118.02 (-512.31, 748.35) | 0.617 | 0.226 |
|  |  |  |  | 1-year visit – baseline | 270.32 (-347.71, 888.35) | 0.869 | 71.29 (-555.05, 697.64) | 0.56 | 341.61 (-289.32, 972.54) | 0.612 | 0.814 |
| **Erythronic acid** |  |  |  |  |  |  |  |  |  |  |  |
| Baseline | 2029.84 (809.02) | 2247.47 (1076.84) | 2372.88 (1722.59) |  |  |  |  |  |  |  |  |
| 6-month visit | 1825.13 (672.9) | 1771.59 (946.17) | 1982.59 (805.11) | 6-month visit – baseline | -271.17 (-998.04, 455.69) | 0.309 | 85.59 (-1051.1, 1222.28) | 0.636 | -185.58 (-1004.46, 633.29) | 0.877 | 0.299 |
| 1-year visit | 1980.55 (820.79) | 2167.41 (992.27) | 2046.76 (1054.53) | 1-year visit – 6-month visit | 240.4 (-428.59, 909.4) | 0.544 | -331.65 (-1329.68, 666.39) | 0.692 | -91.24 (-765.62, 583.13) | 0.924 | 0.636 |
|  |  |  |  | 1-year visit – baseline | -30.77 (-750.64, 689.1) | 0.476 | -246.06 (-1222.32, 730.21) | 0.786 | -276.83 (-999.05, 445.39) | 0.983 | 0.525 |
| **L-Homoserine** |  |  |  |  |  |  |  |  |  |  |  |
| Baseline | 125.16 (96.74) | 99.24 (28.67) | 150.71 (130.18) |  |  |  |  |  |  |  |  |
| 6-month visit | 123.42 (112.78) | 92.94 (23.39) | 102.53 (26.3) | 6-month visit – baseline | -4.55 (-60.42, 51.32) | 0.959 | -41.88 (-109.3, 25.53) | 0.039 | -46.43 (-119.12, 26.25) | 0.251 | 0.837 |
| 1-year visit | 142.48 (118.38) | 118.06 (58) | 100 (46.7) | 1-year visit – 6-month visit | 6.05 (-61.69, 73.8) | 0.204 | -27.65 (-64.09, 8.8) | 0.914 | -21.59 (-88.66, 45.47) | 0.234 | 0.112 |
|  |  |  |  | 1-year visit – baseline | 1.5 (-47.73, 50.74) | 0.521 | -69.53 (-144.29, 5.23) | 0.17 | -68.03 (-134.26, -1.8) | 0.121 | 0.394 |
| **Capric acid** |  |  |  |  |  |  |  |  |  |  |  |
| Baseline | 1527.84 (1990.97) | 1743.76 (2549.32) | 1760.35 (1308.71) |  |  |  |  |  |  |  |  |
| 6-month visit | 1387.06 (998.74) | 1191.65 (437.12) | 1498.24 (943.85) | 6-month visit – baseline | -411.34 (-1869.31, 1046.62) | 0.448 | 290 (-1276.04, 1856.04) | 0.58 | -121.34 (-1382.03, 1139.34) | 0.929 | 0.612 |
| 1-year visit | 1778.87 (1345.39) | 2089.41 (1970) | 2048.53 (2108.11) | 1-year visit – 6-month visit | 505.96 (-553.62, 1565.53) | 0.299 | -347.47 (-1854.2, 1159.26) | 0.882 | 158.49 (-959.78, 1276.75) | 0.421 | 0.296 |
|  |  |  |  | 1-year visit – baseline | 94.61 (-1577.69, 1766.92) | 0.217 | -57.47 (-1884.48, 1769.54) | 0.815 | 37.14 (-1399.42, 1473.7) | 0.415 | 0.356 |
| **Mandelic acid** |  |  |  |  |  |  |  |  |  |  |  |
| Baseline | 123.65 (21.03) | 107.06 (25.16) | 118 (24.59) |  |  |  |  |  |  |  |  |
| 6-month visit | 101.45 (15.02) | 94.41 (12.17) | 105.06 (17.33) | 6-month visit – baseline | 9.55 (-7.15, 26.24) | 0.898 | -0.29 (-19.94, 19.35) | 0.057 | 9.25 (-7.9, 26.41) | 0.037 | 0.696 |
| 1-year visit | 121.81 (28.02) | 124.59 (23.85) | 100.24 (29.1) | 1-year visit – 6-month visit | 9.82 (-8.32, 27.96) | 0.087 | -35 (-55.22, -14.78) | 0.497 | -25.18 (-45.53, -4.83) | 0.498 | 0.065 |
|  |  |  |  | 1-year visit – baseline | 19.37 (-2.91, 41.65) | 0.025 | -35.29 (-57.73, -12.86) | 0.312 | -15.93 (-37.31, 5.46) | 0.19 | 0.018 |
| **L-Malic acid** |  |  |  |  |  |  |  |  |  |  |  |
| Baseline | 5878.94 (2413.87) | 6550.88 (3135.41) | 4953.71 (1409.62) |  |  |  |  |  |  |  |  |
| 6-month visit | 4601.87 (1075.65) | 4351.65 (1599.96) | 5310.29 (1718.85) | 6-month visit – baseline | -922.17 (-2821.77, 977.43) | 0.996 | 2555.82 (474.1, 4637.54) | 0.724 | 1633.65 (118, 3149.3) | 0.851 | 0.866 |
| 1-year visit | 5858.71 (2061.23) | 6271.35 (2301.79) | 5313.29 (1494.22) | 1-year visit – 6-month visit | 662.87 (-984.26, 2310) | 0.778 | -1916.71 (-3796.45, -36.96) | 0.697 | -1253.84 (-2689.43, 181.75) | 0.935 | 0.96 |
|  |  |  |  | 1-year visit – baseline | -259.3 (-1924.12, 1405.52) | 0.164 | 639.12 (-838.99, 2117.23) | 0.071 | 379.81 (-1244.23, 2003.86) | 0.352 | 0.292 |
| **D-Threitol** |  |  |  |  |  |  |  |  |  |  |  |
| Baseline | 788.03 (559.79) | 669.59 (297.71) | 1098.71 (595.83) |  |  |  |  |  |  |  |  |
| 6-month visit | 742.03 (342.65) | 534.53 (209.75) | 665.94 (530.72) | 6-month visit – baseline | -89.06 (-360.77, 182.65) | 0.469 | -297.71 (-685.64, 90.23) | 0.01 | -386.76 (-753.49, -20.04) | 0.064 | 0.577 |
| 1-year visit | 863.19 (468.1) | 781.41 (404.71) | 530.82 (459.95) | 1-year visit – 6-month visit | 125.72 (-166.72, 418.16) | 0.049 | -382 (-779.88, 15.88) | 0.669 | -256.28 (-589.56, 77) | 0.065 | 0.023 |
|  |  |  |  | 1-year visit – baseline | 36.66 (-281.85, 355.17) | 0.747 | -679.71 (-1070.31, -289.1) | 0.205 | -643.04 (-996.19, -289.9) | 0.396 | 0.657 |
| **L-Aspartic acid** |  |  |  |  |  |  |  |  |  |  |  |
| Baseline | 5148.87 (2746.48) | 14687.65 (10644.42) | 5837 (4910.82) |  |  |  |  |  |  |  |  |
| 6-month visit | 3371.9 (900.94) | 9938.35 (8623.63) | 8620.65 (7044.35) | 6-month visit – baseline | -2972.33 (-6478.97, 534.32) | 0.001 | 7532.94 (1724.49, 13341.39) | 0.033 | 4560.61 (1535.55, 7585.68) | 0 | 0.046 |
| 1-year visit | 4541.74 (2616.88) | 10099.35 (10002.77) | 6270.82 (5672.85) | 1-year visit – 6-month visit | -1008.84 (-4629.48, 2611.8) | 0 | -2510.82 (-9459.29, 4437.64) | 0.215 | -3519.66 (-7546.45, 507.12) | 0.002 | 0.011 |
|  |  |  |  | 1-year visit – baseline | -3981.17 (-6894.85, -1067.48) | 0.114 | 5022.12 (275.76, 9768.47) | 0.014 | 1040.95 (-2175.85, 4257.75) | 0.001 | 0.328 |
| **L-Methionine** |  |  |  |  |  |  |  |  |  |  |  |
| Baseline | 11974.74 (7255.78) | 6166.59 (4276.43) | 13358.65 (7747.17) |  |  |  |  |  |  |  |  |
| 6-month visit | 12697.13 (4409.07) | 7754.59 (6291.87) | 9179.12 (5167.55) | 6-month visit – baseline | 865.61 (-3520.09, 5251.32) | 0.402 | -5767.53 (-11248.52, -286.54) | 0.004 | -4901.92 (-9138.28, -665.55) | 0 | 0.238 |
| 1-year visit | 12786.23 (5028.56) | 12947.35 (7953.97) | 10746.18 (7828.96) | 1-year visit – 6-month visit | 5103.67 (385.61, 9821.72) | 0.016 | -3625.71 (-10012.3, 2760.89) | 0.896 | 1477.96 (-2995.9, 5951.83) | 0.053 | 0.021 |
|  |  |  |  | 1-year visit – baseline | 5969.28 (649.95, 11288.61) | 0.719 | -9393.24 (-15475.41, -3311.06) | 0.08 | -3423.95 (-7976.14, 1128.24) | 0.039 | 0.547 |
| **Pyroglutamic acid** |  |  |  |  |  |  |  |  |  |  |  |
| Baseline | 323842.23 (92618.73) | 428916 (139552.65) | 301861.71 (89631.69) |  |  |  |  |  |  |  |  |
| 6-month visit | 254554.58 (49662.05) | 299317 (101336.7) | 338646.94 (84910.38) | 6-month visit – baseline | -60311.35 (-128577.43, 7954.72) | 0.084 | 166384.24 (83247.62, 249520.85) | 0.112 | 106072.88 (49331.81, 162813.95) | 0.01 | 0.109 |
| 1-year visit | 278758.16 (88093.2) | 327287.76 (121500.97) | 312073.18 (103994.73) | 1-year visit – 6-month visit | 3767.18 (-70067.32, 77601.69) | 0.002 | -54544.53 (-142286.09, 33197.03) | 0.702 | -50777.35 (-112364.8, 10810.11) | 0.079 | 0.006 |
|  |  |  |  | 1-year visit – baseline | -56544.17 (-130894.16, 17805.82) | 0.829 | 111839.71 (19410.83, 204268.59) | 0.029 | 55295.54 (-4579.75, 115170.82) | 0.02 | 0.655 |
| **cis-4-Hydroxy-L-proline** |  |  |  |  |  |  |  |  |  |  |  |
| Baseline | 16160.39 (11254.63) | 15990.82 (7036.82) | 18196.76 (11107.55) |  |  |  |  |  |  |  |  |
| 6-month visit | 16474.94 (8928.51) | 12659.65 (5606.17) | 14347.12 (10041.53) | 6-month visit – baseline | -3645.72 (-11785.97, 4494.52) | 0.656 | -518.47 (-9166.91, 8129.97) | 0.345 | -4164.2 (-13094.78, 4766.39) | 0.095 | 0.605 |
| 1-year visit | 17405.81 (9476.48) | 19836.47 (11779.5) | 14507.12 (7395.9) | 1-year visit – 6-month visit | 6245.95 (-1255.91, 13747.81) | 0.108 | -7016.82 (-16076.15, 2042.5) | 0.327 | -770.87 (-8581, 7039.26) | 0.478 | 0.153 |
|  |  |  |  | 1-year visit – baseline | 2600.23 (-5135.38, 10335.84) | 0.658 | -7535.29 (-16342.82, 1272.23) | 0.506 | -4935.07 (-12156.55, 2286.41) | 0.999 | 0.75 |
| **Threonic acid** |  |  |  |  |  |  |  |  |  |  |  |
| Baseline | 6370.26 (3734.25) | 6586.35 (3195.04) | 6640.12 (5017.13) |  |  |  |  |  |  |  |  |
| 6-month visit | 6905.94 (2361.92) | 5638.59 (1784.15) | 6872.71 (3156.16) | 6-month visit – baseline | -1483.44 (-3679.5, 712.62) | 0.615 | 1180.35 (-2487.62, 4848.32) | 0.49 | -303.09 (-3128.95, 2522.77) | 0.737 | 0.731 |
| 1-year visit | 7225.13 (5134.34) | 7762.18 (2903.73) | 5081.82 (2701.88) | 1-year visit – 6-month visit | 1804.39 (-859.35, 4468.14) | 0.307 | -3914.47 (-6530.34, -1298.6) | 0.415 | -2110.08 (-4935.32, 715.17) | 0.835 | 0.216 |
|  |  |  |  | 1-year visit – baseline | 320.95 (-1818.34, 2460.24) | 0.548 | -2734.12 (-5935.62, 467.39) | 0.306 | -2413.17 (-5118.79, 292.46) | 0.633 | 0.478 |
| **L-Cysteine** |  |  |  |  |  |  |  |  |  |  |  |
| Baseline | 8152.39 (2661.93) | 6172.88 (3046.78) | 8121.82 (1911.6) |  |  |  |  |  |  |  |  |
| 6-month visit | 9118.45 (2314.18) | 7174.76 (3115.86) | 9156 (2759.79) | 6-month visit – baseline | 35.82 (-2146.27, 2217.91) | 0.86 | 32.29 (-2412.7, 2477.28) | 0.006 | 68.11 (-1960.64, 2096.86) | 0.005 | 0.721 |
| 1-year visit | 9313.39 (3500.9) | 9086.53 (2602.45) | 7151.24 (3559.04) | 1-year visit – 6-month visit | 1716.83 (-510.38, 3944.04) | 0.128 | -3916.53 (-6802.5, -1030.56) | 0.718 | -2199.7 (-4786.62, 387.22) | 0.126 | 0.081 |
|  |  |  |  | 1-year visit – baseline | 1752.65 (-934.41, 4439.7) | 0.123 | -3884.24 (-6359.16, -1409.31) | 0.792 | -2131.59 (-4733.66, 470.48) | 0.15 | 0.079 |
| **Creatinine** |  |  |  |  |  |  |  |  |  |  |  |
| Baseline | 8033.74 (3495.16) | 25500.88 (22978.63) | 18862.29 (12849.06) |  |  |  |  |  |  |  |  |
| 6-month visit | 23151.13 (18333.59) | 31980.82 (27891.75) | 31276.35 (34443.58) | 6-month visit – baseline | -8637.45 (-20865.8, 3590.91) | 0.02 | 5934.12 (-16002.16, 27870.4) | 0.507 | -2703.33 (-18976.94, 13570.28) | 0.062 | 0.044 |
| 1-year visit | 19771.97 (15564.96) | 21625.35 (15839.84) | 21209.59 (8802.92) | 1-year visit – 6-month visit | -6976.31 (-22009.28, 8056.67) | 0.252 | 288.71 (-21056.51, 21633.92) | 0.77 | -6687.6 (-24355.87, 10980.67) | 0.84 | 0.324 |
|  |  |  |  | 1-year visit – baseline | -15613.76 (-25057.23, -6170.28) | 0.029 | 6222.82 (-6131.04, 18576.68) | 0.494 | -9390.93 (-19173.44, 391.58) | 0.192 | 0.093 |
| **trans Cinnamic acid** |  |  |  |  |  |  |  |  |  |  |  |
| Baseline | 271.97 (45.68) | 242.41 (73.54) | 243 (51.7) |  |  |  |  |  |  |  |  |
| 6-month visit | 233.45 (46.37) | 200.12 (46.79) | 227 (58.41) | 6-month visit – baseline | -3.78 (-48.22, 40.66) | 0.143 | 26.29 (-36.85, 89.44) | 0.405 | 22.52 (-22.19, 67.22) | 0.071 | 0.136 |
| 1-year visit | 246.71 (53.84) | 240.88 (48.75) | 238.24 (47.32) | 1-year visit – 6-month visit | 27.51 (-11.8, 66.82) | 0.386 | -29.53 (-83.87, 24.81) | 0.344 | -2.02 (-47.08, 43.04) | 0.049 | 0.337 |
|  |  |  |  | 1-year visit – baseline | 23.73 (-24.86, 72.32) | 0.162 | -3.24 (-60.45, 53.98) | 0.561 | 20.49 (-22.7, 63.68) | 0.359 | 0.181 |
| **Oxoglutaric acid** |  |  |  |  |  |  |  |  |  |  |  |
| Baseline | 970.13 (665.4) | 994.24 (733.36) | 856.06 (549.43) |  |  |  |  |  |  |  |  |
| 6-month visit | 899.94 (596.29) | 989.88 (645.39) | 1077.82 (653.24) | 6-month visit – baseline | 65.84 (-462.36, 594.04) | 0.661 | 226.12 (-350.23, 802.46) | 0.904 | 291.96 (-267.13, 851.04) | 0.685 | 0.812 |
| 1-year visit | 1197.84 (781.04) | 1214.18 (781.96) | 1006.35 (659.18) | 1-year visit – 6-month visit | -73.61 (-754.1, 606.88) | 0.956 | -295.76 (-983.99, 392.46) | 0.943 | -369.37 (-997.54, 258.79) | 0.68 | 0.9 |
|  |  |  |  | 1-year visit – baseline | -7.77 (-656.64, 641.1) | 0.405 | -69.65 (-764.25, 624.96) | 0.357 | -77.42 (-720.23, 565.4) | 0.707 | 0.415 |
| **L-Glutamic acid** |  |  |  |  |  |  |  |  |  |  |  |
| Baseline | 16310.35 (13709.2) | 46700.41 (28340.05) | 20953.53 (25185.4) |  |  |  |  |  |  |  |  |
| 6-month visit | 12192.97 (4479.23) | 24587.88 (22077.63) | 19126 (17058.94) | 6-month visit – baseline | -17995.14 (-30665.3, -5324.99) | 0.156 | 20285 (3883.71, 36686.29) | 0.016 | 2289.86 (-6461.61, 11041.32) | 0 | 0.133 |
| 1-year visit | 15696.77 (13681.16) | 17461.88 (12019.4) | 21853.12 (22147.69) | 1-year visit – 6-month visit | -10629.81 (-22336.89, 1077.28) | 0.088 | 9853.12 (-4304.47, 24010.71) | 0.758 | -776.69 (-8928.66, 7375.29) | 0.075 | 0.074 |
|  |  |  |  | 1-year visit – baseline | -28624.95 (-43095.57, -14154.33) | 0.277 | 30138.12 (13826.49, 46449.75) | 0.034 | 1513.17 (-9177.6, 12203.94) | 0.001 | 0.153 |
| **L-Phenylalanine** |  |  |  |  |  |  |  |  |  |  |  |
| Baseline | 42159.81 (12589.63) | 49634.06 (17410.76) | 54762.47 (20735.46) |  |  |  |  |  |  |  |  |
| 6-month visit | 44158.97 (12504.34) | 44764.35 (14694) | 48595.94 (14381.13) | 6-month visit – baseline | -6868.87 (-15752.65, 2014.92) | 0.007 | -1296.82 (-16093.01, 13499.36) | 0.336 | -8165.69 (-20314.34, 3982.95) | 0.633 | 0.025 |
| 1-year visit | 49332 (10593.32) | 55413.76 (16444.04) | 43298.24 (17612.5) | 1-year visit – 6-month visit | 5476.38 (-3626.68, 14579.43) | 0.687 | -15947.12 (-30744.01, -1150.22) | 0.45 | -10470.74 (-22189.4, 1247.92) | 0.818 | 0.618 |
|  |  |  |  | 1-year visit – baseline | -1392.49 (-11266.64, 8481.67) | 0.33 | -17243.94 (-31618.61, -2869.28) | 0.725 | -18636.43 (-30403.48, -6869.38) | 0.257 | 0.409 |
| **4 Hydroxy 3 methoxybenzenemethanol** |  |  |  |  |  |  |  |  |  |  |  |
| Baseline | 169.39 (95.71) | 240.59 (195.28) | 178.94 (65.81) |  |  |  |  |  |  |  |  |
| 6-month visit | 156.74 (33.82) | 185.18 (98.08) | 172.18 (97.43) | 6-month visit – baseline | -42.77 (-138.17, 52.64) | 0.612 | 48.65 (-75.73, 173.02) | 0.089 | 5.88 (-65.24, 77) | 0.046 | 0.404 |
| 1-year visit | 165.84 (58.92) | 168.29 (49.78) | 181.59 (94.11) | 1-year visit – 6-month visit | -25.98 (-75.39, 23.43) | 0.42 | 26.29 (-41.92, 94.51) | 0.962 | 0.31 (-51.84, 52.47) | 0.603 | 0.414 |
|  |  |  |  | 1-year visit – baseline | -68.75 (-153.75, 16.26) | 0.759 | 74.94 (-34.67, 184.55) | 0.267 | 6.2 (-55.52, 67.91) | 0.206 | 0.543 |
| **D-Xylose** |  |  |  |  |  |  |  |  |  |  |  |
| Baseline | 967.87 (524.87) | 1730.12 (1556.39) | 1383.35 (1734.18) |  |  |  |  |  |  |  |  |
| 6-month visit | 1444.94 (1653.56) | 850.88 (277.34) | 1763.47 (1708.55) | 6-month visit – baseline | -1356.3 (-2275.61, -436.99) | 0.369 | 1259.35 (67.17, 2451.54) | 0.378 | -96.95 (-1060.75, 866.86) | 0.747 | 0.333 |
| 1-year visit | 892.16 (974.47) | 1104.35 (1299.86) | 1030.41 (1287.31) | 1-year visit – 6-month visit | 806.24 (-235.4, 1847.89) | 0.486 | -986.53 (-2249.66, 276.6) | 0.156 | -180.28 (-1440.73, 1080.17) | 0.589 | 0.526 |
|  |  |  |  | 1-year visit – baseline | -550.06 (-1522.23, 422.12) | 0.219 | 272.82 (-1262.79, 1808.44) | 0.529 | -277.23 (-1271.48, 717.02) | 0.036 | 0.215 |
| **Dodecanoic acid** |  |  |  |  |  |  |  |  |  |  |  |
| Baseline | 3419.52 (4094.28) | 3506.47 (3861.6) | 3054.24 (1800.13) |  |  |  |  |  |  |  |  |
| 6-month visit | 3107.45 (2274.21) | 2456.06 (1970.04) | 2344.12 (1240.31) | 6-month visit – baseline | -738.35 (-3468.68, 1991.99) | 0.519 | 340.29 (-2100.66, 2781.25) | 0.526 | -398.05 (-2691.42, 1895.32) | 0.895 | 0.467 |
| 1-year visit | 3643.45 (4181.92) | 3565.41 (3108.3) | 2639.59 (1271.12) | 1-year visit – 6-month visit | 573.35 (-1848.91, 2995.61) | 0.352 | -813.88 (-2923.03, 1295.26) | 0.159 | -240.53 (-2329.86, 1848.8) | 0.653 | 0.317 |
|  |  |  |  | 1-year visit – baseline | -164.99 (-3304.74, 2974.75) | 0.64 | -473.59 (-3118.24, 2171.06) | 0.254 | -638.58 (-3366.81, 2089.65) | 0.532 | 0.566 |
| **L-Arabinose** |  |  |  |  |  |  |  |  |  |  |  |
| Baseline | 3940.48 (1224.21) | 3675.06 (932.03) | 3528.88 (1100.37) |  |  |  |  |  |  |  |  |
| 6-month visit | 3153.77 (680.8) | 3039.18 (593.82) | 3304.94 (875.92) | 6-month visit – baseline | 150.83 (-546.01, 847.67) | 0.512 | 411.94 (-473.35, 1297.23) | 0.652 | 562.77 (-304.74, 1430.27) | 0.351 | 0.454 |
| 1-year visit | 3833.42 (1178.21) | 3827 (1595.4) | 3253.59 (1149.2) | 1-year visit – 6-month visit | 108.18 (-712.76, 929.12) | 0.248 | -839.18 (-1887.12, 208.76) | 0.924 | -731 (-1597.42, 135.42) | 0.286 | 0.211 |
|  |  |  |  | 1-year visit – baseline | 259.01 (-749.41, 1267.42) | 0.058 | -427.24 (-1344.27, 489.8) | 0.537 | -168.23 (-1089.87, 753.42) | 0.249 | 0.053 |
| **Oxoadipic acid** |  |  |  |  |  |  |  |  |  |  |  |
| Baseline | 1098.81 (550.91) | 1091.71 (648.97) | 1290.53 (1439.85) |  |  |  |  |  |  |  |  |
| 6-month visit | 1484.52 (1756.96) | 908.35 (468.76) | 1745.35 (2099.53) | 6-month visit – baseline | -569.06 (-1473.15, 335.03) | 0.584 | 638.18 (-425.87, 1702.22) | 0.121 | 69.11 (-1033.54, 1171.76) | 0.395 | 0.613 |
| 1-year visit | 1047.52 (619.97) | 963.18 (416.41) | 1283.65 (1896.61) | 1-year visit – 6-month visit | 491.82 (-461.81, 1445.46) | 0.544 | -516.53 (-2004.29, 971.23) | 0.083 | -24.71 (-1439.86, 1390.45) | 0.227 | 0.581 |
|  |  |  |  | 1-year visit – baseline | -77.24 (-580.27, 425.79) | 0.353 | 121.65 (-1150.58, 1393.88) | 0.435 | 44.41 (-919.48, 1008.3) | 0.639 | 0.35 |
| **Homocysteine** |  |  |  |  |  |  |  |  |  |  |  |
| Baseline | 176.19 (91.07) | 353.76 (145.55) | 209.65 (108.49) |  |  |  |  |  |  |  |  |
| 6-month visit | 113.55 (38.54) | 252.82 (157.74) | 223.59 (137.33) | 6-month visit – baseline | -38.3 (-121.62, 45.02) | 0.004 | 114.88 (-2.98, 232.74) | 0.027 | 76.59 (8.92, 144.25) | 0 | 0.01 |
| 1-year visit | 128.19 (71.58) | 277.41 (236.66) | 192.47 (133.22) | 1-year visit – 6-month visit | 9.94 (-90.08, 109.96) | 0 | -55.71 (-221.77, 110.35) | 0.273 | -45.76 (-131.16, 39.64) | 0 | 0.004 |
|  |  |  |  | 1-year visit – baseline | -28.35 (-145.49, 88.79) | 0.027 | 59.18 (-101.81, 220.16) | 0.011 | 30.82 (-63.6, 125.25) | 0 | 0.059 |
| **L-Asparagine** |  |  |  |  |  |  |  |  |  |  |  |
| Baseline | 12655.45 (5231.34) | 9819.18 (3933.36) | 11410.41 (6026.68) |  |  |  |  |  |  |  |  |
| 6-month visit | 11014.45 (3686.87) | 8644.82 (3077.89) | 11298.24 (3194.55) | 6-month visit – baseline | 466.65 (-2643.62, 3576.91) | 0.569 | 1062.18 (-2681.87, 4806.22) | 0.023 | 1528.82 (-1657.43, 4715.07) | 0.004 | 0.355 |
| 1-year visit | 12694.84 (4167.46) | 14536.12 (3844.82) | 9838.53 (4846.38) | 1-year visit – 6-month visit | 4210.91 (968.16, 7453.65) | 0.122 | -7351 (-10672.86, -4029.14) | 0.704 | -3140.09 (-6314.85, 34.66) | 0.634 | 0.102 |
|  |  |  |  | 1-year visit – baseline | 4677.55 (1077.71, 8277.4) | 0.063 | -6288.82 (-9988.03, -2589.62) | 0.545 | -1611.27 (-5360.74, 2138.2) | 0.539 | 0.043 |
| **Taurine** |  |  |  |  |  |  |  |  |  |  |  |
| Baseline | 279.87 (109.2) | 293.06 (210.93) | 702.29 (1001.78) |  |  |  |  |  |  |  |  |
| 6-month visit | 393.65 (285.33) | 270.12 (152.93) | 540.12 (469.82) | 6-month visit – baseline | -136.72 (-300.95, 27.52) | 0.013 | -139.24 (-707.12, 428.65) | 0.012 | -275.95 (-700.11, 148.21) | 0.449 | 0.011 |
| 1-year visit | 355.23 (159.23) | 377 (290.55) | 424.59 (324.44) | 1-year visit – 6-month visit | 145.3 (-42.78, 333.38) | 0.086 | -222.41 (-504.07, 59.25) | 0.145 | -77.11 (-302.24, 148.02) | 0.962 | 0.084 |
|  |  |  |  | 1-year visit – baseline | 8.59 (-163.67, 180.84) | 0.026 | -361.65 (-901.6, 178.3) | 0.117 | -353.06 (-729.57, 23.45) | 0.374 | 0.015 |
| **D-Ribose** |  |  |  |  |  |  |  |  |  |  |  |
| Baseline | 415.52 (186.07) | 269.82 (113.76) | 325.65 (168.6) |  |  |  |  |  |  |  |  |
| 6-month visit | 246.06 (100.51) | 175.94 (145.68) | 245.06 (132.84) | 6-month visit – baseline | 75.57 (-59.58, 210.72) | 0.232 | 13.29 (-136.85, 163.44) | 0.095 | 88.86 (-50.54, 228.27) | 0.008 | 0.136 |
| 1-year visit | 355.26 (186.47) | 310.12 (130.52) | 230.65 (126.8) | 1-year visit – 6-month visit | 24.98 (-102.23, 152.19) | 0.054 | -148.59 (-267.39, -29.79) | 0.987 | -123.61 (-239.74, -7.47) | 0.197 | 0.041 |
|  |  |  |  | 1-year visit – baseline | 100.55 (-56.2, 257.3) | 0.01 | -135.29 (-276.43, 5.85) | 0.75 | -34.74 (-200.29, 130.81) | 0.027 | 0.003 |
| **L-Arabitol** |  |  |  |  |  |  |  |  |  |  |  |
| Baseline | 7247.81 (8673.78) | 3868.06 (1465.75) | 5605.88 (1840.96) |  |  |  |  |  |  |  |  |
| 6-month visit | 6337.13 (6565.91) | 3665.35 (1649.83) | 5073.24 (3547.27) | 6-month visit – baseline | 707.97 (-2729.19, 4145.14) | 0.33 | -329.94 (-2255.92, 1596.04) | 0.019 | 378.03 (-3199.17, 3955.23) | 0.047 | 0.211 |
| 1-year visit | 7086.45 (8003.87) | 5560.53 (2209.66) | 4734.94 (3604.97) | 1-year visit – 6-month visit | 1145.85 (-3708.26, 5999.97) | 0.206 | -2233.47 (-4888.91, 421.97) | 0.639 | -1087.62 (-6189.22, 4013.98) | 0.121 | 0.115 |
|  |  |  |  | 1-year visit – baseline | 1853.83 (-3608.47, 7316.13) | 0.209 | -2563.41 (-4842.98, -283.85) | 0.374 | -709.59 (-6296.91, 4877.74) | 0.109 | 0.112 |
| **Ribitol** |  |  |  |  |  |  |  |  |  |  |  |
| Baseline | 567.77 (195.31) | 556.94 (229.82) | 689.71 (192.53) |  |  |  |  |  |  |  |  |
| 6-month visit | 465.87 (134) | 385.24 (108.49) | 511.12 (96.86) | 6-month visit – baseline | -69.8 (-218.21, 78.6) | 0.019 | -6.88 (-164.68, 150.91) | 0.017 | -76.69 (-212.71, 59.34) | 0.243 | 0.048 |
| 1-year visit | 574.35 (182.33) | 636.06 (187.96) | 526.35 (187.23) | 1-year visit – 6-month visit | 142.34 (18.85, 265.82) | 0.97 | -235.59 (-382.52, -88.66) | 0.669 | -93.25 (-217.3, 30.81) | 0.575 | 0.877 |
|  |  |  |  | 1-year visit – baseline | 72.54 (-70.35, 215.42) | 0.322 | -242.47 (-423.61, -61.33) | 0.714 | -169.93 (-337.39, -2.48) | 0.833 | 0.432 |
| **Rhamnose** |  |  |  |  |  |  |  |  |  |  |  |
| Baseline | 2963.39 (1207.7) | 3141.88 (2240.19) | 3741.12 (1885.16) |  |  |  |  |  |  |  |  |
| 6-month visit | 3170.45 (875.07) | 3950.65 (5748.7) | 2873.18 (919.21) | 6-month visit – baseline | 601.7 (-1703.05, 2906.45) | 0.333 | -1676.71 (-4956, 1602.58) | 0.672 | -1075.01 (-1995.91, -154.1) | 0.151 | 0.43 |
| 1-year visit | 3061.16 (1176.88) | 2749.65 (857.56) | 3222.24 (1232.12) | 1-year visit – 6-month visit | -1091.71 (-3272.32, 1088.9) | 0.964 | 1550.06 (-1439.67, 4539.78) | 0.61 | 458.35 (-210.17, 1126.87) | 0.273 | 0.842 |
|  |  |  |  | 1-year visit – baseline | -490.01 (-1480.41, 500.39) | 0.159 | -126.65 (-1677.15, 1423.86) | 0.137 | -616.66 (-1566.61, 333.3) | 0.859 | 0.246 |
| **L-Fucose** |  |  |  |  |  |  |  |  |  |  |  |
| Baseline | 532.58 (124.96) | 832.71 (581.28) | 857.06 (558.36) |  |  |  |  |  |  |  |  |
| 6-month visit | 638.65 (213.05) | 670.76 (562.91) | 703.82 (202.27) | 6-month visit – baseline | -268.01 (-594.36, 58.35) | 0.003 | 8.71 (-501.22, 518.63) | 0.892 | -259.3 (-497.04, -21.56) | 0.021 | 0.011 |
| 1-year visit | 691.26 (260.34) | 588.41 (239.41) | 699.29 (246.19) | 1-year visit – 6-month visit | -134.97 (-386.03, 116.1) | 0.41 | 77.82 (-233.14, 388.78) | 0.538 | -57.14 (-233.82, 119.53) | 0.947 | 0.512 |
|  |  |  |  | 1-year visit – baseline | -402.97 (-649.33, -156.61) | 0.028 | 86.53 (-310.15, 483.21) | 0.513 | -316.44 (-544.69, -88.2) | 0.273 | 0.05 |
| **cis Aconitic acid** |  |  |  |  |  |  |  |  |  |  |  |
| Baseline | 300.77 (82.42) | 306.41 (224.71) | 299.71 (132.02) |  |  |  |  |  |  |  |  |
| 6-month visit | 312.58 (133.16) | 273.88 (102.9) | 220.18 (94.15) | 6-month visit – baseline | -44.34 (-167.67, 79) | 0.076 | -47 (-185.71, 91.71) | 0.564 | -91.34 (-185.45, 2.78) | 0.654 | 0.11 |
| 1-year visit | 328.19 (125.47) | 282.29 (145.24) | 312.35 (132.62) | 1-year visit – 6-month visit | -7.2 (-106.89, 92.49) | 0.042 | 83.76 (-25.45, 192.98) | 0.856 | 76.56 (-26.64, 179.77) | 0.116 | 0.04 |
|  |  |  |  | 1-year visit – baseline | -51.54 (-158.53, 55.45) | 0.725 | 36.76 (-98.53, 172.06) | 0.735 | -14.77 (-112.28, 82.73) | 0.39 | 0.736 |
| **Glycerol 3-phosphate** |  |  |  |  |  |  |  |  |  |  |  |
| Baseline | 8350.61 (2320) | 8025.65 (3571.06) | 7326.76 (3053.55) |  |  |  |  |  |  |  |  |
| 6-month visit | 4315.45 (1794.38) | 4839.76 (2019.68) | 7488.71 (2882.62) | 6-month visit – baseline | 849.28 (-795.1, 2493.66) | 0.047 | 3347.82 (687.87, 6007.78) | 0.342 | 4197.1 (2328.01, 6066.2) | 0.733 | 0.074 |
| 1-year visit | 6946.55 (1960.02) | 10198.35 (5997.36) | 4643.47 (1612.3) | 1-year visit – 6-month visit | 2727.49 (671.05, 4783.93) | 0.26 | -8203.82 (-11363.49, -5044.16) | 0.227 | -5476.33 (-7215.61, -3737.06) | 0.051 | 0.426 |
|  |  |  |  | 1-year visit – baseline | 3576.77 (1429.95, 5723.59) | 0.006 | -4856 (-7750.22, -1961.78) | 0.014 | -1279.23 (-3253.2, 694.74) | 0.157 | 0.112 |
| **L-Glutamine** |  |  |  |  |  |  |  |  |  |  |  |
| Baseline | 308811.06 (108862.57) | 172493 (82500.63) | 330060.59 (151133.8) |  |  |  |  |  |  |  |  |
| 6-month visit | 326601.42 (75516.73) | 227656.41 (117199.19) | 310070.12 (107959.17) | 6-month visit – baseline | 37373.06 (-33469.94, 108216.05) | 0.99 | -75153.88 (-161250.64, 10942.88) | 0 | -37780.83 (-103067.56, 27505.91) | 0 | 0.612 |
| 1-year visit | 335615.97 (85713.03) | 331942.65 (87456.34) | 268493 (127581.1) | 1-year visit – 6-month visit | 95271.69 (20923.41, 169619.96) | 0.051 | -145863.35 (-232451.73, -59274.98) | 0.382 | -50591.67 (-122687.43, 21504.1) | 0.014 | 0.033 |
|  |  |  |  | 1-year visit – baseline | 132644.74 (49516.62, 215772.87) | 0.369 | -221017.24 (-297759.13, -144275.34) | 0.05 | -88372.49 (-162512.12, -14232.86) | 0.006 | 0.204 |
| **O-Phosphoethanolamine** |  |  |  |  |  |  |  |  |  |  |  |
| Baseline | 1370.65 (528.59) | 869.59 (260.49) | 1187.82 (432.22) |  |  |  |  |  |  |  |  |
| 6-month visit | 1148.03 (418.3) | 768.82 (254.7) | 1037.41 (428.05) | 6-month visit – baseline | 121.85 (-214.66, 458.36) | 0.145 | -49.65 (-333.57, 234.28) | 0.005 | 72.2 (-284.75, 429.15) | 0 | 0.077 |
| 1-year visit | 1145.48 (371.87) | 1063.82 (434.65) | 951.12 (490.61) | 1-year visit – 6-month visit | 297.55 (-33.8, 628.9) | 0.071 | -381.29 (-823, 60.41) | 0.344 | -83.75 (-456.09, 288.6) | 0.021 | 0.045 |
|  |  |  |  | 1-year visit – baseline | 419.4 (62.21, 776.58) | 0.096 | -430.94 (-869.16, 7.28) | 0.342 | -11.54 (-414.1, 391.01) | 0.019 | 0.047 |
| **Azelaic acid** |  |  |  |  |  |  |  |  |  |  |  |
| Baseline | 368.68 (210.82) | 2460.53 (3433.14) | 848.71 (1752.43) |  |  |  |  |  |  |  |  |
| 6-month visit | 422.23 (188.49) | 2528.65 (3388.18) | 2532.94 (3385.34) | 6-month visit – baseline | 14.57 (-923.14, 952.28) | 0.001 | 1616.12 (-397.98, 3630.22) | 0.436 | 1630.69 (492.58, 2768.8) | 0.005 | 0.027 |
| 1-year visit | 365.1 (203.6) | 2506.18 (3405.46) | 1241.94 (2239.22) | 1-year visit – 6-month visit | 34.66 (-870.35, 939.67) | 0 | -1268.53 (-3804.35, 1267.29) | 0.531 | -1233.87 (-2846.44, 378.7) | 0.003 | 0.007 |
|  |  |  |  | 1-year visit – baseline | 49.23 (-878.15, 976.61) | 0.004 | 347.59 (-1579.88, 2275.05) | 0.144 | 396.82 (-665.34, 1458.97) | 0.004 | 0.152 |
| **3-Phosphoglyceric acid** |  |  |  |  |  |  |  |  |  |  |  |
| Baseline | 361.9 (150.66) | 309.94 (138.86) | 293.53 (125.86) |  |  |  |  |  |  |  |  |
| 6-month visit | 350.23 (179.39) | 290.88 (162.73) | 352.06 (208.72) | 6-month visit – baseline | -7.38 (-148.59, 133.83) | 0.286 | 77.59 (-98.94, 254.12) | 0.539 | 70.21 (-83.58, 224) | 0.021 | 0.196 |
| 1-year visit | 306.87 (178.93) | 299.35 (111.41) | 356.41 (160.09) | 1-year visit – 6-month visit | 51.83 (-80.33, 183.98) | 0.516 | -4.12 (-182.38, 174.14) | 0.158 | 47.71 (-101.41, 196.82) | 0.211 | 0.634 |
|  |  |  |  | 1-year visit – baseline | 44.44 (-89.05, 177.94) | 0.589 | 73.47 (-61.04, 207.98) | 0.375 | 117.91 (-22.82, 258.65) | 0.127 | 0.479 |
| **Hippuric acid** |  |  |  |  |  |  |  |  |  |  |  |
| Baseline | 679.9 (99.77) | 623.06 (97.34) | 606.94 (160.25) |  |  |  |  |  |  |  |  |
| 6-month visit | 557.52 (139.19) | 627 (147) | 574.53 (116.27) | 6-month visit – baseline | 126.33 (18.52, 234.14) | 0.393 | -36.35 (-166.85, 94.14) | 0.413 | 89.98 (-24.21, 204.16) | 0.418 | 0.438 |
| 1-year visit | 680.84 (176.1) | 703.53 (119.56) | 647.12 (164.71) | 1-year visit – 6-month visit | -46.79 (-187.99, 94.4) | 0.722 | -3.94 (-134.35, 126.47) | 0.241 | -50.73 (-192.92, 91.45) | 0.341 | 0.794 |
|  |  |  |  | 1-year visit – baseline | 79.54 (-27.84, 186.91) | 0.175 | -40.29 (-164.52, 83.93) | 0.465 | 39.24 (-81.87, 160.35) | 0.66 | 0.123 |
| **1-Methylhistidine** |  |  |  |  |  |  |  |  |  |  |  |
| Baseline | 294.94 (153.53) | 183.18 (78.82) | 288.29 (138.68) |  |  |  |  |  |  |  |  |
| 6-month visit | 169.32 (97.21) | 182.71 (106.14) | 238.24 (117.71) | 6-month visit – baseline | 125.14 (27.12, 223.17) | 0.332 | -49.59 (-144.57, 45.39) | 0.014 | 75.55 (-27.66, 178.77) | 0.042 | 0.492 |
| 1-year visit | 247.16 (121.88) | 234.59 (96.3) | 191 (81) | 1-year visit – 6-month visit | -25.96 (-124.53, 72.61) | 0.772 | -99.12 (-199.18, 0.95) | 0.81 | -125.07 (-223.83, -26.32) | 0.826 | 0.831 |
|  |  |  |  | 1-year visit – baseline | 99.19 (-4.96, 203.33) | 0.276 | -148.71 (-239.4, -58.01) | 0.199 | -49.52 (-155.61, 56.57) | 0.022 | 0.16 |
| **Hypoxanthine** |  |  |  |  |  |  |  |  |  |  |  |
| Baseline | 1578.42 (2251.09) | 2367 (2661.23) | 741.59 (340.41) |  |  |  |  |  |  |  |  |
| 6-month visit | 2350.1 (3660.2) | 1613.82 (1164.8) | 1209.47 (796.89) | 6-month visit – baseline | -1524.85 (-3699.54, 649.83) | 0.044 | 1221.06 (140.97, 2301.15) | 0.084 | -303.8 (-2375.86, 1768.27) | 0.499 | 0.035 |
| 1-year visit | 843.65 (345.94) | 1555.59 (1787.13) | 1817.06 (2544.58) | 1-year visit – 6-month visit | 1448.22 (-558.86, 3455.29) | 0.787 | 665.82 (-1092.43, 2424.08) | 0.837 | 2114.04 (-26.63, 4254.71) | 0.547 | 0.71 |
|  |  |  |  | 1-year visit – baseline | -76.64 (-1568.18, 1414.91) | 0.803 | 1886.88 (56.29, 3717.47) | 0.266 | 1810.24 (334.18, 3286.31) | 0.284 | 0.845 |
| **Citric acid** |  |  |  |  |  |  |  |  |  |  |  |
| Baseline | 110897.39 (25096.09) | 98672.53 (56416.28) | 110856.71 (30840.18) |  |  |  |  |  |  |  |  |
| 6-month visit | 103157.94 (22813.54) | 80398.94 (34233.98) | 85923 (32908.04) | 6-month visit – baseline | -10534.14 (-32113.73, 11045.46) | 0.187 | -6660.12 (-38834.03, 25513.8) | 0.358 | -17194.25 (-37091.66, 2703.15) | 0.187 | 0.275 |
| 1-year visit | 113401.65 (41310.74) | 98862.47 (47435.81) | 99435.47 (36974.81) | 1-year visit – 6-month visit | 8219.82 (-16953.57, 33393.21) | 0.078 | -4951.06 (-40302.7, 30400.58) | 0.539 | 3268.76 (-23350.83, 29888.35) | 0.156 | 0.067 |
|  |  |  |  | 1-year visit – baseline | -2314.32 (-31361.07, 26732.44) | 0.466 | -11611.18 (-46035.48, 22813.13) | 0.498 | -13925.49 (-39154.19, 11303.2) | 0.463 | 0.464 |
| **Ornithine** |  |  |  |  |  |  |  |  |  |  |  |
| Baseline | 138785.32 (71346.15) | 141919.35 (52702.39) | 161530.29 (89827.94) |  |  |  |  |  |  |  |  |
| 6-month visit | 128298.84 (60701.5) | 106184.88 (28969.36) | 121371.71 (50625.11) | 6-month visit – baseline | -25247.99 (-65588.17, 15092.2) | 0.921 | -4424.12 (-58619.72, 49771.48) | 0.104 | -29672.1 (-74934.49, 15590.28) | 0.319 | 0.973 |
| 1-year visit | 161599.81 (101442.18) | 145641.88 (81029.82) | 124398.76 (39550.58) | 1-year visit – 6-month visit | 6156.03 (-45350.17, 57662.23) | 0.174 | -36429.94 (-88391.25, 15531.37) | 0.827 | -30273.91 (-77155.32, 16607.5) | 0.187 | 0.115 |
|  |  |  |  | 1-year visit – baseline | -19091.95 (-68327.15, 30143.24) | 0.52 | -40854.06 (-104415.01, 22706.89) | 0.663 | -59946.01 (-110279.89, -9612.14) | 0.486 | 0.473 |
| **Citrulline** |  |  |  |  |  |  |  |  |  |  |  |
| Baseline | 3764.06 (1906.09) | 3677.41 (1672.76) | 5788.47 (2231.86) |  |  |  |  |  |  |  |  |
| 6-month visit | 4132.97 (1517.02) | 4011.18 (1762.71) | 3938.47 (1367.35) | 6-month visit – baseline | -35.14 (-1293.29, 1223.01) | 0.056 | -2183.76 (-3786.76, -580.77) | 0.046 | -2218.9 (-3412.96, -1024.85) | 0.533 | 0.061 |
| 1-year visit | 4142.68 (2041.67) | 4391.18 (1780.3) | 4243.35 (2466.76) | 1-year visit – 6-month visit | 370.29 (-794.18, 1534.76) | 0.803 | -75.12 (-1852.59, 1702.36) | 0.909 | 295.17 (-1200.22, 1790.56) | 0.637 | 0.716 |
|  |  |  |  | 1-year visit – baseline | 335.15 (-1111.22, 1781.52) | 0.064 | -2258.88 (-4210.61, -307.15) | 0.048 | -1923.73 (-3537.38, -310.08) | 0.764 | 0.063 |
| **Homogentisic acid** |  |  |  |  |  |  |  |  |  |  |  |
| Baseline | 1312.16 (623.56) | 1025.76 (593.74) | 1405.35 (1063.47) |  |  |  |  |  |  |  |  |
| 6-month visit | 1044.71 (796.05) | 634.41 (345.5) | 1315.29 (842.55) | 6-month visit – baseline | -123.9 (-540.57, 292.77) | 0.499 | 301.29 (-328.85, 931.44) | 0.007 | 177.39 (-339.82, 694.61) | 0.035 | 0.656 |
| 1-year visit | 1161.13 (828.95) | 1391.82 (1095.8) | 1113.53 (1755.14) | 1-year visit – 6-month visit | 640.99 (-110.86, 1392.85) | 0.747 | -959.18 (-2152.4, 234.05) | 0.537 | -318.18 (-1303.14, 666.77) | 0.714 | 0.701 |
|  |  |  |  | 1-year visit – baseline | 517.09 (-179.87, 1214.05) | 0.993 | -657.88 (-1883.92, 568.16) | 0.997 | -140.79 (-1058.78, 777.2) | 0.986 | 0.935 |
| **Myristoleic acid** |  |  |  |  |  |  |  |  |  |  |  |
| Baseline | 1093.32 (680.1) | 1247 (688.84) | 1355.53 (682.41) |  |  |  |  |  |  |  |  |
| 6-month visit | 1399 (685.9) | 1189.06 (550.67) | 1471 (980.56) | 6-month visit – baseline | -363.62 (-985.36, 258.12) | 0.374 | 173.41 (-559.92, 906.74) | 0.363 | -190.21 (-871.47, 491.05) | 0.467 | 0.391 |
| 1-year visit | 1417.61 (1014.12) | 1463.88 (848.79) | 1795.82 (3271.32) | 1-year visit – 6-month visit | 256.21 (-431.37, 943.79) | 0.525 | 50 (-1630.08, 1730.08) | 0.588 | 306.21 (-1009.83, 1622.25) | 0.272 | 0.529 |
|  |  |  |  | 1-year visit – baseline | -107.41 (-625.32, 410.5) | 0.373 | 223.41 (-1410.03, 1856.85) | 0.673 | 116 (-1095.76, 1327.77) | 0.864 | 0.351 |
| **Quinic acid** |  |  |  |  |  |  |  |  |  |  |  |
| Baseline | 104477.13 (76820.83) | 121872.88 (99861.8) | 79982.71 (47148.57) |  |  |  |  |  |  |  |  |
| 6-month visit | 105629.42 (63987.57) | 101587.18 (82590.88) | 117905.06 (57762.41) | 6-month visit – baseline | -21438 (-78801.8, 35925.81) | 0.79 | 58208.06 (-7623.92, 124040.04) | 0.613 | 36770.06 (-17620.89, 91161.01) | 0.991 | 0.716 |
| 1-year visit | 108449.48 (78277.66) | 69680.35 (57735.9) | 105345.76 (69764.97) | 1-year visit – 6-month visit | -34726.89 (-98102.88, 28649.11) | 0.75 | 19347.53 (-40259.8, 78954.86) | 0.177 | -15379.36 (-76479.2, 45720.48) | 0.153 | 0.932 |
|  |  |  |  | 1-year visit – baseline | -56164.88 (-121818.18, 9488.41) | 0.448 | 77555.59 (3693.63, 151417.54) | 0.983 | 21390.7 (-39715.55, 82496.96) | 0.459 | 0.336 |
| **Myristic acid** |  |  |  |  |  |  |  |  |  |  |  |
| Baseline | 692.16 (536.87) | 717.24 (491.72) | 964.88 (670.54) |  |  |  |  |  |  |  |  |
| 6-month visit | 784.32 (568.71) | 540.24 (379.55) | 756.29 (741.76) | 6-month visit – baseline | -269.16 (-654.83, 116.5) | 0.191 | -31.59 (-514.18, 451.01) | 0.29 | -300.75 (-726.37, 124.88) | 0.777 | 0.245 |
| 1-year visit | 953.03 (885.99) | 811.53 (418.72) | 746.47 (440.26) | 1-year visit – 6-month visit | 102.58 (-389.53, 594.69) | 0.762 | -281.12 (-819.64, 257.41) | 0.793 | -178.53 (-732.86, 375.79) | 0.762 | 0.62 |
|  |  |  |  | 1-year visit – baseline | -166.58 (-661.61, 328.45) | 0.441 | -312.71 (-770.47, 145.06) | 0.637 | -479.28 (-1026, 67.43) | 0.757 | 0.56 |
| **Methionine sulfoxide** |  |  |  |  |  |  |  |  |  |  |  |
| Baseline | 1399.45 (1004.45) | 3836.71 (1837.26) | 1732.24 (1014.94) |  |  |  |  |  |  |  |  |
| 6-month visit | 894.77 (492.61) | 2693.59 (1640.83) | 1878.59 (1175.58) | 6-month visit – baseline | -638.44 (-1661.76, 384.88) | 0.005 | 1289.47 (-153.67, 2732.61) | 0 | 651.03 (-98.32, 1400.38) | 0 | 0.021 |
| 1-year visit | 1167.87 (868.63) | 2722 (2445.4) | 1990.71 (1916.65) | 1-year visit – 6-month visit | -244.69 (-1299.93, 810.56) | 0 | 83.71 (-1687.9, 1855.31) | 0.087 | -160.98 (-1094.27, 772.31) | 0 | 0.005 |
|  |  |  |  | 1-year visit – baseline | -883.13 (-2282.23, 515.98) | 0.046 | 1373.18 (-584.2, 3330.56) | 0.005 | 490.05 (-542.21, 1522.31) | 0 | 0.057 |
| **L-Sorbose** |  |  |  |  |  |  |  |  |  |  |  |
| Baseline | 48159.16 (15832.68) | 43849.53 (44431.43) | 29431.12 (16043.76) |  |  |  |  |  |  |  |  |
| 6-month visit | 38320.29 (21727.19) | 35629.47 (17862.24) | 44818.82 (37100.44) | 6-month visit – baseline | 1618.81 (-16755.98, 19993.6) | 0.192 | 23607.76 (-4718.26, 51933.79) | 0.839 | 25226.58 (5911, 44542.15) | 0.269 | 0.177 |
| 1-year visit | 36654.77 (18169.49) | 64017 (34778.38) | 38129.59 (16060.93) | 1-year visit – 6-month visit | 30053.05 (10854.07, 49252.02) | 0.637 | -35076.76 (-61312.52, -8841.01) | 0.714 | -5023.72 (-24441.99, 14394.55) | 0.155 | 0.649 |
|  |  |  |  | 1-year visit – baseline | 31671.86 (10169.56, 53174.15) | 0.033 | -11469 (-42335.82, 19397.82) | 0.039 | 20202.86 (7690.22, 32715.5) | 0.331 | 0.14 |
| **D-Fructose** |  |  |  |  |  |  |  |  |  |  |  |
| Baseline | 25307 (9500.22) | 25402.65 (23069.44) | 17359.41 (8663.55) |  |  |  |  |  |  |  |  |
| 6-month visit | 22048.26 (10967.9) | 21453.65 (9955.1) | 24294.12 (15187.86) | 6-month visit – baseline | -690.26 (-10243.61, 8863.1) | 0.224 | 10883.71 (-2139.4, 23906.82) | 0.878 | 10193.45 (1178.6, 19208.29) | 0.469 | 0.23 |
| 1-year visit | 20983.26 (9473.64) | 33333.29 (14880.8) | 21766.71 (8678.94) | 1-year visit – 6-month visit | 12944.65 (3664.31, 22224.98) | 0.686 | -14407.06 (-25654.38, -3159.74) | 0.606 | -1462.41 (-10568.71, 7643.89) | 0.139 | 0.708 |
|  |  |  |  | 1-year visit – baseline | 12254.39 (1751.47, 22757.3) | 0.098 | -3523.35 (-18047.25, 11000.55) | 0.045 | 8731.04 (1765.11, 15696.96) | 0.326 | 0.231 |
| **Gluconolactone** |  |  |  |  |  |  |  |  |  |  |  |
| Baseline | 24463.58 (7561.63) | 24493.47 (7611.59) | 29192.12 (5993.59) |  |  |  |  |  |  |  |  |
| 6-month visit | 27789.61 (8753.36) | 20894.47 (6304.85) | 26704.41 (6417.24) | 6-month visit – baseline | -6925.03 (-12618.63, -1231.44) | 0.405 | 1111.29 (-5874.57, 8097.16) | 0.01 | -5813.74 (-11552.87, -74.61) | 0.053 | 0.513 |
| 1-year visit | 26792.16 (10295.89) | 22530.53 (6319.22) | 25497.24 (6742.09) | 1-year visit – 6-month visit | 2633.51 (-3735.64, 9002.67) | 0.553 | -2843.24 (-7837.44, 2150.97) | 0.038 | -209.72 (-6698.12, 6278.67) | 0.004 | 0.361 |
|  |  |  |  | 1-year visit – baseline | -4291.52 (-11476.19, 2893.14) | 0.379 | -1731.94 (-8182.43, 4718.55) | 0.049 | -6023.46 (-13101.95, 1055.02) | 0.195 | 0.463 |
| **D-Mannose** |  |  |  |  |  |  |  |  |  |  |  |
| Baseline | 544435 (190796.42) | 502103.47 (191855.85) | 613030.18 (196825.35) |  |  |  |  |  |  |  |  |
| 6-month visit | 578603.26 (199434.96) | 502743.53 (190075.74) | 577050.65 (124807.49) | 6-month visit – baseline | -33528.2 (-188493.69, 121437.29) | 0.49 | -36619.59 (-197822.52, 124583.34) | 0.029 | -70147.79 (-219165.48, 78869.9) | 0.065 | 0.726 |
| 1-year visit | 562511.87 (254138.12) | 560991.65 (156131.6) | 534808.82 (193191.26) | 1-year visit – 6-month visit | 74339.5 (-78214.8, 226893.81) | 0.571 | -100489.94 (-267285.46, 66305.58) | 0.322 | -26150.44 (-189593.44, 137292.57) | 0.077 | 0.426 |
|  |  |  |  | 1-year visit – baseline | 40811.31 (-127562.26, 209184.87) | 0.881 | -137109.53 (-322147.86, 47928.8) | 0.285 | -96298.22 (-274655.77, 82059.32) | 0.196 | 0.955 |
| **D-Galactose** |  |  |  |  |  |  |  |  |  |  |  |
| Baseline | 205321.42 (115341.26) | 245547.65 (152408.37) | 241235.06 (178972.56) |  |  |  |  |  |  |  |  |
| 6-month visit | 203663.13 (129182.94) | 183472.76 (113258.02) | 239876.12 (123537.8) | 6-month visit – baseline | -60416.59 (-171950.28, 51117.1) | 0.168 | 60715.94 (-66438.24, 187870.12) | 0.844 | 299.35 (-110588.33, 111187.02) | 0.272 | 0.124 |
| 1-year visit | 209563.9 (123119.92) | 226888.12 (123817.34) | 241508.29 (151233.48) | 1-year visit – 6-month visit | 37514.58 (-68575.59, 143604.75) | 0.313 | -41783.18 (-168026.43, 84460.07) | 0.343 | -4268.6 (-106963.36, 98426.16) | 0.96 | 0.266 |
|  |  |  |  | 1-year visit – baseline | -22902.01 (-123126.08, 77322.06) | 0.266 | 18932.76 (-118844.29, 156709.82) | 0.94 | -3969.25 (-112509.97, 104571.47) | 0.32 | 0.252 |
| **Glucosamine** |  |  |  |  |  |  |  |  |  |  |  |
| Baseline | 2537.61 (1697.99) | 2190.29 (1733.37) | 1524.94 (1157.83) |  |  |  |  |  |  |  |  |
| 6-month visit | 2651.77 (1826.08) | 2256.12 (1754) | 2011.06 (1645.64) | 6-month visit – baseline | -48.34 (-1629.89, 1533.21) | 0.022 | 420.29 (-1325.31, 2165.9) | 0.162 | 371.96 (-1080.33, 1824.24) | 0.378 | 0.029 |
| 1-year visit | 2871.48 (2236.62) | 1778.94 (1291.56) | 1725.41 (1375) | 1-year visit – 6-month visit | -696.89 (-2300.77, 907) | 0.021 | 191.53 (-1214.49, 1597.55) | 0.64 | -505.36 (-2116.17, 1105.46) | 0.047 | 0.016 |
|  |  |  |  | 1-year visit – baseline | -745.22 (-2291.32, 800.87) | 0.012 | 611.82 (-671.04, 1894.69) | 0.304 | -133.4 (-1514.08, 1247.28) | 0.066 | 0.006 |
| **D-Glucose** |  |  |  |  |  |  |  |  |  |  |  |
| Baseline | 200826.58 (133703.54) | 208976.59 (155901.29) | 187509.82 (126247.67) |  |  |  |  |  |  |  |  |
| 6-month visit | 185870.58 (130361.83) | 219457.53 (98048.69) | 189222.24 (132387.66) | 6-month visit – baseline | 25436.94 (-81365.52, 132239.4) | 0.828 | -8768.53 (-135598.45, 118061.39) | 0.316 | 16668.41 (-92918.87, 126255.69) | 0.399 | 0.98 |
| 1-year visit | 218080.65 (130897.1) | 202408.88 (109741.26) | 234155.65 (142741.63) | 1-year visit – 6-month visit | -49258.71 (-153044.53, 54527.1) | 0.928 | 61982.06 (-47716.56, 171680.68) | 0.819 | 12723.35 (-98378.82, 123825.52) | 0.538 | 0.819 |
|  |  |  |  | 1-year visit – baseline | -23821.77 (-127898.09, 80254.55) | 0.92 | 53213.53 (-57560.13, 163987.18) | 0.764 | 29391.76 (-81679.11, 140462.63) | 0.856 | 0.966 |
| **L-Histidine** |  |  |  |  |  |  |  |  |  |  |  |
| Baseline | 14571.65 (4956.57) | 22228.18 (11205.23) | 23434.12 (8643.34) |  |  |  |  |  |  |  |  |
| 6-month visit | 18173.13 (7375.88) | 16153.71 (8814.12) | 20700.94 (8390.78) | 6-month visit – baseline | -9675.95 (-16379.38, -2972.53) | 0.002 | 3341.29 (-5343.03, 12025.62) | 0.241 | -6334.66 (-11699.12, -970.2) | 0.244 | 0.003 |
| 1-year visit | 23658.58 (8203.52) | 22586.65 (8755.61) | 15498.41 (5982.81) | 1-year visit – 6-month visit | 947.49 (-6569.39, 8464.37) | 0.123 | -11635.47 (-19624.4, -3646.54) | 0.591 | -10687.98 (-17494.39, -3881.57) | 0.343 | 0.103 |
|  |  |  |  | 1-year visit – baseline | -8728.46 (-15595.45, -1861.48) | 0.935 | -8294.18 (-16440.57, -147.79) | 0.327 | -17022.64 (-22957.55, -11087.73) | 0.26 | 0.868 |
| **L-Lysine** |  |  |  |  |  |  |  |  |  |  |  |
| Baseline | 73781.61 (20666.48) | 85700.65 (24755.67) | 82713.35 (27216.39) |  |  |  |  |  |  |  |  |
| 6-month visit | 77874.55 (15389.48) | 78157.41 (24880.41) | 76502.24 (21743.35) | 6-month visit – baseline | -11636.17 (-26019.44, 2747.1) | 0.547 | 1332.12 (-20349.6, 23013.83) | 0.764 | -10304.05 (-24825.82, 4217.71) | 0.38 | 0.517 |
| 1-year visit | 78967.03 (19313.89) | 88069.29 (22640.6) | 74347.88 (21991.88) | 1-year visit – 6-month visit | 8819.4 (-6542.78, 24181.58) | 0.508 | -12066.24 (-34172.85, 10040.37) | 0.263 | -3246.84 (-19986.17, 13492.5) | 0.486 | 0.55 |
|  |  |  |  | 1-year visit – baseline | -2816.77 (-19133.19, 13499.65) | 0.77 | -10734.12 (-32874.68, 11406.45) | 0.331 | -13550.89 (-30948.98, 3847.2) | 0.079 | 0.684 |
| **Mannitol** |  |  |  |  |  |  |  |  |  |  |  |
| Baseline | 17345.81 (13874.25) | 12312.06 (7317.05) | 20967.82 (33313.42) |  |  |  |  |  |  |  |  |
| 6-month visit | 16002.71 (9702.84) | 9594.94 (4588.17) | 10147.47 (7799.78) | 6-month visit – baseline | -1374.02 (-8433.79, 5685.75) | 0.761 | -8103.24 (-25603.05, 9396.58) | 0.314 | -9477.26 (-23570.21, 4615.69) | 0.02 | 0.6 |
| 1-year visit | 14120.68 (6928.93) | 13271.24 (10473.58) | 10124 (7949.74) | 1-year visit – 6-month visit | 5558.33 (-743.87, 11860.52) | 0.022 | -3699.76 (-10354.66, 2955.13) | 0.927 | 1858.56 (-3452.48, 7169.6) | 0.031 | 0.007 |
|  |  |  |  | 1-year visit – baseline | 4184.31 (-3826.76, 12195.37) | 0.969 | -11803 (-28457.99, 4851.99) | 0.544 | -7618.69 (-21050.01, 5812.62) | 0.188 | 0.899 |
| **L-Tyrosine** |  |  |  |  |  |  |  |  |  |  |  |
| Baseline | 99358.03 (42153.37) | 99035.65 (38198.78) | 112203.06 (45864.78) |  |  |  |  |  |  |  |  |
| 6-month visit | 89307.81 (27713.36) | 74963.65 (29582.73) | 88747.12 (30887.31) | 6-month visit – baseline | -14021.77 (-36980.22, 8936.67) | 0.493 | 616.06 (-34051.26, 35283.38) | 0.136 | -13405.72 (-40106.36, 13294.93) | 0.503 | 0.573 |
| 1-year visit | 97836.74 (26382.06) | 100961.12 (43610.22) | 88379.12 (46978.85) | 1-year visit – 6-month visit | 17468.54 (-5750.54, 40687.61) | 0.447 | -26365.47 (-63047.34, 10316.4) | 0.68 | -8896.94 (-33773.06, 15979.19) | 0.37 | 0.381 |
|  |  |  |  | 1-year visit – baseline | 3446.76 (-24357.51, 31251.03) | 0.886 | -25749.41 (-66313.71, 14814.88) | 0.773 | -22302.65 (-52995.62, 8390.31) | 0.878 | 0.906 |
| **Gluconic acid** |  |  |  |  |  |  |  |  |  |  |  |
| Baseline | 2853.1 (963.12) | 2502.53 (943.76) | 2661.76 (1126.3) |  |  |  |  |  |  |  |  |
| 6-month visit | 2132.16 (564.47) | 2081.94 (566.57) | 2202.29 (826.72) | 6-month visit – baseline | 300.35 (-296.84, 897.53) | 0.652 | -38.88 (-846.61, 768.84) | 0.404 | 261.46 (-437.83, 960.76) | 0.142 | 0.562 |
| 1-year visit | 2507.77 (1001.73) | 3041.65 (1362.32) | 2096.71 (1055.53) | 1-year visit – 6-month visit | 584.09 (-55.62, 1223.81) | 0.345 | -1065.29 (-1747.59, -383) | 0.344 | -481.2 (-1015.18, 52.78) | 0.885 | 0.344 |
|  |  |  |  | 1-year visit – baseline | 884.44 (246.45, 1522.43) | 0.136 | -1104.18 (-1929.16, -279.19) | 0.482 | -219.74 (-907.3, 467.83) | 0.518 | 0.152 |
| **Palmitoleic acid** |  |  |  |  |  |  |  |  |  |  |  |
| Baseline | 3409.13 (3503.1) | 4597.47 (5130.87) | 7766.06 (8510.6) |  |  |  |  |  |  |  |  |
| 6-month visit | 5499.26 (6656.82) | 2691.24 (2590.12) | 6059.65 (8980.84) | 6-month visit – baseline | -3996.36 (-7437.1, -555.63) | 0.066 | 199.82 (-5849.63, 6249.28) | 0.198 | -3796.54 (-8503.58, 910.5) | 0.756 | 0.063 |
| 1-year visit | 5922.23 (6742.52) | 3276.18 (3421.66) | 5476.88 (4527.44) | 1-year visit – 6-month visit | 161.97 (-4442.97, 4766.92) | 0.741 | -1167.71 (-6201.02, 3865.61) | 0.112 | -1005.73 (-6447.67, 4436.2) | 0.205 | 0.898 |
|  |  |  |  | 1-year visit – baseline | -3834.39 (-7962.59, 293.81) | 0.069 | -967.88 (-6429.25, 4493.48) | 0.091 | -4802.27 (-9830.2, 225.65) | 0.667 | 0.102 |
| **Palmitic acid** |  |  |  |  |  |  |  |  |  |  |  |
| Baseline | 169094.03 (68874.08) | 188233.76 (98018.19) | 234120.18 (116792.92) |  |  |  |  |  |  |  |  |
| 6-month visit | 207113.71 (119940.62) | 155588.24 (70701.92) | 202402.47 (147132.67) | 6-month visit – baseline | -70665.21 (-138771.26, -2559.15) | 0.115 | 927.82 (-94493.96, 96349.6) | 0.286 | -69737.38 (-145571.05, 6096.28) | 0.76 | 0.151 |
| 1-year visit | 210848.9 (110337.87) | 180006 (93238.44) | 208132.94 (74705.26) | 1-year visit – 6-month visit | 20682.57 (-54233.78, 95598.92) | 0.732 | -18687.29 (-112592.25, 75217.66) | 0.247 | 1995.28 (-81852.48, 85843.03) | 0.389 | 0.956 |
|  |  |  |  | 1-year visit – baseline | -49982.64 (-123335.59, 23370.31) | 0.044 | -17759.47 (-106390.8, 70871.86) | 0.169 | -67742.11 (-149355.23, 13871.01) | 0.969 | 0.103 |
| **Myoinositol** |  |  |  |  |  |  |  |  |  |  |  |
| Baseline | 33182.87 (10998.94) | 34481.41 (11213.64) | 33481.29 (9006.11) |  |  |  |  |  |  |  |  |
| 6-month visit | 31593 (6736.71) | 27811.18 (12283.3) | 30798.18 (8626.88) | 6-month visit – baseline | -5080.36 (-12779.04, 2618.31) | 0.846 | 3987.12 (-4698.3, 12672.54) | 0.536 | -1093.25 (-7665.18, 5478.69) | 0.172 | 0.669 |
| 1-year visit | 35274.23 (12023) | 37590.12 (13514.92) | 28069.47 (7808.06) | 1-year visit – 6-month visit | 6097.72 (-1413.02, 13608.45) | 0.095 | -12507.65 (-21515.96, -3499.33) | 0.434 | -6409.93 (-13806.24, 986.38) | 0.262 | 0.086 |
|  |  |  |  | 1-year visit – baseline | 1017.35 (-7653.73, 9688.43) | 0.128 | -8520.53 (-18455.6, 1414.54) | 0.127 | -7503.18 (-16200.62, 1194.27) | 0.886 | 0.145 |
| **Uric acid** |  |  |  |  |  |  |  |  |  |  |  |
| Baseline | 60884.35 (23082.42) | 49606.06 (29723.64) | 62645.18 (25670.51) |  |  |  |  |  |  |  |  |
| 6-month visit | 69103.42 (18039.37) | 66529.71 (22945.86) | 61829.47 (33466.82) | 6-month visit – baseline | 8704.58 (-7149.16, 24558.33) | 0.695 | -17739.35 (-36869.07, 1390.37) | 0.379 | -9034.77 (-26302.29, 8232.75) | 0.318 | 0.582 |
| 1-year visit | 57693.39 (21837.56) | 66069.35 (19999.53) | 71486.94 (18248.71) | 1-year visit – 6-month visit | 10949.68 (-4004.84, 25904.2) | 0.507 | 10117.82 (-13624.54, 33860.18) | 0.573 | 21067.5 (3225.87, 38909.14) | 0.612 | 0.567 |
|  |  |  |  | 1-year visit – baseline | 19654.26 (3123.14, 36185.38) | 0.19 | -7621.53 (-30721.43, 15478.37) | 0.057 | 12032.73 (-1945.69, 26011.16) | 0.674 | 0.265 |
| **Heptadecanoic acid** |  |  |  |  |  |  |  |  |  |  |  |
| Baseline | 270889.19 (32131.32) | 260366.82 (26524.69) | 259030.76 (19198.99) |  |  |  |  |  |  |  |  |
| 6-month visit | 247697.16 (31201.06) | 253445.88 (13966.53) | 259369.59 (21497.36) | 6-month visit – baseline | 16271.09 (-6932.59, 39474.77) | 0.796 | 7259.76 (-13318.41, 27837.94) | 0.343 | 23530.86 (-25.24, 47086.95) | 0.7 | 0.893 |
| 1-year visit | 266171.71 (44043.41) | 281137.94 (29890.26) | 256325 (25237.45) | 1-year visit – 6-month visit | 9217.51 (-16830.59, 35265.61) | 0.688 | -30736.65 (-54924.07, -6549.23) | 0.245 | -21519.14 (-47614.38, 4576.1) | 0.139 | 0.673 |
|  |  |  |  | 1-year visit – baseline | 25488.6 (-865.42, 51842.62) | 0.211 | -23476.88 (-41790.11, -5163.66) | 0.266 | 2011.72 (-24834.22, 28857.66) | 0.79 | 0.184 |
| **Indolelactic acid** |  |  |  |  |  |  |  |  |  |  |  |
| Baseline | 1362.13 (666.53) | 1250.47 (717.68) | 1522.06 (536.92) |  |  |  |  |  |  |  |  |
| 6-month visit | 1497.32 (879.5) | 1150.29 (570.55) | 1482.18 (673.97) | 6-month visit – baseline | -235.37 (-674.35, 203.61) | 0.576 | 60.29 (-558.54, 679.13) | 0.135 | -175.08 (-604.7, 254.55) | 0.424 | 0.64 |
| 1-year visit | 1559.26 (774.02) | 1338.59 (509.09) | 1270.59 (603.44) | 1-year visit – 6-month visit | 126.36 (-328.08, 580.8) | 0.581 | -399.88 (-985.7, 185.93) | 0.445 | -273.52 (-721.07, 174.03) | 0.25 | 0.421 |
|  |  |  |  | 1-year visit – baseline | -109.01 (-532.07, 314.05) | 0.842 | -339.59 (-911.99, 232.81) | 0.736 | -448.6 (-868.41, -28.79) | 0.472 | 0.759 |
| **5-Hydroxydopamine** |  |  |  |  |  |  |  |  |  |  |  |
| Baseline | 908.48 (619.39) | 925.82 (401.03) | 1018.88 (717.7) |  |  |  |  |  |  |  |  |
| 6-month visit | 827 (519.77) | 548.65 (243.11) | 845.71 (440.13) | 6-month visit – baseline | -295.69 (-650.92, 59.54) | 0.671 | 204 (-220.56, 628.56) | 0.138 | -91.69 (-523.61, 340.22) | 0.577 | 0.696 |
| 1-year visit | 806.39 (336.47) | 1058.88 (730.59) | 664.88 (537.99) | 1-year visit – 6-month visit | 530.85 (127.98, 933.72) | 0.581 | -691.06 (-1194.41, -187.71) | 0.855 | -160.21 (-596.13, 275.71) | 0.852 | 0.605 |
|  |  |  |  | 1-year visit – baseline | 235.16 (-203.48, 673.79) | 0.937 | -487.06 (-1064.46, 90.34) | 0.291 | -251.9 (-744.04, 240.24) | 0.146 | 0.902 |
| **L-Kynurenine** |  |  |  |  |  |  |  |  |  |  |  |
| Baseline | 247.1 (66.66) | 262.71 (89.81) | 308.59 (67.51) |  |  |  |  |  |  |  |  |
| 6-month visit | 243 (72.86) | 223.65 (62.13) | 248.76 (83.46) | 6-month visit – baseline | -34.96 (-85.02, 15.09) | 0.066 | -20.76 (-88.39, 46.86) | 0.127 | -55.73 (-111.07, -0.38) | 0.858 | 0.082 |
| 1-year visit | 271.52 (74.42) | 268.47 (89.03) | 248.82 (122.09) | 1-year visit – 6-month visit | 16.31 (-36.02, 68.64) | 0.561 | -44.76 (-137.08, 47.55) | 0.631 | -28.46 (-97.81, 40.89) | 0.19 | 0.427 |
|  |  |  |  | 1-year visit – baseline | -18.65 (-73.92, 36.61) | 0.36 | -65.53 (-156.43, 25.37) | 0.451 | -84.18 (-148.73, -19.64) | 0.868 | 0.408 |
| **Linoleic acid** |  |  |  |  |  |  |  |  |  |  |  |
| Baseline | 24076.42 (13123.67) | 29034.29 (19108.95) | 33379.76 (16968.79) |  |  |  |  |  |  |  |  |
| 6-month visit | 28012.97 (19034.46) | 20049.76 (9211.23) | 34097.35 (27586.57) | 6-month visit – baseline | -12921.08 (-24671.58, -1170.58) | 0.043 | 9702.12 (-7276.06, 26680.3) | 0.212 | -3218.96 (-16456.53, 10018.61) | 0.891 | 0.051 |
| 1-year visit | 31755.55 (22857.09) | 20995.24 (11982.84) | 33038.47 (14615.84) | 1-year visit – 6-month visit | -2797.11 (-17499.41, 11905.19) | 0.304 | -2004.35 (-18529.01, 14520.3) | 0.012 | -4801.46 (-22031.11, 12428.18) | 0.108 | 0.439 |
|  |  |  |  | 1-year visit – baseline | -15718.19 (-29936.11, -1500.27) | 0.119 | 7697.76 (-6518.55, 21914.08) | 0.06 | -8020.42 (-22828.92, 6788.08) | 0.455 | 0.22 |
| **L-Tryptophan** |  |  |  |  |  |  |  |  |  |  |  |
| Baseline | 77412.77 (22888.44) | 79235 (27701.74) | 88161.47 (34605.89) |  |  |  |  |  |  |  |  |
| 6-month visit | 81060.45 (27583.87) | 72846.59 (23956.18) | 83750.18 (29912.08) | 6-month visit – baseline | -10036.09 (-28581.73, 8509.55) | 0.261 | 1977.12 (-26189.06, 30143.29) | 0.263 | -8058.97 (-30885.38, 14767.44) | 0.515 | 0.329 |
| 1-year visit | 87973.29 (25106.83) | 97184.65 (25997.06) | 74524.29 (31817) | 1-year visit – 6-month visit | 17425.22 (46.59, 34803.85) | 0.428 | -33563.94 (-60433.11, -6694.77) | 0.614 | -16138.72 (-36673.35, 4395.9) | 0.541 | 0.332 |
|  |  |  |  | 1-year visit – baseline | 7389.13 (-11398.95, 26177.22) | 0.823 | -31586.82 (-58614.9, -4558.75) | 0.573 | -24197.69 (-44668.46, -3726.92) | 0.682 | 0.804 |
| **Alpha Linolenic acid** |  |  |  |  |  |  |  |  |  |  |  |
| Baseline | 65867.45 (64308.39) | 81621.59 (93872.03) | 84971.71 (72280.42) |  |  |  |  |  |  |  |  |
| 6-month visit | 101043.19 (112527.18) | 54159.06 (55832.27) | 88843.82 (132293.09) | 6-month visit – baseline | -62638.27 (-135003.64, 9727.09) | 0.604 | 31334.65 (-50293.72, 112963.01) | 0.851 | -31303.62 (-107118.95, 44511.7) | 0.908 | 0.579 |
| 1-year visit | 77029.81 (75701.5) | 71120.18 (78237.95) | 73373.76 (68398.2) | 1-year visit – 6-month visit | 40974.5 (-23855.78, 105804.79) | 0.774 | -32431.18 (-116880.5, 52018.14) | 0.969 | 8543.33 (-68262.19, 85348.85) | 0.987 | 0.845 |
|  |  |  |  | 1-year visit – baseline | -21663.77 (-86457.75, 43130.21) | 0.353 | -1096.53 (-85568.66, 83375.6) | 0.853 | -22760.3 (-88475.93, 42955.34) | 0.469 | 0.402 |
| **Petroselinic acid** |  |  |  |  |  |  |  |  |  |  |  |
| Baseline | 76740.94 (55621.8) | 93577.29 (73944.02) | 136484.88 (81014.87) |  |  |  |  |  |  |  |  |
| 6-month visit | 110350.19 (87681.75) | 71542.53 (43430.73) | 109533.29 (96880.2) | 6-month visit – baseline | -55644.02 (-105417.11, -5870.94) | 0.054 | -4916.82 (-71622.63, 61788.98) | 0.129 | -60560.85 (-113189.59, -7932.1) | 0.921 | 0.063 |
| 1-year visit | 105980.65 (79808.25) | 72732.94 (57796.09) | 126769.29 (71513.41) | 1-year visit – 6-month visit | 5559.96 (-48578.66, 59698.58) | 0.36 | 16045.59 (-49195.77, 81286.95) | 0.03 | 21605.55 (-39480.2, 82691.3) | 0.281 | 0.508 |
|  |  |  |  | 1-year visit – baseline | -50084.06 (-102933.24, 2765.11) | 0.005 | 11128.76 (-57840.61, 80098.13) | 0.007 | -38955.3 (-101612.77, 23702.18) | 0.798 | 0.014 |
| **Oleic acid** |  |  |  |  |  |  |  |  |  |  |  |
| Baseline | 4706.42 (2733.38) | 5834.82 (4031.59) | 7619.94 (4774) |  |  |  |  |  |  |  |  |
| 6-month visit | 5712.94 (4057.72) | 4334.29 (2304.57) | 5703.82 (4730.99) | 6-month visit – baseline | -2507.05 (-5255.23, 241.14) | 0.076 | -415.59 (-3970.08, 3138.91) | 0.312 | -2922.63 (-5687.85, -157.42) | 0.95 | 0.073 |
| 1-year visit | 5547.42 (4222.64) | 3914.94 (3610.62) | 6615.88 (4938.08) | 1-year visit – 6-month visit | -253.84 (-3316.47, 2808.8) | 0.387 | 1331.41 (-2605.85, 5268.68) | 0.09 | 1077.57 (-2434.81, 4589.96) | 0.232 | 0.532 |
|  |  |  |  | 1-year visit – baseline | -2760.88 (-5863.23, 341.46) | 0.013 | 915.82 (-3163.56, 4995.21) | 0.075 | -1845.06 (-5405.73, 1715.62) | 0.79 | 0.025 |
| **Stearic acid** |  |  |  |  |  |  |  |  |  |  |  |
| Baseline | 82276.74 (23800.79) | 86973.71 (32253.55) | 106134.12 (32390.39) |  |  |  |  |  |  |  |  |
| 6-month visit | 95028.84 (34913.47) | 75753.71 (23705.96) | 92015.18 (47451.35) | 6-month visit – baseline | -23972.1 (-45389.42, -2554.78) | 0.104 | -2898.94 (-32022.33, 26224.45) | 0.167 | -26871.04 (-49930.33, -3811.74) | 0.548 | 0.147 |
| 1-year visit | 90440.45 (43159.47) | 81420.06 (33134.12) | 96315.53 (23621.32) | 1-year visit – 6-month visit | 10254.74 (-15788.96, 36298.44) | 0.485 | -1366 (-32551.52, 29819.52) | 0.162 | 8888.74 (-19444.49, 37221.97) | 0.37 | 0.697 |
|  |  |  |  | 1-year visit – baseline | -13717.36 (-39907.57, 12472.86) | 0.011 | -4264.94 (-29681.92, 21152.04) | 0.058 | -17982.3 (-45595.45, 9630.85) | 0.952 | 0.039 |
| **L-Cystine** |  |  |  |  |  |  |  |  |  |  |  |
| Baseline | 3921.84 (1977.49) | 3342 (2521.23) | 4334 (1867.97) |  |  |  |  |  |  |  |  |
| 6-month visit | 3544 (1524.02) | 4047.88 (2626.5) | 4728.82 (2575.98) | 6-month visit – baseline | 1083.72 (-235.16, 2402.6) | 0.086 | -311.06 (-2122.78, 1500.66) | 0.119 | 772.66 (-619.95, 2165.27) | 0.707 | 0.231 |
| 1-year visit | 4817.29 (2147.01) | 5049.06 (1788.04) | 3407.12 (2317.97) | 1-year visit – 6-month visit | -272.11 (-1684.37, 1140.14) | 0.736 | -2322.88 (-4366.56, -279.21) | 0.721 | -2595 (-4440.33, -749.66) | 0.67 | 0.729 |
|  |  |  |  | 1-year visit – baseline | 811.61 (-898.05, 2521.26) | 0.28 | -2633.94 (-4494.09, -773.8) | 0.964 | -1822.33 (-3555.71, -88.96) | 0.659 | 0.243 |
| **Nonadecanoic acid** |  |  |  |  |  |  |  |  |  |  |  |
| Baseline | 472.13 (103.35) | 438.41 (106.03) | 489.82 (99.51) |  |  |  |  |  |  |  |  |
| 6-month visit | 445.61 (103.04) | 463.41 (93.46) | 488.24 (101.07) | 6-month visit – baseline | 51.52 (-33.12, 136.16) | 0.094 | -26.59 (-118.33, 65.15) | 0.218 | 24.93 (-66.42, 116.28) | 0.857 | 0.156 |
| 1-year visit | 522.32 (207.52) | 529.35 (123.47) | 468.18 (127.81) | 1-year visit – 6-month visit | -10.77 (-130.01, 108.47) | 0.828 | -86 (-201.35, 29.35) | 0.581 | -96.77 (-225.67, 32.13) | 0.353 | 0.878 |
|  |  |  |  | 1-year visit – baseline | 40.75 (-82.65, 164.14) | 0.826 | -112.59 (-192.51, -32.66) | 0.926 | -71.84 (-195.08, 51.4) | 0.959 | 0.686 |
| **Arachidonic acid** |  |  |  |  |  |  |  |  |  |  |  |
| Baseline | 2642.23 (771.35) | 3519.65 (1702.07) | 3225.24 (1864.9) |  |  |  |  |  |  |  |  |
| 6-month visit | 2744.97 (820.18) | 3021.24 (1379.95) | 3389.59 (1690.98) | 6-month visit – baseline | -601.15 (-1339.52, 137.22) | 0.029 | 662.76 (-385.55, 1711.08) | 0.914 | 61.61 (-611.37, 734.59) | 0.181 | 0.058 |
| 1-year visit | 2334.42 (892.51) | 3098.82 (1232.41) | 3387 (1032.39) | 1-year visit – 6-month visit | 488.14 (-279.21, 1255.48) | 0.001 | -80.18 (-1147.63, 987.27) | 0.473 | 407.96 (-376.33, 1192.25) | 0.269 | 0.003 |
|  |  |  |  | 1-year visit – baseline | -113.02 (-927.14, 701.11) | 0.002 | 582.59 (-583.14, 1748.32) | 0.841 | 469.57 (-354.98, 1294.12) | 0.042 | 0.005 |
| **N Acetylserotonin** |  |  |  |  |  |  |  |  |  |  |  |
| Baseline | 744369.45 (200829.4) | 738704.18 (170212.8) | 678468.18 (193855.92) |  |  |  |  |  |  |  |  |
| 6-month visit | 750236.52 (220424.88) | 739438.06 (182010.85) | 640026.41 (194467.53) | 6-month visit – baseline | -5133.18 (-186143.02, 175876.66) | 0.053 | -39175.65 (-212970.56, 134619.26) | 0.12 | -44308.83 (-239868.84, 151251.18) | 0.871 | 0.05 |
| 1-year visit | 684046.61 (268381.16) | 768325.47 (183918.58) | 660682.18 (251876.12) | 1-year visit – 6-month visit | 95077.31 (-93109.05, 283263.68) | 0.187 | -8231.65 (-233096.89, 216633.6) | 0.073 | 86845.67 (-125454.34, 299145.68) | 0.782 | 0.182 |
|  |  |  |  | 1-year visit – baseline | 89944.13 (-86790.54, 266678.8) | 0.43 | -47407.29 (-242153.29, 147338.7) | 0.134 | 42536.84 (-141838.63, 226912.31) | 0.413 | 0.467 |
| **Docosahexaenoic acid** |  |  |  |  |  |  |  |  |  |  |  |
| Baseline | 988.97 (445.49) | 1162.71 (609.63) | 1292.18 (773.44) |  |  |  |  |  |  |  |  |
| 6-month visit | 1080.74 (459.06) | 861 (411.22) | 1071 (583.26) | 6-month visit – baseline | -393.48 (-715, -71.96) | 0.134 | 80.53 (-383.17, 544.22) | 0.54 | -312.95 (-711.5, 85.6) | 0.959 | 0.187 |
| 1-year visit | 1054.9 (616.31) | 1120.18 (478.58) | 976.53 (213.67) | 1-year visit – 6-month visit | 285.02 (-74.53, 644.56) | 0.933 | -353.65 (-740.04, 32.75) | 0.756 | -68.63 (-436.57, 299.31) | 0.509 | 0.742 |
|  |  |  |  | 1-year visit – baseline | -108.46 (-463.1, 246.17) | 0.241 | -273.12 (-750.23, 203.99) | 0.874 | -381.58 (-789.24, 26.08) | 0.466 | 0.3 |
| **Sucrose** |  |  |  |  |  |  |  |  |  |  |  |
| Baseline | 650.32 (385.35) | 842.65 (718.78) | 894.76 (1417.18) |  |  |  |  |  |  |  |  |
| 6-month visit | 699.65 (494.73) | 735.35 (494.01) | 625.71 (429.17) | 6-month visit – baseline | -156.62 (-552.02, 238.78) | 0.48 | -161.76 (-1034.47, 710.94) | 0.856 | -318.38 (-925.18, 288.41) | 0.216 | 0.459 |
| 1-year visit | 660.84 (341.65) | 928.18 (798.32) | 590.29 (286.18) | 1-year visit – 6-month visit | 231.63 (-183.87, 647.13) | 0.48 | -228.24 (-773.43, 316.96) | 0.23 | 3.39 (-329.65, 336.44) | 0.188 | 0.574 |
|  |  |  |  | 1-year visit – baseline | 75.01 (-336.9, 486.93) | 0.499 | -390 (-1259.46, 479.46) | 0.621 | -314.99 (-892.43, 262.46) | 0.184 | 0.538 |
| **Adenosine** |  |  |  |  |  |  |  |  |  |  |  |
| Baseline | 3727.58 (1899.36) | 3152.47 (2196.6) | 3688.94 (1608.58) |  |  |  |  |  |  |  |  |
| 6-month visit | 2172.61 (1051.01) | 2119.29 (523.03) | 2387.71 (1524) | 6-month visit – baseline | 521.79 (-861.12, 1904.7) | 0.905 | -268.06 (-1741.28, 1205.17) | 0.134 | 253.73 (-1101.2, 1608.66) | 0.172 | 0.858 |
| 1-year visit | 4067 (1919.22) | 3395.76 (1531.04) | 1984.88 (863.31) | 1-year visit – 6-month visit | -617.92 (-1859.71, 623.88) | 0.006 | -1679.29 (-2895.44, -463.15) | 0.068 | -2297.21 (-3554.67, -1039.75) | 0.245 | 0.005 |
|  |  |  |  | 1-year visit – baseline | -96.13 (-1692.18, 1499.93) | 0.003 | -1947.35 (-3667.95, -226.76) | 0.48 | -2043.48 (-3413.61, -673.35) | 0.03 | 0.002 |
| **Alpha-Lactose** |  |  |  |  |  |  |  |  |  |  |  |
| Baseline | 370.84 (575.76) | 372.41 (611.38) | 436.53 (552.08) |  |  |  |  |  |  |  |  |
| 6-month visit | 481.13 (722.89) | 377.29 (633.83) | 194.94 (128.77) | 6-month visit – baseline | -105.41 (-617.84, 407.02) | 0.368 | -246.47 (-805.08, 312.14) | 0.61 | -351.88 (-785.17, 81.41) | 0.701 | 0.377 |
| 1-year visit | 507.74 (686.28) | 839.41 (877) | 346.06 (469.71) | 1-year visit – 6-month visit | 435.5 (-226.4, 1097.41) | 0.095 | -311 (-901.36, 279.36) | 0.014 | 124.5 (-426.6, 675.61) | 0.543 | 0.195 |
|  |  |  |  | 1-year visit – baseline | 330.1 (-252.52, 912.72) | 0.692 | -557.47 (-1100.97, -13.97) | 0.391 | -227.37 (-764.42, 309.68) | 0.61 | 0.715 |
| **Cellobiose** |  |  |  |  |  |  |  |  |  |  |  |
| Baseline | 1093.84 (1236.49) | 502 (379.16) | 471.53 (246.48) |  |  |  |  |  |  |  |  |
| 6-month visit | 685.23 (469.15) | 520.35 (335.02) | 532.76 (261.37) | 6-month visit – baseline | 426.97 (-267.92, 1121.85) | 0.018 | 42.88 (-267.46, 353.22) | 0.644 | 469.85 (-220.82, 1160.52) | 0.013 | 0.004 |
| 1-year visit | 500.84 (206.89) | 694.29 (676.47) | 443.82 (285.6) | 1-year visit – 6-month visit | 358.33 (-17.72, 734.37) | 0.151 | -262.88 (-676.67, 150.91) | 0.339 | 95.45 (-194.41, 385.3) | 0.934 | 0.181 |
|  |  |  |  | 1-year visit – baseline | 785.29 (124.34, 1446.25) | 0.034 | -220 (-529.55, 89.55) | 0.42 | 565.29 (-81.19, 1211.78) | 0.14 | 0.016 |
| **MG182** |  |  |  |  |  |  |  |  |  |  |  |
| Baseline | 523.29 (136.92) | 842.65 (386.83) | 681.29 (301.13) |  |  |  |  |  |  |  |  |
| 6-month visit | 558.26 (142.98) | 632.18 (280.13) | 729.35 (249.26) | 6-month visit – baseline | -245.44 (-441.43, -49.45) | 0.001 | 258.53 (-35.12, 552.18) | 0.301 | 13.09 (-127.61, 153.79) | 0.001 | 0.001 |
| 1-year visit | 533.77 (190.72) | 740.88 (506.37) | 554.06 (238.67) | 1-year visit – 6-month visit | 133.19 (-92.45, 358.83) | 0.019 | -284 (-603.61, 35.61) | 0.612 | -150.81 (-324.11, 22.49) | 0.138 | 0.096 |
|  |  |  |  | 1-year visit – baseline | -112.25 (-385.82, 161.32) | 0.065 | -25.47 (-408.96, 358.01) | 0.068 | -137.72 (-296.17, 20.73) | 0.003 | 0.114 |
| **D-Maltose** |  |  |  |  |  |  |  |  |  |  |  |
| Baseline | 283.13 (233.18) | 187.94 (62.33) | 183.35 (42.8) |  |  |  |  |  |  |  |  |
| 6-month visit | 198.06 (104.85) | 165.53 (68.08) | 192.47 (56.64) | 6-month visit – baseline | 62.65 (-58.78, 184.09) | 0.093 | 31.53 (-26.45, 89.51) | 0.261 | 94.18 (-27.03, 215.4) | 0.031 | 0.038 |
| 1-year visit | 184.32 (82.99) | 230.29 (186.61) | 172.94 (59.11) | 1-year visit – 6-month visit | 78.51 (-16.35, 173.36) | 0.63 | -84.29 (-182.11, 13.52) | 0.628 | -5.79 (-84.18, 72.61) | 0.95 | 0.603 |
|  |  |  |  | 1-year visit – baseline | 141.16 (-0.11, 282.43) | 0.085 | -52.76 (-149.75, 44.22) | 0.301 | 88.39 (-38.87, 215.66) | 0.376 | 0.063 |
| **Tetracosanoic acid** |  |  |  |  |  |  |  |  |  |  |  |
| Baseline | 321 (121.42) | 320.41 (82.34) | 473.94 (410.37) |  |  |  |  |  |  |  |  |
| 6-month visit | 322.13 (104.82) | 329 (64.49) | 342.82 (57.71) | 6-month visit – baseline | 7.46 (-76.12, 91.04) | 0.023 | -139.71 (-342.87, 63.45) | 0.18 | -132.25 (-295.84, 31.35) | 0.63 | 0.016 |
| 1-year visit | 395.42 (325.58) | 316.06 (81.47) | 356.41 (120.42) | 1-year visit – 6-month visit | -86.23 (-255.1, 82.64) | 0.927 | 26.53 (-51.02, 104.08) | 0.233 | -59.7 (-231.31, 111.91) | 0.962 | 0.991 |
|  |  |  |  | 1-year visit – baseline | -78.77 (-265.4, 107.86) | 0.186 | -113.18 (-326.6, 100.25) | 0.135 | -191.95 (-425.72, 41.82) | 0.985 | 0.174 |
| **Alpha-Tocopherol** |  |  |  |  |  |  |  |  |  |  |  |
| Baseline | 1506.42 (749.93) | 1496.76 (705.87) | 1257.12 (493.39) |  |  |  |  |  |  |  |  |
| 6-month visit | 1806.68 (639.8) | 1279.65 (675.03) | 1852.53 (749.59) | 6-month visit – baseline | -517.38 (-1015.51, -19.25) | 0.679 | 812.53 (232.06, 1393) | 0.4 | 295.15 (-205.67, 795.97) | 0.164 | 0.506 |
| 1-year visit | 1887.9 (1065.63) | 1792.47 (567.7) | 1737.65 (811.84) | 1-year visit – 6-month visit | 431.6 (-256.09, 1119.28) | 0.887 | -627.71 (-1402.1, 146.69) | 0.151 | -196.11 (-907.19, 514.97) | 0.068 | 0.645 |
|  |  |  |  | 1-year visit – baseline | -85.78 (-755.27, 583.71) | 0.399 | 184.82 (-393.49, 763.14) | 0.515 | 99.05 (-571.66, 769.75) | 0.985 | 0.335 |

**Table S6 Multivariate analysis of metabolomics and rating scale**

|  | Compounds | t-value | p-value |
| --- | --- | --- | --- |
| BBS | Alpha-Hydroxyisobutyric acid | 1.026 | 0.307 |
|  | Hydroxyurea | 1.35 | 0.18 |
|  | Caprylic acid | -0.291 | 0.772 |
|  | L-Homoserine | 0.19 | 0.849 |
|  | Mandelic acid | 0.099 | 0.921 |
|  | D-Threitol | -1.165 | 0.247 |
|  | trans Cinnamic acid | -1.019 | 0.311 |
|  | Oxoglutaric acid | 0.837 | 0.404 |
|  | 4 Hydroxy 3 methoxybenzenemethanol | 0.181 | 0.857 |
|  | D-Xylose | 1.031 | 0.305 |
|  | Homocysteine | 1.491 | 0.139 |
|  | D-Ribose | 1.029 | 0.306 |
|  | Ribitol | 0.215 | 0.83 |
|  | L-Fucose | -0.23 | 0.818 |
|  | cis Aconitic acid | 1.329 | 0.187 |
|  | O-Phosphoethanolamine | -1.042 | 0.3 |
|  | 3-Phosphoglyceric acid | -0.518 | 0.605 |
|  | Hippuric acid | 0.055 | 0.956 |
|  | 1-Methylhistidine | -1.707 | 0.091 |
|  | Homogentisic acid | -0.738 | 0.462 |
|  | Myristic acid | 0.017 | 0.986 |
|  | Methionine sulfoxide | -1.609 | 0.111 |
|  | Indolelactic acid | 1.334 | 0.185 |
|  | Nonadecanoic acid | 0.002 | 0.998 |
|  | Docosahexaenoic acid | 0.404 | 0.687 |
|  | MG182 | -1.816 | 0.072 |
|  | Alpha-Tocopherol | 0.239 | 0.811 |
| UPDRS | Alpha-ketoisovaleric acid | 0.624 | 0.534 |
|  | Malonic acid | -1.403 | 0.164 |
|  | Hydroxyurea | -1.704 | 0.092 |
|  | Caprylic acid | 0.814 | 0.418 |
|  | Ethanolamine | -0.317 | 0.752 |
|  | L-Alloisoleucine | -0.005 | 0.996 |
|  | Fumaric acid | -0.402 | 0.689 |
|  | Pyrrole-2-carboxylic acid | -0.077 | 0.939 |
|  | Glutaric acid | -0.258 | 0.797 |
|  | Beta-Alanine | 2.164 | 0.033 |
|  | L-Homoserine | 1.036 | 0.303 |
|  | Capric acid | -0.617 | 0.539 |
|  | Mandelic acid | 0.534 | 0.594 |
|  | L-Malic acid | 2.106 | 0.038 |
|  | D-Threitol | -0.424 | 0.672 |
|  | trans Cinnamic acid | 1.907 | 0.06 |
|  | Oxoglutaric acid | -1.642 | 0.104 |
|  | 4 Hydroxy 3 methoxybenzenemethanol | -1.532 | 0.129 |
|  | D-Xylose | -0.865 | 0.389 |
|  | L-Arabinose | 2.212 | 0.03 |
|  | Homocysteine | 0.687 | 0.494 |
|  | Taurine | -0.479 | 0.633 |
|  | D-Ribose | 1.142 | 0.257 |
|  | Ribitol | -1.693 | 0.094 |
|  | Rhamnose | 1.688 | 0.095 |
|  | L-Fucose | -1.57 | 0.12 |
|  | cis Aconitic acid | -1.637 | 0.105 |
|  | O-Phosphoethanolamine | -1.205 | 0.232 |
|  | 3-Phosphoglyceric acid | 2.052 | 0.043 |
|  | Hippuric acid | 2.271 | 0.026 |
|  | 1-Methylhistidine | -0.587 | 0.559 |
|  | Myristic acid | 0.255 | 0.8 |
|  | Indolelactic acid | 0.988 | 0.326 |
|  | 5-Hydroxydopamine | 0.966 | 0.337 |
|  | L-Kynurenine | -2.08 | 0.041 |
|  | Oleic acid | -1.499 | 0.138 |
|  | Nonadecanoic acid | 0.467 | 0.642 |
|  | Arachidonic acid | 1.606 | 0.112 |
|  | Docosahexaenoic acid | -1.222 | 0.225 |
|  | Adenosine | -3.228 | 0.002 |
|  | Alpha-Lactose | 2.305 | 0.024 |
|  | Cellobiose | -0.39 | 0.698 |
|  | MG182 | -0.593 | 0.555 |
|  | D-Maltose | 1.203 | 0.232 |
|  | Tetracosanoic acid | -1.384 | 0.17 |
| UPDRS Part III | Alpha-ketoisovaleric acid | 0.833 | 0.407 |
|  | Malonic acid | -1.195 | 0.235 |
|  | Caprylic acid | -0.378 | 0.707 |
|  | Ethanolamine | -0.238 | 0.813 |
|  | L-Alloisoleucine | -0.588 | 0.558 |
|  | Pyrrole-2-carboxylic acid | -1.277 | 0.205 |
|  | Pipecolic acid | 2.11 | 0.038 |
|  | Glutaric acid | 0.617 | 0.539 |
|  | Beta-Alanine | 2.291 | 0.024 |
|  | L-Homoserine | 0.92 | 0.36 |
|  | Capric acid | 0.538 | 0.592 |
|  | Mandelic acid | 0.265 | 0.792 |
|  | L-Malic acid | 2.939 | 0.004 |
|  | D-Threitol | -1.248 | 0.216 |
|  | trans Cinnamic acid | 2.174 | 0.032 |
|  | Oxoglutaric acid | -1.738 | 0.086 |
|  | 4 Hydroxy 3 methoxybenzenemethanol | -1.042 | 0.3 |
|  | D-Xylose | -2.041 | 0.044 |
|  | L-Arabinose | 2.744 | 0.007 |
|  | Homocysteine | -0.573 | 0.568 |
|  | Taurine | 0.103 | 0.918 |
|  | D-Ribose | 1.506 | 0.136 |
|  | Ribitol | -1.564 | 0.122 |
|  | Rhamnose | 1.941 | 0.056 |
|  | L-Fucose | -2.071 | 0.041 |
|  | cis Aconitic acid | -3.099 | 0.003 |
|  | O-Phosphoethanolamine | -2.361 | 0.021 |
|  | 3-Phosphoglyceric acid | 1.878 | 0.064 |
|  | Hippuric acid | 2.683 | 0.009 |
|  | 1-Methylhistidine | -1.262 | 0.21 |
|  | Homogentisic acid | 1.178 | 0.242 |
|  | Myristic acid | 0.217 | 0.829 |
|  | Indolelactic acid | 0.919 | 0.361 |
|  | 5-Hydroxydopamine | 1.709 | 0.091 |
|  | L-Kynurenine | -2.899 | 0.005 |
|  | Oleic acid | -1.577 | 0.119 |
|  | Nonadecanoic acid | -0.002 | 0.999 |
|  | Arachidonic acid | 1.956 | 0.054 |
|  | Sucrose | 1.297 | 0.198 |
|  | Adenosine | -4.175 | <0.001 |
|  | Alpha-Lactose | 2.669 | 0.009 |
|  | Cellobiose | -0.099 | 0.922 |
|  | MG182 | -1.535 | 0.128 |
|  | D-Maltose | 0.918 | 0.361 |
|  | Tetracosanoic acid | -2.09 | 0.04 |
| TUG | Hydroxyurea | -0.376 | 0.708 |
|  | L-Homoserine | -0.371 | 0.711 |
|  | Mandelic acid | -1.091 | 0.278 |
|  | D-Threitol | 1.874 | 0.064 |
|  | trans Cinnamic acid | 0.718 | 0.474 |
|  | 4 Hydroxy 3 methoxybenzenemethanol | -0.013 | 0.989 |
|  | Homocysteine | -0.47 | 0.639 |
|  | D-Ribose | -0.477 | 0.634 |
|  | Ribitol | 0.347 | 0.729 |
|  | L-Fucose | -0.774 | 0.44 |
|  | cis Aconitic acid | -1.015 | 0.313 |
|  | 3-Phosphoglyceric acid | 0.217 | 0.829 |
|  | Hippuric acid | -0.671 | 0.503 |
|  | 1-Methylhistidine | 0.607 | 0.545 |
|  | L-Kynurenine | 1.209 | 0.229 |
|  | Nonadecanoic acid | -0.393 | 0.695 |
|  | Docosahexaenoic acid | 0.218 | 0.828 |
|  | MG182 | 0.233 | 0.816 |
|  | Alpha-Tocopherol | -0.594 | 0.554 |

**Table S7 Pathway analysis of metabolites**

| Comparisons | | Pathway | Total | Expected | Hits | Raw p | #NAME? | Holm adjust | FDR | Impact |
| --- | --- | --- | --- | --- | --- | --- | --- | --- | --- | --- |
| BBS | | Citrate cycle (TCA cycle) | 20 | 0.30968 | 2 | 0.036843 | 3.3011 | 1 | 1 | 0.10859 |
|  |  | D-Glutamine and D-glutamate metabolism | 6 | 0.092903 | 1 | 0.089519 | 2.4133 | 1 | 1 | 0 |
|  |  | Ubiquinone and other terpenoid-quinone biosynthesis | 9 | 0.13935 | 1 | 0.13135 | 2.0299 | 1 | 1 | 0 |
|  |  | Phenylalanine metabolism | 10 | 0.15484 | 1 | 0.14488 | 1.9319 | 1 | 1 | 0 |
|  |  | Fatty acid biosynthesis | 47 | 0.72774 | 2 | 0.16313 | 1.8132 | 1 | 1 | 0 |
|  |  | Arginine biosynthesis | 14 | 0.21677 | 1 | 0.197 | 1.6246 | 1 | 1 | 0 |
|  |  | Butanoate metabolism | 15 | 0.23226 | 1 | 0.20955 | 1.5628 | 1 | 1 | 0 |
|  |  | Histidine metabolism | 16 | 0.24774 | 1 | 0.2219 | 1.5055 | 1 | 1 | 0 |
|  |  | Pentose and glucuronate interconversions | 18 | 0.27871 | 1 | 0.24607 | 1.4021 | 1 | 1 | 0.07812 |
|  |  | Sphingolipid metabolism | 21 | 0.32516 | 1 | 0.28097 | 1.2695 | 1 | 1 | 0.0142 |
|  |  | Pentose phosphate pathway | 22 | 0.34065 | 1 | 0.29226 | 1.2301 | 1 | 1 | 0 |
|  |  | Alanine, aspartate and glutamate metabolism | 28 | 0.43355 | 1 | 0.35649 | 1.0314 | 1 | 1 | 0.04808 |
|  |  | Glyoxylate and dicarboxylate metabolism | 32 | 0.49548 | 1 | 0.39617 | 0.92591 | 1 | 1 | 0.02381 |
|  |  | Biosynthesis of unsaturated fatty acids | 36 | 0.55742 | 1 | 0.4335 | 0.83587 | 1 | 1 | 0 |
|  |  | Glycerophospholipid metabolism | 36 | 0.55742 | 1 | 0.4335 | 0.83587 | 1 | 1 | 0.02423 |
|  |  | Tyrosine metabolism | 42 | 0.65032 | 1 | 0.48537 | 0.72284 | 1 | 1 | 0.06467 |
| UPDRS | | Citrate cycle (TCA cycle) | 20 | 0.52903 | 4 | 0.0015042 | 6.4995 | 0.12636 | 0.12636 | 0.18252 |
|  |  | Arginine biosynthesis | 14 | 0.37032 | 2 | 0.050822 | 2.9794 | 1 | 1 | 0 |
|  |  | Biosynthesis of unsaturated fatty acids | 36 | 0.95226 | 3 | 0.067064 | 2.7021 | 1 | 1 | 0 |
|  |  | Pentose and glucuronate interconversions | 18 | 0.47613 | 2 | 0.079978 | 2.526 | 1 | 1 | 0.07812 |
|  |  | Pantothenate and CoA biosynthesis | 19 | 0.50258 | 2 | 0.087928 | 2.4312 | 1 | 1 | 0.02143 |
|  |  | Pyruvate metabolism | 22 | 0.58194 | 2 | 0.11308 | 2.1797 | 1 | 1 | 0.0311 |
|  |  | Fatty acid biosynthesis | 47 | 1.2432 | 3 | 0.125 | 2.0794 | 1 | 1 | 0 |
|  |  | D-Glutamine and D-glutamate metabolism | 6 | 0.15871 | 1 | 0.1488 | 1.9051 | 1 | 1 | 0 |
|  |  | Alanine, aspartate and glutamate metabolism | 28 | 0.74065 | 2 | 0.16784 | 1.7847 | 1 | 1 | 0.05048 |
|  |  | Valine, leucine and isoleucine biosynthesis | 8 | 0.21161 | 1 | 0.19342 | 1.6429 | 1 | 1 | 0 |
|  |  | Taurine and hypotaurine metabolism | 8 | 0.21161 | 1 | 0.19342 | 1.6429 | 1 | 1 | 0.42857 |
|  |  | Glyoxylate and dicarboxylate metabolism | 32 | 0.84645 | 2 | 0.20651 | 1.5774 | 1 | 1 | 0.02381 |
|  |  | Phenylalanine metabolism | 10 | 0.26452 | 1 | 0.23576 | 1.445 | 1 | 1 | 0 |
|  |  | Glycerophospholipid metabolism | 36 | 0.95226 | 2 | 0.24608 | 1.4021 | 1 | 1 | 0.03747 |
|  |  | Butanoate metabolism | 15 | 0.39677 | 1 | 0.33233 | 1.1016 | 1 | 1 | 0 |
|  |  | Histidine metabolism | 16 | 0.42323 | 1 | 0.35017 | 1.0493 | 1 | 1 | 0 |
|  |  | Starch and sucrose metabolism | 18 | 0.47613 | 1 | 0.38445 | 0.95594 | 1 | 1 | 0.07306 |
|  |  | Sphingolipid metabolism | 21 | 0.55548 | 1 | 0.43259 | 0.83796 | 1 | 1 | 0.0142 |
|  |  | beta-Alanine metabolism | 21 | 0.55548 | 1 | 0.43259 | 0.83796 | 1 | 1 | 0.39925 |
|  |  | Pentose phosphate pathway | 22 | 0.58194 | 1 | 0.44781 | 0.80339 | 1 | 1 | 0 |
|  |  | Propanoate metabolism | 23 | 0.60839 | 1 | 0.46262 | 0.77084 | 1 | 1 | 0 |
|  |  | Galactose metabolism | 27 | 0.71419 | 1 | 0.51811 | 0.65757 | 1 | 1 | 0.11032 |
|  |  | Arachidonic acid metabolism | 36 | 0.95226 | 1 | 0.6233 | 0.47273 | 1 | 1 | 0.3135 |
|  |  | Pyrimidine metabolism | 39 | 1.0316 | 1 | 0.6531 | 0.42603 | 1 | 1 | 0 |
|  |  | Valine, leucine and isoleucine degradation | 40 | 1.0581 | 1 | 0.66251 | 0.41172 | 1 | 1 | 0.01084 |
|  |  | Tryptophan metabolism | 41 | 1.0845 | 1 | 0.67167 | 0.39798 | 1 | 1 | 0.09417 |
|  |  | Tyrosine metabolism | 42 | 1.111 | 1 | 0.6806 | 0.38479 | 1 | 1 | 0.02463 |
|  |  | Primary bile acid biosynthesis | 46 | 1.2168 | 1 | 0.71397 | 0.33691 | 1 | 1 | 0.00758 |
|  |  | Purine metabolism | 65 | 1.7194 | 1 | 0.83137 | 0.18469 | 1 | 1 | 0.00124 |
| UPDRS Part III | | Citrate cycle (TCA cycle) | 20 | 0.52903 | 3 | 0.01433 | 4.2454 | 1 | 1 | 0.15271 |
|  |  | Pentose and glucuronate interconversions | 18 | 0.47613 | 2 | 0.079978 | 2.526 | 1 | 1 | 0.07812 |
|  |  | Starch and sucrose metabolism | 18 | 0.47613 | 2 | 0.079978 | 2.526 | 1 | 1 | 0.12329 |
|  |  | Pantothenate and CoA biosynthesis | 19 | 0.50258 | 2 | 0.087928 | 2.4312 | 1 | 1 | 0.02143 |
|  |  | Fatty acid biosynthesis | 47 | 1.2432 | 3 | 0.125 | 2.0794 | 1 | 1 | 0 |
|  |  | D-Glutamine and D-glutamate metabolism | 6 | 0.15871 | 1 | 0.1488 | 1.9051 | 1 | 1 | 0 |
|  |  | Galactose metabolism | 27 | 0.71419 | 2 | 0.15839 | 1.8427 | 1 | 1 | 0.1492 |
|  |  | Valine, leucine and isoleucine biosynthesis | 8 | 0.21161 | 1 | 0.19342 | 1.6429 | 1 | 1 | 0 |
|  |  | Taurine and hypotaurine metabolism | 8 | 0.21161 | 1 | 0.19342 | 1.6429 | 1 | 1 | 0.42857 |
|  |  | Glyoxylate and dicarboxylate metabolism | 32 | 0.84645 | 2 | 0.20651 | 1.5774 | 1 | 1 | 0.02381 |
|  |  | Ubiquinone and other terpenoid-quinone biosynthesis | 9 | 0.23806 | 1 | 0.21487 | 1.5377 | 1 | 1 | 0 |
|  |  | Phenylalanine metabolism | 10 | 0.26452 | 1 | 0.23576 | 1.445 | 1 | 1 | 0 |
|  |  | Biosynthesis of unsaturated fatty acids | 36 | 0.95226 | 2 | 0.24608 | 1.4021 | 1 | 1 | 0 |
|  |  | Glycerophospholipid metabolism | 36 | 0.95226 | 2 | 0.24608 | 1.4021 | 1 | 1 | 0.03747 |
|  |  | Arginine biosynthesis | 14 | 0.37032 | 1 | 0.31402 | 1.1583 | 1 | 1 | 0 |
|  |  | Butanoate metabolism | 15 | 0.39677 | 1 | 0.33233 | 1.1016 | 1 | 1 | 0 |
|  |  | Histidine metabolism | 16 | 0.42323 | 1 | 0.35017 | 1.0493 | 1 | 1 | 0 |
|  |  | Sphingolipid metabolism | 21 | 0.55548 | 1 | 0.43259 | 0.83796 | 1 | 1 | 0.0142 |
|  |  | beta-Alanine metabolism | 21 | 0.55548 | 1 | 0.43259 | 0.83796 | 1 | 1 | 0.39925 |
|  |  | Pentose phosphate pathway | 22 | 0.58194 | 1 | 0.44781 | 0.80339 | 1 | 1 | 0 |
|  |  | Pyruvate metabolism | 22 | 0.58194 | 1 | 0.44781 | 0.80339 | 1 | 1 | 0.0311 |
|  |  | Propanoate metabolism | 23 | 0.60839 | 1 | 0.46262 | 0.77084 | 1 | 1 | 0 |
|  |  | Lysine degradation | 25 | 0.66129 | 1 | 0.4911 | 0.7111 | 1 | 1 | 0 |
|  |  | Alanine, aspartate and glutamate metabolism | 28 | 0.74065 | 1 | 0.53108 | 0.63284 | 1 | 1 | 0.04808 |
|  |  | Arachidonic acid metabolism | 36 | 0.95226 | 1 | 0.6233 | 0.47273 | 1 | 1 | 0.3135 |
|  |  | Pyrimidine metabolism | 39 | 1.0316 | 1 | 0.6531 | 0.42603 | 1 | 1 | 0 |
|  |  | Valine, leucine and isoleucine degradation | 40 | 1.0581 | 1 | 0.66251 | 0.41172 | 1 | 1 | 0.01084 |
|  |  | Tryptophan metabolism | 41 | 1.0845 | 1 | 0.67167 | 0.39798 | 1 | 1 | 0.09417 |
|  |  | Tyrosine metabolism | 42 | 1.111 | 1 | 0.6806 | 0.38479 | 1 | 1 | 0.06467 |
|  |  | Primary bile acid biosynthesis | 46 | 1.2168 | 1 | 0.71397 | 0.33691 | 1 | 1 | 0.00758 |
|  |  | Purine metabolism | 65 | 1.7194 | 1 | 0.83137 | 0.18469 | 1 | 1 | 0.00124 |
| TUG | | Phenylalanine metabolism | 10 | 0.11613 | 1 | 0.11055 | 2.2023 | 1 | 1 | 0 |
|  |  | Histidine metabolism | 16 | 0.18581 | 1 | 0.17123 | 1.7648 | 1 | 1 | 0 |
|  |  | Citrate cycle (TCA cycle) | 20 | 0.23226 | 1 | 0.20948 | 1.5631 | 1 | 1 | 0.05003 |
|  |  | Pentose phosphate pathway | 22 | 0.25548 | 1 | 0.22798 | 1.4785 | 1 | 1 | 0 |
|  |  | Glyoxylate and dicarboxylate metabolism | 32 | 0.37161 | 1 | 0.31449 | 1.1568 | 1 | 1 | 0.02381 |
|  |  | Biosynthesis of unsaturated fatty acids | 36 | 0.41806 | 1 | 0.34647 | 1.06 | 1 | 1 | 0 |
|  |  | Tryptophan metabolism | 41 | 0.47613 | 1 | 0.38445 | 0.95594 | 1 | 1 | 0.09417 |
| Tai Chi v.s. Control | Baseline v.s. 6-month visit | Cysteine and methionine metabolism | 33 | 0.31935 | 3 | 0.0033631 | 5.6949 | 0.2825 | 0.2825 | 0.10446 |
|  |  | Arginine biosynthesis | 14 | 0.13548 | 2 | 0.0074414 | 4.9007 | 0.61764 | 0.31254 | 0 |
|  |  | Pantothenate and CoA biosynthesis | 19 | 0.18387 | 2 | 0.013597 | 4.2979 | 1 | 0.38073 | 0 |
|  |  | Alanine, aspartate and glutamate metabolism | 28 | 0.27097 | 2 | 0.028584 | 3.5549 | 1 | 0.60026 | 0.22356 |
|  |  | Glycine, serine and threonine metabolism | 33 | 0.31935 | 2 | 0.038829 | 3.2486 | 1 | 0.65233 | 0.0455 |
|  |  | Valine, leucine and isoleucine biosynthesis | 8 | 0.077419 | 1 | 0.075011 | 2.5901 | 1 | 0.91579 | 0 |
|  |  | Aminoacyl-tRNA biosynthesis | 48 | 0.46452 | 2 | 0.076316 | 2.5729 | 1 | 0.91579 | 0 |
|  |  | alpha-Linolenic acid metabolism | 13 | 0.12581 | 1 | 0.11919 | 2.127 | 1 | 1 | 0.33333 |
|  |  | Nicotinate and nicotinamide metabolism | 15 | 0.14516 | 1 | 0.1363 | 1.9929 | 1 | 1 | 0 |
|  |  | Histidine metabolism | 16 | 0.15484 | 1 | 0.14474 | 1.9328 | 1 | 1 | 0 |
|  |  | Glycerolipid metabolism | 16 | 0.15484 | 1 | 0.14474 | 1.9328 | 1 | 1 | 0.04361 |
|  |  | Citrate cycle (TCA cycle) | 20 | 0.19355 | 1 | 0.17774 | 1.7274 | 1 | 1 | 0.04634 |
|  |  | beta-Alanine metabolism | 21 | 0.20323 | 1 | 0.1858 | 1.6831 | 1 | 1 | 0 |
|  |  | Pyruvate metabolism | 22 | 0.2129 | 1 | 0.19379 | 1.641 | 1 | 1 | 0.20684 |
|  |  | Glycolysis / Gluconeogenesis | 26 | 0.25161 | 1 | 0.22501 | 1.4916 | 1 | 1 | 0.10044 |
|  |  | Glyoxylate and dicarboxylate metabolism | 32 | 0.30968 | 1 | 0.26974 | 1.3103 | 1 | 1 | 0 |
|  |  | Biosynthesis of unsaturated fatty acids | 36 | 0.34839 | 1 | 0.29821 | 1.21 | 1 | 1 | 0 |
|  |  | Glycerophospholipid metabolism | 36 | 0.34839 | 1 | 0.29821 | 1.21 | 1 | 1 | 0.08093 |
|  |  | Amino sugar and nucleotide sugar metabolism | 37 | 0.35806 | 1 | 0.30516 | 1.1869 | 1 | 1 | 0 |
|  |  | Arginine and proline metabolism | 38 | 0.36774 | 1 | 0.31205 | 1.1646 | 1 | 1 | 0 |
|  |  | Valine, leucine and isoleucine degradation | 40 | 0.3871 | 1 | 0.32564 | 1.122 | 1 | 1 | 0.01084 |
|  |  | Tyrosine metabolism | 42 | 0.40645 | 1 | 0.33897 | 1.0818 | 1 | 1 | 0 |
|  |  | Fatty acid biosynthesis | 47 | 0.45484 | 1 | 0.37124 | 0.9909 | 1 | 1 | 0 |
|  |  | Purine metabolism | 65 | 0.62903 | 1 | 0.47565 | 0.74308 | 1 | 1 | 0 |
| Tai Chi v.s. Control | 6-month visit v.s. 12-month visit | Arginine biosynthesis | 14 | 0.16258 | 3 | 0.0004425 | 7.7231 | 0.03717 | 0.03717 | 0 |
|  |  | Citrate cycle (TCA cycle) | 20 | 0.23226 | 3 | 0.0013265 | 6.6252 | 0.1101 | 0.055712 | 0.1211 |
|  |  | Alanine, aspartate and glutamate metabolism | 28 | 0.32516 | 3 | 0.0035956 | 5.628 | 0.29484 | 0.10068 | 0.05048 |
|  |  | Purine metabolism | 65 | 0.75484 | 4 | 0.0055337 | 5.1969 | 0.44823 | 0.11621 | 0.01775 |
|  |  | Butanoate metabolism | 15 | 0.17419 | 2 | 0.012234 | 4.4035 | 0.97875 | 0.20554 | 0 |
|  |  | D-Glutamine and D-glutamate metabolism | 6 | 0.069677 | 1 | 0.067792 | 2.6913 | 1 | 0.94909 | 0 |
|  |  | Tyrosine metabolism | 42 | 0.48774 | 2 | 0.083408 | 2.484 | 1 | 1 | 0.0893 |
|  |  | Ubiquinone and other terpenoid-quinone biosynthesis | 9 | 0.10452 | 1 | 0.10004 | 2.3022 | 1 | 1 | 0 |
|  |  | Histidine metabolism | 16 | 0.18581 | 1 | 0.17123 | 1.7648 | 1 | 1 | 0 |
|  |  | Glycerolipid metabolism | 16 | 0.18581 | 1 | 0.17123 | 1.7648 | 1 | 1 | 0.04361 |
|  |  | Fructose and mannose metabolism | 20 | 0.23226 | 1 | 0.20948 | 1.5631 | 1 | 1 | 0 |
|  |  | Pyruvate metabolism | 22 | 0.25548 | 1 | 0.22798 | 1.4785 | 1 | 1 | 0 |
|  |  | Propanoate metabolism | 23 | 0.2671 | 1 | 0.23708 | 1.4394 | 1 | 1 | 0 |
|  |  | Galactose metabolism | 27 | 0.31355 | 1 | 0.27245 | 1.3003 | 1 | 1 | 0 |
|  |  | Cysteine and methionine metabolism | 33 | 0.38323 | 1 | 0.32262 | 1.1313 | 1 | 1 | 0 |
|  |  | Glycerophospholipid metabolism | 36 | 0.41806 | 1 | 0.34647 | 1.06 | 1 | 1 | 0.08093 |
|  |  | Amino sugar and nucleotide sugar metabolism | 37 | 0.42968 | 1 | 0.35424 | 1.0378 | 1 | 1 | 0 |
| Tai Chi v.s. Control | Baseline v.s. 12-month visit | Arginine biosynthesis | 14 | 0.14452 | 2 | 0.0084606 | 4.7723 | 0.71069 | 0.71069 | 0 |
|  |  | Purine metabolism | 65 | 0.67097 | 3 | 0.02666 | 3.6246 | 1 | 1 | 0.01775 |
|  |  | Cysteine and methionine metabolism | 33 | 0.34065 | 2 | 0.043793 | 3.1283 | 1 | 1 | 0.10446 |
|  |  | Aminoacyl-tRNA biosynthesis | 48 | 0.49548 | 2 | 0.085538 | 2.4588 | 1 | 1 | 0 |
|  |  | Histidine metabolism | 16 | 0.16516 | 1 | 0.15366 | 1.873 | 1 | 1 | 0.22131 |
|  |  | Citrate cycle (TCA cycle) | 20 | 0.20645 | 1 | 0.18845 | 1.6689 | 1 | 1 | 0.02981 |
|  |  | beta-Alanine metabolism | 21 | 0.21677 | 1 | 0.19694 | 1.6249 | 1 | 1 | 0 |
|  |  | Pyruvate metabolism | 22 | 0.2271 | 1 | 0.20534 | 1.5831 | 1 | 1 | 0 |
|  |  | Lysine degradation | 25 | 0.25806 | 1 | 0.23006 | 1.4694 | 1 | 1 | 0 |
|  |  | Alanine, aspartate and glutamate metabolism | 28 | 0.28903 | 1 | 0.25406 | 1.3702 | 1 | 1 | 0.0024 |
|  |  | Glycine, serine and threonine metabolism | 33 | 0.34065 | 1 | 0.2925 | 1.2293 | 1 | 1 | 0.0455 |
|  |  | Amino sugar and nucleotide sugar metabolism | 37 | 0.38194 | 1 | 0.32191 | 1.1335 | 1 | 1 | 0 |
|  |  | Tyrosine metabolism | 42 | 0.43355 | 1 | 0.35706 | 1.0299 | 1 | 1 | 0.02463 |
|  |  | Fatty acid biosynthesis | 47 | 0.48516 | 1 | 0.39049 | 0.94034 | 1 | 1 | 0 |
| Brisk walking v.s. control | Baseline v.s. 6-month visit | Arginine biosynthesis | 14 | 0.099355 | 3 | 9.29E-05 | 9.284 | 0.0078032 | 0.007803 | 0.11675 |
|  |  | Alanine, aspartate and glutamate metabolism | 28 | 0.19871 | 3 | 0.0007916 | 7.1415 | 0.065703 | 0.033247 | 0.42068 |
|  |  | Aminoacyl-tRNA biosynthesis | 48 | 0.34065 | 3 | 0.0038642 | 5.556 | 0.31686 | 0.1082 | 0 |
|  |  | Histidine metabolism | 16 | 0.11355 | 2 | 0.0052069 | 5.2578 | 0.42176 | 0.10935 | 0 |
|  |  | Pantothenate and CoA biosynthesis | 19 | 0.13484 | 2 | 0.0073339 | 4.9152 | 0.58671 | 0.12321 | 0 |
|  |  | Glyoxylate and dicarboxylate metabolism | 32 | 0.2271 | 2 | 0.020226 | 3.9008 | 1 | 0.25737 | 0 |
|  |  | Cysteine and methionine metabolism | 33 | 0.23419 | 2 | 0.021447 | 3.8422 | 1 | 0.25737 | 0.10446 |
|  |  | Arginine and proline metabolism | 38 | 0.26968 | 2 | 0.028007 | 3.5753 | 1 | 0.29407 | 0.086 |
|  |  | Nitrogen metabolism | 6 | 0.042581 | 1 | 0.041899 | 3.1725 | 1 | 0.35195 | 0 |
|  |  | D-Glutamine and D-glutamate metabolism | 6 | 0.042581 | 1 | 0.041899 | 3.1725 | 1 | 0.35195 | 0.5 |
|  |  | Valine, leucine and isoleucine biosynthesis | 8 | 0.056774 | 1 | 0.055506 | 2.8913 | 1 | 0.42387 | 0 |
|  |  | Purine metabolism | 65 | 0.46129 | 2 | 0.074627 | 2.5953 | 1 | 0.52239 | 0 |
|  |  | alpha-Linolenic acid metabolism | 13 | 0.092258 | 1 | 0.08876 | 2.4218 | 1 | 0.56986 | 0.33333 |
|  |  | Butanoate metabolism | 15 | 0.10645 | 1 | 0.10176 | 2.2851 | 1 | 0.56986 | 0 |
|  |  | Nicotinate and nicotinamide metabolism | 15 | 0.10645 | 1 | 0.10176 | 2.2851 | 1 | 0.56986 | 0 |
|  |  | Pentose and glucuronate interconversions | 18 | 0.12774 | 1 | 0.12095 | 2.1124 | 1 | 0.63496 | 0.07812 |
|  |  | Citrate cycle (TCA cycle) | 20 | 0.14194 | 1 | 0.13353 | 2.0134 | 1 | 0.64524 | 0.04634 |
|  |  | beta-Alanine metabolism | 21 | 0.14903 | 1 | 0.13976 | 1.9678 | 1 | 0.64524 | 0 |
|  |  | Pyruvate metabolism | 22 | 0.15613 | 1 | 0.14595 | 1.9245 | 1 | 0.64524 | 0.20684 |
|  |  | Glycolysis / Gluconeogenesis | 26 | 0.18452 | 1 | 0.1703 | 1.7702 | 1 | 0.69581 | 0.10044 |
|  |  | Galactose metabolism | 27 | 0.19161 | 1 | 0.17629 | 1.7356 | 1 | 0.69581 | 0.11032 |
|  |  | Glutathione metabolism | 28 | 0.19871 | 1 | 0.18224 | 1.7024 | 1 | 0.69581 | 0.01966 |
|  |  | Porphyrin and chlorophyll metabolism | 30 | 0.2129 | 1 | 0.19402 | 1.6398 | 1 | 0.70859 | 0 |
|  |  | Glycine, serine and threonine metabolism | 33 | 0.23419 | 1 | 0.2114 | 1.554 | 1 | 0.73991 | 0 |
|  |  | Biosynthesis of unsaturated fatty acids | 36 | 0.25548 | 1 | 0.22844 | 1.4765 | 1 | 0.76757 | 0 |
|  |  | Valine, leucine and isoleucine degradation | 40 | 0.28387 | 1 | 0.25064 | 1.3837 | 1 | 0.80978 | 0.01084 |
|  |  | Tyrosine metabolism | 42 | 0.29806 | 1 | 0.26153 | 1.3412 | 1 | 0.81364 | 0 |
| Brisk walking v.s. control | 6-month visit v.s. 12-month visit | Alanine, aspartate and glutamate metabolism | 28 | 0.27097 | 4 | 0.00010168 | 9.1937 | 0.0085408 | 0.008541 | 0.0024 |
|  |  | Arginine biosynthesis | 14 | 0.13548 | 2 | 0.0074414 | 4.9007 | 0.61764 | 0.31254 | 0 |
|  |  | Citrate cycle (TCA cycle) | 20 | 0.19355 | 2 | 0.015024 | 4.1981 | 1 | 0.32369 | 0.07615 |
|  |  | Pyruvate metabolism | 22 | 0.2129 | 2 | 0.018063 | 4.0139 | 1 | 0.32369 | 0.20684 |
|  |  | Neomycin, kanamycin and gentamicin biosynthesis | 2 | 0.019355 | 1 | 0.019267 | 3.9493 | 1 | 0.32369 | 0 |
|  |  | Tyrosine metabolism | 42 | 0.40645 | 2 | 0.060225 | 2.8097 | 1 | 0.84315 | 0.02463 |
|  |  | Aminoacyl-tRNA biosynthesis | 48 | 0.46452 | 2 | 0.076316 | 2.5729 | 1 | 0.91579 | 0 |
|  |  | Purine metabolism | 65 | 0.62903 | 2 | 0.1281 | 2.055 | 1 | 1 | 0.01651 |
|  |  | Glycerolipid metabolism | 16 | 0.15484 | 1 | 0.14474 | 1.9328 | 1 | 1 | 0.04361 |
|  |  | Starch and sucrose metabolism | 18 | 0.17419 | 1 | 0.16139 | 1.8239 | 1 | 1 | 0.4207 |
|  |  | Selenocompound metabolism | 20 | 0.19355 | 1 | 0.17774 | 1.7274 | 1 | 1 | 0 |
|  |  | Pentose phosphate pathway | 22 | 0.2129 | 1 | 0.19379 | 1.641 | 1 | 1 | 0 |
|  |  | Glycolysis / Gluconeogenesis | 26 | 0.25161 | 1 | 0.22501 | 1.4916 | 1 | 1 | 0.10044 |
|  |  | Galactose metabolism | 27 | 0.26129 | 1 | 0.23264 | 1.4583 | 1 | 1 | 0.03499 |
|  |  | Glyoxylate and dicarboxylate metabolism | 32 | 0.30968 | 1 | 0.26974 | 1.3103 | 1 | 1 | 0 |
|  |  | Glycine, serine and threonine metabolism | 33 | 0.31935 | 1 | 0.27696 | 1.2839 | 1 | 1 | 0 |
|  |  | Cysteine and methionine metabolism | 33 | 0.31935 | 1 | 0.27696 | 1.2839 | 1 | 1 | 0 |
|  |  | Glycerophospholipid metabolism | 36 | 0.34839 | 1 | 0.29821 | 1.21 | 1 | 1 | 0.08093 |
|  |  | Arginine and proline metabolism | 38 | 0.36774 | 1 | 0.31205 | 1.1646 | 1 | 1 | 0 |
| Brisk walking v.s. control | Baseline v.s. 12-month visit | Cysteine and methionine metabolism | 33 | 0.25548 | 4 | 7.49E-05 | 9.4991 | 0.0062932 | 0.006293 | 0.20038 |
|  |  | Taurine and hypotaurine metabolism | 8 | 0.061935 | 2 | 0.0015 | 6.5023 | 0.1245 | 0.063002 | 0.42857 |
|  |  | Aminoacyl-tRNA biosynthesis | 48 | 0.37161 | 3 | 0.0050408 | 5.2902 | 0.41334 | 0.14114 | 0 |
|  |  | Purine metabolism | 65 | 0.50323 | 3 | 0.011808 | 4.439 | 0.95647 | 0.24797 | 0.01775 |
|  |  | Alanine, aspartate and glutamate metabolism | 28 | 0.21677 | 2 | 0.018578 | 3.9858 | 1 | 0.30476 | 0.11378 |
|  |  | Glyoxylate and dicarboxylate metabolism | 32 | 0.24774 | 2 | 0.023961 | 3.7313 | 1 | 0.30476 | 0 |
|  |  | Glycine, serine and threonine metabolism | 33 | 0.25548 | 2 | 0.025397 | 3.6731 | 1 | 0.30476 | 0 |
|  |  | D-Glutamine and D-glutamate metabolism | 6 | 0.046452 | 1 | 0.045634 | 3.0871 | 1 | 0.42592 | 0 |
|  |  | Nitrogen metabolism | 6 | 0.046452 | 1 | 0.045634 | 3.0871 | 1 | 0.42592 | 0 |
|  |  | Thiamine metabolism | 7 | 0.054194 | 1 | 0.053051 | 2.9365 | 1 | 0.44563 | 0 |
|  |  | Arginine biosynthesis | 14 | 0.10839 | 1 | 0.10351 | 2.2681 | 1 | 0.79045 | 0 |
|  |  | Pantothenate and CoA biosynthesis | 19 | 0.1471 | 1 | 0.13803 | 1.9803 | 1 | 0.93555 | 0 |
|  |  | Citrate cycle (TCA cycle) | 20 | 0.15484 | 1 | 0.14479 | 1.9325 | 1 | 0.93555 | 0.04634 |
|  |  | Pyruvate metabolism | 22 | 0.17032 | 1 | 0.15815 | 1.8442 | 1 | 0.94893 | 0.20684 |
|  |  | Glycolysis / Gluconeogenesis | 26 | 0.20129 | 1 | 0.18432 | 1.6911 | 1 | 1 | 0.10044 |
|  |  | Glutathione metabolism | 28 | 0.21677 | 1 | 0.19711 | 1.624 | 1 | 1 | 0.00343 |
|  |  | Arginine and proline metabolism | 38 | 0.29419 | 1 | 0.25839 | 1.3533 | 1 | 1 | 0 |
|  |  | Pyrimidine metabolism | 39 | 0.30194 | 1 | 0.26428 | 1.3308 | 1 | 1 | 0 |
|  |  | Tyrosine metabolism | 42 | 0.32516 | 1 | 0.28168 | 1.267 | 1 | 1 | 0 |
|  |  | Primary bile acid biosynthesis | 46 | 0.35613 | 1 | 0.30429 | 1.1898 | 1 | 1 | 0.00758 |
|  |  | Fatty acid biosynthesis | 47 | 0.36387 | 1 | 0.30985 | 1.1717 | 1 | 1 | 0 |
| Tai Chi v.s. Brisk Walking | Baseline v.s. 6-month visit | Biosynthesis of unsaturated fatty acids | 36 | 0.30194 | 3 | 0.0028068 | 5.8757 | 0.23577 | 0.23459 | 0 |
|  |  | Arginine biosynthesis | 14 | 0.11742 | 2 | 0.0055854 | 5.1876 | 0.46359 | 0.23459 | 0 |
|  |  | Purine metabolism | 65 | 0.54516 | 3 | 0.014896 | 4.2067 | 1 | 0.41709 | 0.01651 |
|  |  | Cysteine and methionine metabolism | 33 | 0.27677 | 2 | 0.029619 | 3.5193 | 1 | 0.59229 | 0 |
|  |  | Linoleic acid metabolism | 5 | 0.041935 | 1 | 0.04129 | 3.1871 | 1 | 0.59229 | 1 |
|  |  | D-Glutamine and D-glutamate metabolism | 6 | 0.050323 | 1 | 0.049357 | 3.0087 | 1 | 0.59229 | 0 |
|  |  | Nitrogen metabolism | 6 | 0.050323 | 1 | 0.049357 | 3.0087 | 1 | 0.59229 | 0 |
|  |  | alpha-Linolenic acid metabolism | 13 | 0.10903 | 1 | 0.10409 | 2.2625 | 1 | 1 | 0.33333 |
|  |  | Pentose and glucuronate interconversions | 18 | 0.15097 | 1 | 0.14139 | 1.9562 | 1 | 1 | 0.07812 |
|  |  | Alanine, aspartate and glutamate metabolism | 28 | 0.23484 | 1 | 0.21173 | 1.5524 | 1 | 1 | 0.11378 |
|  |  | Glyoxylate and dicarboxylate metabolism | 32 | 0.26839 | 1 | 0.23835 | 1.434 | 1 | 1 | 0 |
|  |  | Glycine, serine and threonine metabolism | 33 | 0.27677 | 1 | 0.24487 | 1.407 | 1 | 1 | 0.0455 |
|  |  | Fatty acid elongation | 39 | 0.3271 | 1 | 0.28293 | 1.2625 | 1 | 1 | 0 |
|  |  | Fatty acid degradation | 39 | 0.3271 | 1 | 0.28293 | 1.2625 | 1 | 1 | 0 |
|  |  | Pyrimidine metabolism | 39 | 0.3271 | 1 | 0.28293 | 1.2625 | 1 | 1 | 0 |
|  |  | Fatty acid biosynthesis | 47 | 0.39419 | 1 | 0.33094 | 1.1058 | 1 | 1 | 0.01473 |
|  |  | Aminoacyl-tRNA biosynthesis | 48 | 0.40258 | 1 | 0.33672 | 1.0885 | 1 | 1 | 0 |
| Tai Chi v.s. Brisk Walking | 6-month visit v.s. 12-month visit | Glyoxylate and dicarboxylate metabolism | 32 | 0.2271 | 3 | 0.0011799 | 6.7423 | 0.099112 | 0.099112 | 0.10582 |
|  |  | Aminoacyl-tRNA biosynthesis | 48 | 0.34065 | 3 | 0.0038642 | 5.556 | 0.32073 | 0.1623 | 0 |
|  |  | Neomycin, kanamycin and gentamicin biosynthesis | 2 | 0.014194 | 1 | 0.014148 | 4.2582 | 1 | 0.30026 | 0 |
|  |  | Alanine, aspartate and glutamate metabolism | 28 | 0.19871 | 2 | 0.015655 | 4.157 | 1 | 0.30026 | 0.11378 |
|  |  | Cysteine and methionine metabolism | 33 | 0.23419 | 2 | 0.021447 | 3.8422 | 1 | 0.30026 | 0.10446 |
|  |  | Glycine, serine and threonine metabolism | 33 | 0.23419 | 2 | 0.021447 | 3.8422 | 1 | 0.30026 | 0.24577 |
|  |  | D-Glutamine and D-glutamate metabolism | 6 | 0.042581 | 1 | 0.041899 | 3.1725 | 1 | 0.43994 | 0 |
|  |  | Nitrogen metabolism | 6 | 0.042581 | 1 | 0.041899 | 3.1725 | 1 | 0.43994 | 0 |
|  |  | alpha-Linolenic acid metabolism | 13 | 0.092258 | 1 | 0.08876 | 2.4218 | 1 | 0.80036 | 0.33333 |
|  |  | Arginine biosynthesis | 14 | 0.099355 | 1 | 0.095281 | 2.3509 | 1 | 0.80036 | 0 |
|  |  | Starch and sucrose metabolism | 18 | 0.12774 | 1 | 0.12095 | 2.1124 | 1 | 0.87568 | 0.4207 |
|  |  | Citrate cycle (TCA cycle) | 20 | 0.14194 | 1 | 0.13353 | 2.0134 | 1 | 0.87568 | 0.04634 |
|  |  | Pentose phosphate pathway | 22 | 0.15613 | 1 | 0.14595 | 1.9245 | 1 | 0.87568 | 0 |
|  |  | Pyruvate metabolism | 22 | 0.15613 | 1 | 0.14595 | 1.9245 | 1 | 0.87568 | 0.20684 |
|  |  | Glycolysis / Gluconeogenesis | 26 | 0.18452 | 1 | 0.1703 | 1.7702 | 1 | 0.90046 | 0.10044 |
|  |  | Galactose metabolism | 27 | 0.19161 | 1 | 0.17629 | 1.7356 | 1 | 0.90046 | 0.03499 |
|  |  | Glutathione metabolism | 28 | 0.19871 | 1 | 0.18224 | 1.7024 | 1 | 0.90046 | 0.08873 |
|  |  | Porphyrin and chlorophyll metabolism | 30 | 0.2129 | 1 | 0.19402 | 1.6398 | 1 | 0.90542 | 0 |
|  |  | Biosynthesis of unsaturated fatty acids | 36 | 0.25548 | 1 | 0.22844 | 1.4765 | 1 | 0.93603 | 0 |
|  |  | Amino sugar and nucleotide sugar metabolism | 37 | 0.26258 | 1 | 0.23405 | 1.4522 | 1 | 0.93603 | 0 |
|  |  | Arginine and proline metabolism | 38 | 0.26968 | 1 | 0.23962 | 1.4287 | 1 | 0.93603 | 0 |
|  |  | Pyrimidine metabolism | 39 | 0.27677 | 1 | 0.24515 | 1.4059 | 1 | 0.93603 | 0 |
|  |  | Tyrosine metabolism | 42 | 0.29806 | 1 | 0.26153 | 1.3412 | 1 | 0.95514 | 0 |
|  |  | Primary bile acid biosynthesis | 46 | 0.32645 | 1 | 0.28286 | 1.2628 | 1 | 0.96803 | 0.00758 |
|  |  | Fatty acid biosynthesis | 47 | 0.33355 | 1 | 0.2881 | 1.2444 | 1 | 0.96803 | 0 |
|  |  | Purine metabolism | 65 | 0.46129 | 1 | 0.37675 | 0.97618 | 1 | 1 | 0 |
| Tai Chi v.s. Brisk Walking | Baseline v.s. 12-month visit | Arginine biosynthesis | 14 | 0.11742 | 3 | 0.0001593 | 8.7447 | 0.013382 | 0.013382 | 0 |
|  |  | D-Glutamine and D-glutamate metabolism | 6 | 0.050323 | 2 | 0.00095628 | 6.9525 | 0.079371 | 0.037496 | 0 |
|  |  | Alanine, aspartate and glutamate metabolism | 28 | 0.23484 | 3 | 0.0013391 | 6.6157 | 0.10981 | 0.037496 | 0.16186 |
|  |  | Citrate cycle (TCA cycle) | 20 | 0.16774 | 2 | 0.011335 | 4.4799 | 0.91811 | 0.23803 | 0.1049 |
|  |  | Purine metabolism | 65 | 0.54516 | 3 | 0.014896 | 4.2067 | 1 | 0.25026 | 0 |
|  |  | Glyoxylate and dicarboxylate metabolism | 32 | 0.26839 | 2 | 0.027956 | 3.5771 | 1 | 0.35542 | 0 |
|  |  | Cysteine and methionine metabolism | 33 | 0.27677 | 2 | 0.029619 | 3.5193 | 1 | 0.35542 | 0.10446 |
|  |  | Nitrogen metabolism | 6 | 0.050323 | 1 | 0.049357 | 3.0087 | 1 | 0.51825 | 0 |
|  |  | Aminoacyl-tRNA biosynthesis | 48 | 0.40258 | 2 | 0.058948 | 2.8311 | 1 | 0.54855 | 0 |
|  |  | Taurine and hypotaurine metabolism | 8 | 0.067097 | 1 | 0.065303 | 2.7287 | 1 | 0.54855 | 0.42857 |
|  |  | alpha-Linolenic acid metabolism | 13 | 0.10903 | 1 | 0.10409 | 2.2625 | 1 | 0.7949 | 0.33333 |
|  |  | Butanoate metabolism | 15 | 0.12581 | 1 | 0.11919 | 2.127 | 1 | 0.81835 | 0 |
|  |  | Histidine metabolism | 16 | 0.13419 | 1 | 0.12665 | 2.0663 | 1 | 0.81835 | 0 |
|  |  | Pyruvate metabolism | 22 | 0.18452 | 1 | 0.1702 | 1.7708 | 1 | 1 | 0.20684 |
|  |  | Glycolysis / Gluconeogenesis | 26 | 0.21806 | 1 | 0.1981 | 1.619 | 1 | 1 | 0.10044 |
|  |  | Glycine, serine and threonine metabolism | 33 | 0.27677 | 1 | 0.24487 | 1.407 | 1 | 1 | 0 |
|  |  | Biosynthesis of unsaturated fatty acids | 36 | 0.30194 | 1 | 0.26413 | 1.3313 | 1 | 1 | 0 |
|  |  | Amino sugar and nucleotide sugar metabolism | 37 | 0.31032 | 1 | 0.27045 | 1.3077 | 1 | 1 | 0 |
|  |  | Arginine and proline metabolism | 38 | 0.31871 | 1 | 0.27672 | 1.2848 | 1 | 1 | 0 |
|  |  | Pyrimidine metabolism | 39 | 0.3271 | 1 | 0.28293 | 1.2625 | 1 | 1 | 0 |
|  |  | Tyrosine metabolism | 42 | 0.35226 | 1 | 0.3013 | 1.1997 | 1 | 1 | 0 |
|  |  | Primary bile acid biosynthesis | 46 | 0.38581 | 1 | 0.3251 | 1.1236 | 1 | 1 | 0.00758 |

**Table S8 Enrichment analysis of metabolites**

| Comparisons | | Enriched pathway | total | expected | hits | Raw p | Holm p | FDR |
| --- | --- | --- | --- | --- | --- | --- | --- | --- |
| BBS | | Methionine Metabolism | 43 | 1.09 | 3 | 0.0915 | 1 | 1 |
|  |  | Phenylalanine and Tyrosine Metabolism | 28 | 0.711 | 2 | 0.157 | 1 | 1 |
|  |  | Glycine and Serine Metabolism | 59 | 1.5 | 3 | 0.185 | 1 | 1 |
|  |  | Citric Acid Cycle | 32 | 0.812 | 2 | 0.194 | 1 | 1 |
|  |  | Homocysteine Degradation | 9 | 0.229 | 1 | 0.207 | 1 | 1 |
|  |  | Gluconeogenesis | 35 | 0.889 | 2 | 0.222 | 1 | 1 |
|  |  | Fatty Acid Biosynthesis | 35 | 0.889 | 2 | 0.222 | 1 | 1 |
|  |  | Malate-Aspartate Shuttle | 10 | 0.254 | 1 | 0.228 | 1 | 1 |
|  |  | Phosphatidylethanolamine Biosynthesis | 12 | 0.305 | 1 | 0.267 | 1 | 1 |
|  |  | Glucose-Alanine Cycle | 13 | 0.33 | 1 | 0.286 | 1 | 1 |
|  |  | Phosphatidylcholine Biosynthesis | 14 | 0.355 | 1 | 0.304 | 1 | 1 |
|  |  | Beta Oxidation of Very Long Chain Fatty Acids | 17 | 0.432 | 1 | 0.356 | 1 | 1 |
|  |  | Alanine Metabolism | 17 | 0.432 | 1 | 0.356 | 1 | 1 |
|  |  | Alpha Linolenic Acid and Linoleic Acid Metabolism | 19 | 0.482 | 1 | 0.389 | 1 | 1 |
|  |  | Catecholamine Biosynthesis | 20 | 0.508 | 1 | 0.405 | 1 | 1 |
|  |  | Betaine Metabolism | 21 | 0.533 | 1 | 0.42 | 1 | 1 |
|  |  | Carnitine Synthesis | 22 | 0.559 | 1 | 0.435 | 1 | 1 |
|  |  | Warburg Effect | 58 | 1.47 | 2 | 0.439 | 1 | 1 |
|  |  | Glycerolipid Metabolism | 25 | 0.635 | 1 | 0.478 | 1 | 1 |
|  |  | Glycolysis | 25 | 0.635 | 1 | 0.478 | 1 | 1 |
|  |  | Cysteine Metabolism | 26 | 0.66 | 1 | 0.492 | 1 | 1 |
|  |  | Oxidation of Branched Chain Fatty Acids | 26 | 0.66 | 1 | 0.492 | 1 | 1 |
|  |  | Phytanic Acid Peroxisomal Oxidation | 26 | 0.66 | 1 | 0.492 | 1 | 1 |
|  |  | Mitochondrial Beta-Oxidation of Short Chain Saturated Fatty Acids | 27 | 0.686 | 1 | 0.505 | 1 | 1 |
|  |  | Pentose Phosphate Pathway | 29 | 0.736 | 1 | 0.531 | 1 | 1 |
|  |  | Urea Cycle | 29 | 0.736 | 1 | 0.531 | 1 | 1 |
|  |  | Lysine Degradation | 30 | 0.762 | 1 | 0.543 | 1 | 1 |
|  |  | Starch and Sucrose Metabolism | 31 | 0.787 | 1 | 0.555 | 1 | 1 |
|  |  | Tyrosine Metabolism | 72 | 1.83 | 2 | 0.557 | 1 | 1 |
|  |  | Ammonia Recycling | 32 | 0.812 | 1 | 0.567 | 1 | 1 |
|  |  | Fructose and Mannose Degradation | 32 | 0.812 | 1 | 0.567 | 1 | 1 |
|  |  | Beta-Alanine Metabolism | 34 | 0.863 | 1 | 0.589 | 1 | 1 |
|  |  | Aspartate Metabolism | 35 | 0.889 | 1 | 0.6 | 1 | 1 |
|  |  | Sphingolipid Metabolism | 40 | 1.02 | 1 | 0.65 | 1 | 1 |
|  |  | Propanoate Metabolism | 42 | 1.07 | 1 | 0.668 | 1 | 1 |
|  |  | Histidine Metabolism | 43 | 1.09 | 1 | 0.677 | 1 | 1 |
|  |  | Glutamate Metabolism | 49 | 1.24 | 1 | 0.725 | 1 | 1 |
|  |  | Arginine and Proline Metabolism | 53 | 1.35 | 1 | 0.753 | 1 | 1 |
|  |  | Valine, Leucine and Isoleucine Degradation | 60 | 1.52 | 1 | 0.796 | 1 | 1 |
|  |  | Tryptophan Metabolism | 60 | 1.52 | 1 | 0.796 | 1 | 1 |
| UPDRS | | Beta Oxidation of Very Long Chain Fatty Acids | 17 | 0.73 | 3 | 0.033 | 1 | 1 |
|  |  | Citric Acid Cycle | 32 | 1.38 | 4 | 0.0443 | 1 | 1 |
|  |  | Aspartate Metabolism | 35 | 1.5 | 4 | 0.0587 | 1 | 1 |
|  |  | Fatty Acid Biosynthesis | 35 | 1.5 | 4 | 0.0587 | 1 | 1 |
|  |  | Phosphatidylethanolamine Biosynthesis | 12 | 0.516 | 2 | 0.0906 | 1 | 1 |
|  |  | Phosphatidylcholine Biosynthesis | 14 | 0.602 | 2 | 0.118 | 1 | 1 |
|  |  | Alpha Linolenic Acid and Linoleic Acid Metabolism | 19 | 0.816 | 2 | 0.195 | 1 | 1 |
|  |  | Betaine Metabolism | 21 | 0.902 | 2 | 0.227 | 1 | 1 |
|  |  | Warburg Effect | 58 | 2.49 | 4 | 0.235 | 1 | 1 |
|  |  | Methionine Metabolism | 43 | 1.85 | 3 | 0.28 | 1 | 1 |
|  |  | Homocysteine Degradation | 9 | 0.387 | 1 | 0.328 | 1 | 1 |
|  |  | Lactose Degradation | 9 | 0.387 | 1 | 0.328 | 1 | 1 |
|  |  | Phenylalanine and Tyrosine Metabolism | 28 | 1.2 | 2 | 0.341 | 1 | 1 |
|  |  | Malate-Aspartate Shuttle | 10 | 0.43 | 1 | 0.357 | 1 | 1 |
|  |  | Urea Cycle | 29 | 1.25 | 2 | 0.357 | 1 | 1 |
|  |  | Starch and Sucrose Metabolism | 31 | 1.33 | 2 | 0.389 | 1 | 1 |
|  |  | Taurine and Hypotaurine Metabolism | 12 | 0.516 | 1 | 0.411 | 1 | 1 |
|  |  | Beta-Alanine Metabolism | 34 | 1.46 | 2 | 0.435 | 1 | 1 |
|  |  | Glucose-Alanine Cycle | 13 | 0.559 | 1 | 0.437 | 1 | 1 |
|  |  | Gluconeogenesis | 35 | 1.5 | 2 | 0.45 | 1 | 1 |
|  |  | Glycine and Serine Metabolism | 59 | 2.54 | 3 | 0.472 | 1 | 1 |
|  |  | Alanine Metabolism | 17 | 0.73 | 1 | 0.529 | 1 | 1 |
|  |  | Propanoate Metabolism | 42 | 1.8 | 2 | 0.549 | 1 | 1 |
|  |  | Histidine Metabolism | 43 | 1.85 | 2 | 0.562 | 1 | 1 |
|  |  | Mitochondrial Electron Transport Chain | 19 | 0.816 | 1 | 0.569 | 1 | 1 |
|  |  | Catecholamine Biosynthesis | 20 | 0.859 | 1 | 0.588 | 1 | 1 |
|  |  | Lactose Synthesis | 20 | 0.859 | 1 | 0.588 | 1 | 1 |
|  |  | Carnitine Synthesis | 22 | 0.945 | 1 | 0.623 | 1 | 1 |
|  |  | Transfer of Acetyl Groups into Mitochondria | 22 | 0.945 | 1 | 0.623 | 1 | 1 |
|  |  | Glycerolipid Metabolism | 25 | 1.07 | 1 | 0.671 | 1 | 1 |
|  |  | Glycolysis | 25 | 1.07 | 1 | 0.671 | 1 | 1 |
|  |  | Arginine and Proline Metabolism | 53 | 2.28 | 2 | 0.678 | 1 | 1 |
|  |  | Cysteine Metabolism | 26 | 1.12 | 1 | 0.685 | 1 | 1 |
|  |  | Oxidation of Branched Chain Fatty Acids | 26 | 1.12 | 1 | 0.685 | 1 | 1 |
|  |  | Phytanic Acid Peroxisomal Oxidation | 26 | 1.12 | 1 | 0.685 | 1 | 1 |
|  |  | Mitochondrial Beta-Oxidation of Short Chain Saturated Fatty Acids | 27 | 1.16 | 1 | 0.699 | 1 | 1 |
|  |  | Selenoamino Acid Metabolism | 28 | 1.2 | 1 | 0.713 | 1 | 1 |
|  |  | Phospholipid Biosynthesis | 29 | 1.25 | 1 | 0.725 | 1 | 1 |
|  |  | Pentose Phosphate Pathway | 29 | 1.25 | 1 | 0.725 | 1 | 1 |
|  |  | Lysine Degradation | 30 | 1.29 | 1 | 0.737 | 1 | 1 |
|  |  | Valine, Leucine and Isoleucine Degradation | 60 | 2.58 | 2 | 0.744 | 1 | 1 |
|  |  | Tryptophan Metabolism | 60 | 2.58 | 2 | 0.744 | 1 | 1 |
|  |  | Ammonia Recycling | 32 | 1.38 | 1 | 0.76 | 1 | 1 |
|  |  | Fructose and Mannose Degradation | 32 | 1.38 | 1 | 0.76 | 1 | 1 |
|  |  | Galactose Metabolism | 38 | 1.63 | 1 | 0.817 | 1 | 1 |
|  |  | Tyrosine Metabolism | 72 | 3.09 | 2 | 0.831 | 1 | 1 |
|  |  | Sphingolipid Metabolism | 40 | 1.72 | 1 | 0.833 | 1 | 1 |
|  |  | Purine Metabolism | 74 | 3.18 | 2 | 0.843 | 1 | 1 |
|  |  | Pyruvate Metabolism | 48 | 2.06 | 1 | 0.885 | 1 | 1 |
|  |  | Glutamate Metabolism | 49 | 2.11 | 1 | 0.89 | 1 | 1 |
|  |  | Pyrimidine Metabolism | 59 | 2.54 | 1 | 0.931 | 1 | 1 |
|  |  | Bile Acid Biosynthesis | 65 | 2.79 | 1 | 0.948 | 1 | 1 |
|  |  | Arachidonic Acid Metabolism | 69 | 2.96 | 1 | 0.957 | 1 | 1 |
| UPDRS Part III | | Beta Oxidation of Very Long Chain Fatty Acids | 17 | 0.73 | 3 | 0.033 | 1 | 1 |
|  |  | Fatty Acid Biosynthesis | 35 | 1.5 | 4 | 0.0587 | 1 | 1 |
|  |  | Phosphatidylethanolamine Biosynthesis | 12 | 0.516 | 2 | 0.0906 | 1 | 1 |
|  |  | Phosphatidylcholine Biosynthesis | 14 | 0.602 | 2 | 0.118 | 1 | 1 |
|  |  | Starch and Sucrose Metabolism | 31 | 1.33 | 3 | 0.144 | 1 | 1 |
|  |  | Citric Acid Cycle | 32 | 1.38 | 3 | 0.155 | 1 | 1 |
|  |  | Aspartate Metabolism | 35 | 1.5 | 3 | 0.187 | 1 | 1 |
|  |  | Betaine Metabolism | 21 | 0.902 | 2 | 0.227 | 1 | 1 |
|  |  | Methionine Metabolism | 43 | 1.85 | 3 | 0.28 | 1 | 1 |
|  |  | Homocysteine Degradation | 9 | 0.387 | 1 | 0.328 | 1 | 1 |
|  |  | Lactose Degradation | 9 | 0.387 | 1 | 0.328 | 1 | 1 |
|  |  | Phenylalanine and Tyrosine Metabolism | 28 | 1.2 | 2 | 0.341 | 1 | 1 |
|  |  | Malate-Aspartate Shuttle | 10 | 0.43 | 1 | 0.357 | 1 | 1 |
|  |  | Taurine and Hypotaurine Metabolism | 12 | 0.516 | 1 | 0.411 | 1 | 1 |
|  |  | Beta-Alanine Metabolism | 34 | 1.46 | 2 | 0.435 | 1 | 1 |
|  |  | Glucose-Alanine Cycle | 13 | 0.559 | 1 | 0.437 | 1 | 1 |
|  |  | Gluconeogenesis | 35 | 1.5 | 2 | 0.45 | 1 | 1 |
|  |  | Warburg Effect | 58 | 2.49 | 3 | 0.46 | 1 | 1 |
|  |  | Glycine and Serine Metabolism | 59 | 2.54 | 3 | 0.472 | 1 | 1 |
|  |  | Galactose Metabolism | 38 | 1.63 | 2 | 0.494 | 1 | 1 |
|  |  | Alanine Metabolism | 17 | 0.73 | 1 | 0.529 | 1 | 1 |
|  |  | Propanoate Metabolism | 42 | 1.8 | 2 | 0.549 | 1 | 1 |
|  |  | Histidine Metabolism | 43 | 1.85 | 2 | 0.562 | 1 | 1 |
|  |  | Alpha Linolenic Acid and Linoleic Acid Metabolism | 19 | 0.816 | 1 | 0.569 | 1 | 1 |
|  |  | Catecholamine Biosynthesis | 20 | 0.859 | 1 | 0.588 | 1 | 1 |
|  |  | Lactose Synthesis | 20 | 0.859 | 1 | 0.588 | 1 | 1 |
|  |  | Carnitine Synthesis | 22 | 0.945 | 1 | 0.623 | 1 | 1 |
|  |  | Transfer of Acetyl Groups into Mitochondria | 22 | 0.945 | 1 | 0.623 | 1 | 1 |
|  |  | Glycerolipid Metabolism | 25 | 1.07 | 1 | 0.671 | 1 | 1 |
|  |  | Glycolysis | 25 | 1.07 | 1 | 0.671 | 1 | 1 |
|  |  | Cysteine Metabolism | 26 | 1.12 | 1 | 0.685 | 1 | 1 |
|  |  | Oxidation of Branched Chain Fatty Acids | 26 | 1.12 | 1 | 0.685 | 1 | 1 |
|  |  | Phytanic Acid Peroxisomal Oxidation | 26 | 1.12 | 1 | 0.685 | 1 | 1 |
|  |  | Mitochondrial Beta-Oxidation of Short Chain Saturated Fatty Acids | 27 | 1.16 | 1 | 0.699 | 1 | 1 |
|  |  | Selenoamino Acid Metabolism | 28 | 1.2 | 1 | 0.713 | 1 | 1 |
|  |  | Phospholipid Biosynthesis | 29 | 1.25 | 1 | 0.725 | 1 | 1 |
|  |  | Pentose Phosphate Pathway | 29 | 1.25 | 1 | 0.725 | 1 | 1 |
|  |  | Urea Cycle | 29 | 1.25 | 1 | 0.725 | 1 | 1 |
|  |  | Lysine Degradation | 30 | 1.29 | 1 | 0.737 | 1 | 1 |
|  |  | Valine, Leucine and Isoleucine Degradation | 60 | 2.58 | 2 | 0.744 | 1 | 1 |
|  |  | Tryptophan Metabolism | 60 | 2.58 | 2 | 0.744 | 1 | 1 |
|  |  | Ammonia Recycling | 32 | 1.38 | 1 | 0.76 | 1 | 1 |
|  |  | Fructose and Mannose Degradation | 32 | 1.38 | 1 | 0.76 | 1 | 1 |
|  |  | Tyrosine Metabolism | 72 | 3.09 | 2 | 0.831 | 1 | 1 |
|  |  | Sphingolipid Metabolism | 40 | 1.72 | 1 | 0.833 | 1 | 1 |
|  |  | Pyruvate Metabolism | 48 | 2.06 | 1 | 0.885 | 1 | 1 |
|  |  | Glutamate Metabolism | 49 | 2.11 | 1 | 0.89 | 1 | 1 |
|  |  | Arginine and Proline Metabolism | 53 | 2.28 | 1 | 0.908 | 1 | 1 |
|  |  | Pyrimidine Metabolism | 59 | 2.54 | 1 | 0.931 | 1 | 1 |
|  |  | Bile Acid Biosynthesis | 65 | 2.79 | 1 | 0.948 | 1 | 1 |
|  |  | Arachidonic Acid Metabolism | 69 | 2.96 | 1 | 0.957 | 1 | 1 |
|  |  | Purine Metabolism | 74 | 3.18 | 1 | 0.966 | 1 | 1 |
| TUG | | Homocysteine Degradation | 9 | 0.158 | 1 | 0.148 | 1 | 1 |
|  |  | Methionine Metabolism | 43 | 0.756 | 2 | 0.173 | 1 | 1 |
|  |  | Glycine and Serine Metabolism | 59 | 1.04 | 2 | 0.278 | 1 | 1 |
|  |  | Alpha Linolenic Acid and Linoleic Acid Metabolism | 19 | 0.334 | 1 | 0.288 | 1 | 1 |
|  |  | Catecholamine Biosynthesis | 20 | 0.352 | 1 | 0.301 | 1 | 1 |
|  |  | Betaine Metabolism | 21 | 0.369 | 1 | 0.313 | 1 | 1 |
|  |  | Glycerolipid Metabolism | 25 | 0.439 | 1 | 0.362 | 1 | 1 |
|  |  | Glycolysis | 25 | 0.439 | 1 | 0.362 | 1 | 1 |
|  |  | Pentose Phosphate Pathway | 29 | 0.51 | 1 | 0.406 | 1 | 1 |
|  |  | Starch and Sucrose Metabolism | 31 | 0.545 | 1 | 0.428 | 1 | 1 |
|  |  | Citric Acid Cycle | 32 | 0.562 | 1 | 0.438 | 1 | 1 |
|  |  | Fructose and Mannose Degradation | 32 | 0.562 | 1 | 0.438 | 1 | 1 |
|  |  | Gluconeogenesis | 35 | 0.615 | 1 | 0.468 | 1 | 1 |
|  |  | Histidine Metabolism | 43 | 0.756 | 1 | 0.541 | 1 | 1 |
|  |  | Warburg Effect | 58 | 1.02 | 1 | 0.653 | 1 | 1 |
|  |  | Tryptophan Metabolism | 60 | 1.05 | 1 | 0.666 | 1 | 1 |
| Tai Chi v.s. Control | Baseline v.s. 6-month visit | Urea Cycle | 29 | 0.453 | 3 | 0.00893 | 0.875 | 0.875 |
|  |  | Ammonia Recycling | 32 | 0.5 | 2 | 0.0865 | 1 | 1 |
|  |  | Amino Sugar Metabolism | 33 | 0.516 | 2 | 0.0912 | 1 | 1 |
|  |  | De Novo Triacylglycerol Biosynthesis | 9 | 0.141 | 1 | 0.133 | 1 | 1 |
|  |  | Malate-Aspartate Shuttle | 10 | 0.156 | 1 | 0.146 | 1 | 1 |
|  |  | Pyruvaldehyde Degradation | 10 | 0.156 | 1 | 0.146 | 1 | 1 |
|  |  | D-Arginine and D-Ornithine Metabolism | 11 | 0.172 | 1 | 0.16 | 1 | 1 |
|  |  | Glycerol Phosphate Shuttle | 11 | 0.172 | 1 | 0.16 | 1 | 1 |
|  |  | Cardiolipin Biosynthesis | 11 | 0.172 | 1 | 0.16 | 1 | 1 |
|  |  | Glutamate Metabolism | 49 | 0.766 | 2 | 0.176 | 1 | 1 |
|  |  | Glucose-Alanine Cycle | 13 | 0.203 | 1 | 0.186 | 1 | 1 |
|  |  | Arginine and Proline Metabolism | 53 | 0.828 | 2 | 0.199 | 1 | 1 |
|  |  | Glycine and Serine Metabolism | 59 | 0.922 | 2 | 0.234 | 1 | 1 |
|  |  | Beta Oxidation of Very Long Chain Fatty Acids | 17 | 0.266 | 1 | 0.237 | 1 | 1 |
|  |  | Alanine Metabolism | 17 | 0.266 | 1 | 0.237 | 1 | 1 |
|  |  | Spermidine and Spermine Biosynthesis | 18 | 0.281 | 1 | 0.249 | 1 | 1 |
|  |  | Alpha Linolenic Acid and Linoleic Acid Metabolism | 19 | 0.297 | 1 | 0.261 | 1 | 1 |
|  |  | Mitochondrial Electron Transport Chain | 19 | 0.297 | 1 | 0.261 | 1 | 1 |
|  |  | Betaine Metabolism | 21 | 0.328 | 1 | 0.284 | 1 | 1 |
|  |  | Transfer of Acetyl Groups into Mitochondria | 22 | 0.344 | 1 | 0.295 | 1 | 1 |
|  |  | Glycerolipid Metabolism | 25 | 0.391 | 1 | 0.329 | 1 | 1 |
|  |  | Glycolysis | 25 | 0.391 | 1 | 0.329 | 1 | 1 |
|  |  | Cysteine Metabolism | 26 | 0.406 | 1 | 0.339 | 1 | 1 |
|  |  | Mitochondrial Beta-Oxidation of Medium Chain Saturated Fatty Acids | 27 | 0.422 | 1 | 0.35 | 1 | 1 |
|  |  | Phospholipid Biosynthesis | 29 | 0.453 | 1 | 0.371 | 1 | 1 |
|  |  | Starch and Sucrose Metabolism | 31 | 0.484 | 1 | 0.391 | 1 | 1 |
|  |  | Citric Acid Cycle | 32 | 0.5 | 1 | 0.401 | 1 | 1 |
|  |  | Fructose and Mannose Degradation | 32 | 0.5 | 1 | 0.401 | 1 | 1 |
|  |  | Beta-Alanine Metabolism | 34 | 0.531 | 1 | 0.42 | 1 | 1 |
|  |  | Aspartate Metabolism | 35 | 0.547 | 1 | 0.429 | 1 | 1 |
|  |  | Gluconeogenesis | 35 | 0.547 | 1 | 0.429 | 1 | 1 |
|  |  | Fatty Acid Biosynthesis | 35 | 0.547 | 1 | 0.429 | 1 | 1 |
|  |  | Galactose Metabolism | 38 | 0.594 | 1 | 0.456 | 1 | 1 |
|  |  | Methionine Metabolism | 43 | 0.672 | 1 | 0.499 | 1 | 1 |
|  |  | Pyruvate Metabolism | 48 | 0.75 | 1 | 0.539 | 1 | 1 |
|  |  | Warburg Effect | 58 | 0.906 | 1 | 0.609 | 1 | 1 |
|  |  | Valine, Leucine and Isoleucine Degradation | 60 | 0.938 | 1 | 0.622 | 1 | 1 |
|  |  | Tyrosine Metabolism | 72 | 1.12 | 1 | 0.691 | 1 | 1 |
|  |  | Purine Metabolism | 74 | 1.16 | 1 | 0.702 | 1 | 1 |
| Tai Chi v.s. Control | 6-month visit v.s. 12-month visit | Mitochondrial Electron Transport Chain | 19 | 0.334 | 3 | 0.00371 | 0.364 | 0.307 |
|  |  | Phenylalanine and Tyrosine Metabolism | 28 | 0.492 | 3 | 0.0114 | 1 | 0.307 |
|  |  | Arginine and Proline Metabolism | 53 | 0.932 | 4 | 0.0114 | 1 | 0.307 |
|  |  | Urea Cycle | 29 | 0.51 | 3 | 0.0125 | 1 | 0.307 |
|  |  | Citric Acid Cycle | 32 | 0.562 | 3 | 0.0164 | 1 | 0.322 |
|  |  | Purine Metabolism | 74 | 1.3 | 4 | 0.0354 | 1 | 0.578 |
|  |  | Carnitine Synthesis | 22 | 0.387 | 2 | 0.0548 | 1 | 0.752 |
|  |  | Oxidation of Branched Chain Fatty Acids | 26 | 0.457 | 2 | 0.0739 | 1 | 0.752 |
|  |  | Phytanic Acid Peroxisomal Oxidation | 26 | 0.457 | 2 | 0.0739 | 1 | 0.752 |
|  |  | Warburg Effect | 58 | 1.02 | 3 | 0.0768 | 1 | 0.752 |
|  |  | Aspartate Metabolism | 35 | 0.615 | 2 | 0.123 | 1 | 1 |
|  |  | Tyrosine Metabolism | 72 | 1.27 | 3 | 0.127 | 1 | 1 |
|  |  | De Novo Triacylglycerol Biosynthesis | 9 | 0.158 | 1 | 0.148 | 1 | 1 |
|  |  | Malate-Aspartate Shuttle | 10 | 0.176 | 1 | 0.163 | 1 | 1 |
|  |  | D-Arginine and D-Ornithine Metabolism | 11 | 0.193 | 1 | 0.178 | 1 | 1 |
|  |  | Glycerol Phosphate Shuttle | 11 | 0.193 | 1 | 0.178 | 1 | 1 |
|  |  | Cardiolipin Biosynthesis | 11 | 0.193 | 1 | 0.178 | 1 | 1 |
|  |  | Ketone Body Metabolism | 13 | 0.229 | 1 | 0.207 | 1 | 1 |
|  |  | Glucose-Alanine Cycle | 13 | 0.229 | 1 | 0.207 | 1 | 1 |
|  |  | Glutamate Metabolism | 49 | 0.861 | 2 | 0.211 | 1 | 1 |
|  |  | Alanine Metabolism | 17 | 0.299 | 1 | 0.262 | 1 | 1 |
|  |  | Valine, Leucine and Isoleucine Degradation | 60 | 1.05 | 2 | 0.285 | 1 | 1 |
|  |  | Butyrate Metabolism | 19 | 0.334 | 1 | 0.288 | 1 | 1 |
|  |  | Betaine Metabolism | 21 | 0.369 | 1 | 0.313 | 1 | 1 |
|  |  | Glycerolipid Metabolism | 25 | 0.439 | 1 | 0.362 | 1 | 1 |
|  |  | Cysteine Metabolism | 26 | 0.457 | 1 | 0.373 | 1 | 1 |
|  |  | Selenoamino Acid Metabolism | 28 | 0.492 | 1 | 0.395 | 1 | 1 |
|  |  | Phospholipid Biosynthesis | 29 | 0.51 | 1 | 0.406 | 1 | 1 |
|  |  | Lysine Degradation | 30 | 0.527 | 1 | 0.417 | 1 | 1 |
|  |  | Ammonia Recycling | 32 | 0.562 | 1 | 0.438 | 1 | 1 |
|  |  | Fructose and Mannose Degradation | 32 | 0.562 | 1 | 0.438 | 1 | 1 |
|  |  | Beta-Alanine Metabolism | 34 | 0.598 | 1 | 0.458 | 1 | 1 |
|  |  | Gluconeogenesis | 35 | 0.615 | 1 | 0.468 | 1 | 1 |
|  |  | Galactose Metabolism | 38 | 0.668 | 1 | 0.497 | 1 | 1 |
|  |  | Propanoate Metabolism | 42 | 0.738 | 1 | 0.532 | 1 | 1 |
|  |  | Methionine Metabolism | 43 | 0.756 | 1 | 0.541 | 1 | 1 |
|  |  | Histidine Metabolism | 43 | 0.756 | 1 | 0.541 | 1 | 1 |
|  |  | Glycine and Serine Metabolism | 59 | 1.04 | 1 | 0.66 | 1 | 1 |
|  |  | Tryptophan Metabolism | 60 | 1.05 | 1 | 0.666 | 1 | 1 |
| Tai Chi v.s. Control | Baseline v.s. 12-month visit | Betaine Metabolism | 21 | 0.349 | 2 | 0.0453 | 1 | 1 |
|  |  | Methylhistidine Metabolism | 4 | 0.0664 | 1 | 0.0649 | 1 | 1 |
|  |  | Urea Cycle | 29 | 0.481 | 2 | 0.081 | 1 | 1 |
|  |  | Starch and Sucrose Metabolism | 31 | 0.515 | 2 | 0.091 | 1 | 1 |
|  |  | Purine Metabolism | 74 | 1.23 | 3 | 0.119 | 1 | 1 |
|  |  | Methionine Metabolism | 43 | 0.714 | 2 | 0.157 | 1 | 1 |
|  |  | D-Arginine and D-Ornithine Metabolism | 11 | 0.183 | 1 | 0.169 | 1 | 1 |
|  |  | Arginine and Proline Metabolism | 53 | 0.88 | 2 | 0.218 | 1 | 1 |
|  |  | Beta Oxidation of Very Long Chain Fatty Acids | 17 | 0.282 | 1 | 0.249 | 1 | 1 |
|  |  | Warburg Effect | 58 | 0.963 | 2 | 0.25 | 1 | 1 |
|  |  | Glycine and Serine Metabolism | 59 | 0.979 | 2 | 0.256 | 1 | 1 |
|  |  | Spermidine and Spermine Biosynthesis | 18 | 0.299 | 1 | 0.262 | 1 | 1 |
|  |  | Mitochondrial Electron Transport Chain | 19 | 0.315 | 1 | 0.275 | 1 | 1 |
|  |  | Glycerolipid Metabolism | 25 | 0.415 | 1 | 0.345 | 1 | 1 |
|  |  | Glycolysis | 25 | 0.415 | 1 | 0.345 | 1 | 1 |
|  |  | Mitochondrial Beta-Oxidation of Short Chain Saturated Fatty Acids | 27 | 0.448 | 1 | 0.367 | 1 | 1 |
|  |  | Phenylalanine and Tyrosine Metabolism | 28 | 0.465 | 1 | 0.378 | 1 | 1 |
|  |  | Selenoamino Acid Metabolism | 28 | 0.465 | 1 | 0.378 | 1 | 1 |
|  |  | Ammonia Recycling | 32 | 0.531 | 1 | 0.42 | 1 | 1 |
|  |  | Citric Acid Cycle | 32 | 0.531 | 1 | 0.42 | 1 | 1 |
|  |  | Fructose and Mannose Degradation | 32 | 0.531 | 1 | 0.42 | 1 | 1 |
|  |  | Amino Sugar Metabolism | 33 | 0.548 | 1 | 0.43 | 1 | 1 |
|  |  | Beta-Alanine Metabolism | 34 | 0.564 | 1 | 0.439 | 1 | 1 |
|  |  | Aspartate Metabolism | 35 | 0.581 | 1 | 0.449 | 1 | 1 |
|  |  | Gluconeogenesis | 35 | 0.581 | 1 | 0.449 | 1 | 1 |
|  |  | Fatty Acid Biosynthesis | 35 | 0.581 | 1 | 0.449 | 1 | 1 |
|  |  | Galactose Metabolism | 38 | 0.631 | 1 | 0.477 | 1 | 1 |
|  |  | Histidine Metabolism | 43 | 0.714 | 1 | 0.521 | 1 | 1 |
|  |  | Tyrosine Metabolism | 72 | 1.2 | 1 | 0.713 | 1 | 1 |
| Brisk Walking v.s. Control | Baseline v.s. 6-month visit | Urea Cycle | 29 | 0.312 | 4 | 0.00015 | 0.0147 | 0.0147 |
|  |  | Ammonia Recycling | 32 | 0.344 | 3 | 0.00386 | 0.375 | 0.147 |
|  |  | Malate-Aspartate Shuttle | 10 | 0.107 | 2 | 0.00451 | 0.433 | 0.147 |
|  |  | Glucose-Alanine Cycle | 13 | 0.14 | 2 | 0.00768 | 0.729 | 0.188 |
|  |  | Glutamate Metabolism | 49 | 0.526 | 3 | 0.013 | 1 | 0.214 |
|  |  | Alanine Metabolism | 17 | 0.183 | 2 | 0.0131 | 1 | 0.214 |
|  |  | Arginine and Proline Metabolism | 53 | 0.569 | 3 | 0.0161 | 1 | 0.225 |
|  |  | Glycine and Serine Metabolism | 59 | 0.634 | 3 | 0.0215 | 1 | 0.264 |
|  |  | Cysteine Metabolism | 26 | 0.279 | 2 | 0.0296 | 1 | 0.323 |
|  |  | Purine Metabolism | 74 | 0.795 | 3 | 0.0392 | 1 | 0.384 |
|  |  | Amino Sugar Metabolism | 33 | 0.354 | 2 | 0.0462 | 1 | 0.388 |
|  |  | Beta-Alanine Metabolism | 34 | 0.365 | 2 | 0.0488 | 1 | 0.388 |
|  |  | Aspartate Metabolism | 35 | 0.376 | 2 | 0.0515 | 1 | 0.388 |
|  |  | Lactose Degradation | 9 | 0.0967 | 1 | 0.093 | 1 | 0.651 |
|  |  | Pyruvaldehyde Degradation | 10 | 0.107 | 1 | 0.103 | 1 | 0.672 |
|  |  | D-Arginine and D-Ornithine Metabolism | 11 | 0.118 | 1 | 0.113 | 1 | 0.689 |
|  |  | Warburg Effect | 58 | 0.623 | 2 | 0.125 | 1 | 0.72 |
|  |  | Valine, Leucine and Isoleucine Degradation | 60 | 0.645 | 2 | 0.132 | 1 | 0.72 |
|  |  | Tyrosine Metabolism | 72 | 0.773 | 2 | 0.178 | 1 | 0.836 |
|  |  | Spermidine and Spermine Biosynthesis | 18 | 0.193 | 1 | 0.178 | 1 | 0.836 |
|  |  | Alpha Linolenic Acid and Linoleic Acid Metabolism | 19 | 0.204 | 1 | 0.187 | 1 | 0.836 |
|  |  | Lactose Synthesis | 20 | 0.215 | 1 | 0.196 | 1 | 0.836 |
|  |  | Glutathione Metabolism | 21 | 0.226 | 1 | 0.205 | 1 | 0.836 |
|  |  | Betaine Metabolism | 21 | 0.226 | 1 | 0.205 | 1 | 0.836 |
|  |  | Transfer of Acetyl Groups into Mitochondria | 22 | 0.236 | 1 | 0.213 | 1 | 0.837 |
|  |  | Glycolysis | 25 | 0.269 | 1 | 0.239 | 1 | 0.901 |
|  |  | Phenylalanine and Tyrosine Metabolism | 28 | 0.301 | 1 | 0.264 | 1 | 0.947 |
|  |  | Folate Metabolism | 29 | 0.312 | 1 | 0.272 | 1 | 0.947 |
|  |  | Lysine Degradation | 30 | 0.322 | 1 | 0.28 | 1 | 0.947 |
|  |  | Citric Acid Cycle | 32 | 0.344 | 1 | 0.296 | 1 | 0.967 |
|  |  | Gluconeogenesis | 35 | 0.376 | 1 | 0.319 | 1 | 1 |
|  |  | Nicotinate and Nicotinamide Metabolism | 37 | 0.397 | 1 | 0.334 | 1 | 1 |
|  |  | Galactose Metabolism | 38 | 0.408 | 1 | 0.342 | 1 | 1 |
|  |  | Propanoate Metabolism | 42 | 0.451 | 1 | 0.371 | 1 | 1 |
|  |  | Methionine Metabolism | 43 | 0.462 | 1 | 0.378 | 1 | 1 |
|  |  | Histidine Metabolism | 43 | 0.462 | 1 | 0.378 | 1 | 1 |
|  |  | Pyruvate Metabolism | 48 | 0.516 | 1 | 0.412 | 1 | 1 |
|  |  | Tryptophan Metabolism | 60 | 0.645 | 1 | 0.487 | 1 | 1 |
|  |  | Arachidonic Acid Metabolism | 69 | 0.741 | 1 | 0.538 | 1 | 1 |
| Brisk Walking v.s. control | 6-month visit v.s. 12-month visit | Urea Cycle | 29 | 0.425 | 4 | 0.000573 | 0.0561 | 0.0327 |
|  |  | Glucose-Alanine Cycle | 13 | 0.19 | 3 | 0.000667 | 0.0647 | 0.0327 |
|  |  | Alanine Metabolism | 17 | 0.249 | 2 | 0.024 | 1 | 0.693 |
|  |  | Mitochondrial Electron Transport Chain | 19 | 0.278 | 2 | 0.0297 | 1 | 0.693 |
|  |  | Transfer of Acetyl Groups into Mitochondria | 22 | 0.322 | 2 | 0.0391 | 1 | 0.693 |
|  |  | Warburg Effect | 58 | 0.85 | 3 | 0.0483 | 1 | 0.693 |
|  |  | Glycolysis | 25 | 0.366 | 2 | 0.0495 | 1 | 0.693 |
|  |  | Ammonia Recycling | 32 | 0.469 | 2 | 0.0771 | 1 | 0.804 |
|  |  | Citric Acid Cycle | 32 | 0.469 | 2 | 0.0771 | 1 | 0.804 |
|  |  | Aspartate Metabolism | 35 | 0.513 | 2 | 0.0902 | 1 | 0.804 |
|  |  | Gluconeogenesis | 35 | 0.513 | 2 | 0.0902 | 1 | 0.804 |
|  |  | Lactose Degradation | 9 | 0.132 | 1 | 0.125 | 1 | 0.863 |
|  |  | De Novo Triacylglycerol Biosynthesis | 9 | 0.132 | 1 | 0.125 | 1 | 0.863 |
|  |  | Pyruvaldehyde Degradation | 10 | 0.146 | 1 | 0.138 | 1 | 0.863 |
|  |  | D-Arginine and D-Ornithine Metabolism | 11 | 0.161 | 1 | 0.151 | 1 | 0.863 |
|  |  | Glycerol Phosphate Shuttle | 11 | 0.161 | 1 | 0.151 | 1 | 0.863 |
|  |  | Cardiolipin Biosynthesis | 11 | 0.161 | 1 | 0.151 | 1 | 0.863 |
|  |  | Glutamate Metabolism | 49 | 0.718 | 2 | 0.159 | 1 | 0.863 |
|  |  | Arginine and Proline Metabolism | 53 | 0.776 | 2 | 0.18 | 1 | 0.927 |
|  |  | Glycine and Serine Metabolism | 59 | 0.864 | 2 | 0.212 | 1 | 1 |
|  |  | Lactose Synthesis | 20 | 0.293 | 1 | 0.258 | 1 | 1 |
|  |  | Glutathione Metabolism | 21 | 0.308 | 1 | 0.269 | 1 | 1 |
|  |  | Purine Metabolism | 74 | 1.08 | 2 | 0.296 | 1 | 1 |
|  |  | Glycerolipid Metabolism | 25 | 0.366 | 1 | 0.312 | 1 | 1 |
|  |  | Cysteine Metabolism | 26 | 0.381 | 1 | 0.322 | 1 | 1 |
|  |  | Phenylalanine and Tyrosine Metabolism | 28 | 0.41 | 1 | 0.342 | 1 | 1 |
|  |  | Selenoamino Acid Metabolism | 28 | 0.41 | 1 | 0.342 | 1 | 1 |
|  |  | Phospholipid Biosynthesis | 29 | 0.425 | 1 | 0.352 | 1 | 1 |
|  |  | Pentose Phosphate Pathway | 29 | 0.425 | 1 | 0.352 | 1 | 1 |
|  |  | Amino Sugar Metabolism | 33 | 0.483 | 1 | 0.39 | 1 | 1 |
|  |  | Galactose Metabolism | 38 | 0.557 | 1 | 0.435 | 1 | 1 |
|  |  | Sphingolipid Metabolism | 40 | 0.586 | 1 | 0.452 | 1 | 1 |
|  |  | Pyruvate Metabolism | 48 | 0.703 | 1 | 0.516 | 1 | 1 |
|  |  | Tryptophan Metabolism | 60 | 0.879 | 1 | 0.598 | 1 | 1 |
|  |  | Tyrosine Metabolism | 72 | 1.05 | 1 | 0.668 | 1 | 1 |
| Brisk Walking v.s. Control | Baseline v.s. 12-month visit | Taurine and Hypotaurine Metabolism | 12 | 0.152 | 2 | 0.00915 | 0.896 | 0.617 |
|  |  | Methionine Metabolism | 43 | 0.546 | 3 | 0.0147 | 1 | 0.617 |
|  |  | Glutamate Metabolism | 49 | 0.622 | 3 | 0.021 | 1 | 0.617 |
|  |  | Betaine Metabolism | 21 | 0.267 | 2 | 0.0273 | 1 | 0.617 |
|  |  | Glycine and Serine Metabolism | 59 | 0.749 | 3 | 0.0344 | 1 | 0.617 |
|  |  | Cysteine Metabolism | 26 | 0.33 | 2 | 0.0407 | 1 | 0.617 |
|  |  | Urea Cycle | 29 | 0.368 | 2 | 0.0498 | 1 | 0.617 |
|  |  | Ammonia Recycling | 32 | 0.406 | 2 | 0.0596 | 1 | 0.617 |
|  |  | Purine Metabolism | 74 | 0.939 | 3 | 0.0613 | 1 | 0.617 |
|  |  | Amino Sugar Metabolism | 33 | 0.419 | 2 | 0.0629 | 1 | 0.617 |
|  |  | Phenylacetate Metabolism | 9 | 0.114 | 1 | 0.109 | 1 | 0.89 |
|  |  | Homocysteine Degradation | 9 | 0.114 | 1 | 0.109 | 1 | 0.89 |
|  |  | Pyruvaldehyde Degradation | 10 | 0.127 | 1 | 0.12 | 1 | 0.908 |
|  |  | Glucose-Alanine Cycle | 13 | 0.165 | 1 | 0.154 | 1 | 1 |
|  |  | Warburg Effect | 58 | 0.736 | 2 | 0.165 | 1 | 1 |
|  |  | Beta Oxidation of Very Long Chain Fatty Acids | 17 | 0.216 | 1 | 0.197 | 1 | 1 |
|  |  | Alanine Metabolism | 17 | 0.216 | 1 | 0.197 | 1 | 1 |
|  |  | Spermidine and Spermine Biosynthesis | 18 | 0.229 | 1 | 0.207 | 1 | 1 |
|  |  | Glutathione Metabolism | 21 | 0.267 | 1 | 0.237 | 1 | 1 |
|  |  | Pantothenate and CoA Biosynthesis | 21 | 0.267 | 1 | 0.237 | 1 | 1 |
|  |  | Transfer of Acetyl Groups into Mitochondria | 22 | 0.279 | 1 | 0.247 | 1 | 1 |
|  |  | Glycolysis | 25 | 0.317 | 1 | 0.276 | 1 | 1 |
|  |  | Mitochondrial Beta-Oxidation of Medium Chain Saturated Fatty Acids | 27 | 0.343 | 1 | 0.295 | 1 | 1 |
|  |  | Selenoamino Acid Metabolism | 28 | 0.355 | 1 | 0.304 | 1 | 1 |
|  |  | Citric Acid Cycle | 32 | 0.406 | 1 | 0.34 | 1 | 1 |
|  |  | Aspartate Metabolism | 35 | 0.444 | 1 | 0.365 | 1 | 1 |
|  |  | Gluconeogenesis | 35 | 0.444 | 1 | 0.365 | 1 | 1 |
|  |  | Fatty Acid Biosynthesis | 35 | 0.444 | 1 | 0.365 | 1 | 1 |
|  |  | Nicotinate and Nicotinamide Metabolism | 37 | 0.47 | 1 | 0.382 | 1 | 1 |
|  |  | Pyruvate Metabolism | 48 | 0.609 | 1 | 0.466 | 1 | 1 |
|  |  | Pyrimidine Metabolism | 59 | 0.749 | 1 | 0.54 | 1 | 1 |
|  |  | Bile Acid Biosynthesis | 65 | 0.825 | 1 | 0.576 | 1 | 1 |
| Tai Chi v.s. Brisk Walking | Baseline v.s. 6-month visit | Alpha Linolenic Acid and Linoleic Acid Metabolism | 19 | 0.241 | 2 | 0.0225 | 1 | 1 |
|  |  | Urea Cycle | 29 | 0.368 | 2 | 0.0498 | 1 | 1 |
|  |  | Phenylacetate Metabolism | 9 | 0.114 | 1 | 0.109 | 1 | 1 |
|  |  | D-Arginine and D-Ornithine Metabolism | 11 | 0.14 | 1 | 0.132 | 1 | 1 |
|  |  | Purine Metabolism | 74 | 0.939 | 2 | 0.241 | 1 | 1 |
|  |  | Glycerolipid Metabolism | 25 | 0.317 | 1 | 0.276 | 1 | 1 |
|  |  | Ammonia Recycling | 32 | 0.406 | 1 | 0.34 | 1 | 1 |
|  |  | Amino Sugar Metabolism | 33 | 0.419 | 1 | 0.348 | 1 | 1 |
|  |  | Fatty Acid Elongation In Mitochondria | 35 | 0.444 | 1 | 0.365 | 1 | 1 |
|  |  | Aspartate Metabolism | 35 | 0.444 | 1 | 0.365 | 1 | 1 |
|  |  | Fatty Acid Biosynthesis | 35 | 0.444 | 1 | 0.365 | 1 | 1 |
|  |  | Nicotinate and Nicotinamide Metabolism | 37 | 0.47 | 1 | 0.382 | 1 | 1 |
|  |  | Fatty acid Metabolism | 43 | 0.546 | 1 | 0.429 | 1 | 1 |
|  |  | Steroid Biosynthesis | 48 | 0.609 | 1 | 0.466 | 1 | 1 |
|  |  | Glutamate Metabolism | 49 | 0.622 | 1 | 0.473 | 1 | 1 |
|  |  | Arginine and Proline Metabolism | 53 | 0.673 | 1 | 0.501 | 1 | 1 |
|  |  | Warburg Effect | 58 | 0.736 | 1 | 0.534 | 1 | 1 |
|  |  | Pyrimidine Metabolism | 59 | 0.749 | 1 | 0.54 | 1 | 1 |
|  |  | Bile Acid Biosynthesis | 65 | 0.825 | 1 | 0.576 | 1 | 1 |
| Tai Chi v.s. Brisk Walking | 6-month visit v.s. 12-month visit | Ammonia Recycling | 32 | 0.406 | 3 | 0.00641 | 0.629 | 0.343 |
|  |  | Amino Sugar Metabolism | 33 | 0.419 | 3 | 0.007 | 0.679 | 0.343 |
|  |  | Glucose-Alanine Cycle | 13 | 0.165 | 2 | 0.0107 | 1 | 0.351 |
|  |  | Alanine Metabolism | 17 | 0.216 | 2 | 0.0182 | 1 | 0.411 |
|  |  | Glutamate Metabolism | 49 | 0.622 | 3 | 0.021 | 1 | 0.411 |
|  |  | Transfer of Acetyl Groups into Mitochondria | 22 | 0.279 | 2 | 0.0298 | 1 | 0.412 |
|  |  | Warburg Effect | 58 | 0.736 | 3 | 0.0329 | 1 | 0.412 |
|  |  | Glycine and Serine Metabolism | 59 | 0.749 | 3 | 0.0344 | 1 | 0.412 |
|  |  | Glycolysis | 25 | 0.317 | 2 | 0.0379 | 1 | 0.412 |
|  |  | Urea Cycle | 29 | 0.368 | 2 | 0.0498 | 1 | 0.488 |
|  |  | Gluconeogenesis | 35 | 0.444 | 2 | 0.0699 | 1 | 0.623 |
|  |  | Galactose Metabolism | 38 | 0.482 | 2 | 0.0809 | 1 | 0.66 |
|  |  | Methionine Metabolism | 43 | 0.546 | 2 | 0.1 | 1 | 0.712 |
|  |  | Phenylacetate Metabolism | 9 | 0.114 | 1 | 0.109 | 1 | 0.712 |
|  |  | Lactose Degradation | 9 | 0.114 | 1 | 0.109 | 1 | 0.712 |
|  |  | Pyruvaldehyde Degradation | 10 | 0.127 | 1 | 0.12 | 1 | 0.738 |
|  |  | Beta Oxidation of Very Long Chain Fatty Acids | 17 | 0.216 | 1 | 0.197 | 1 | 1 |
|  |  | Spermidine and Spermine Biosynthesis | 18 | 0.229 | 1 | 0.207 | 1 | 1 |
|  |  | Alpha Linolenic Acid and Linoleic Acid Metabolism | 19 | 0.241 | 1 | 0.217 | 1 | 1 |
|  |  | Lactose Synthesis | 20 | 0.254 | 1 | 0.227 | 1 | 1 |
|  |  | Glutathione Metabolism | 21 | 0.267 | 1 | 0.237 | 1 | 1 |
|  |  | Betaine Metabolism | 21 | 0.267 | 1 | 0.237 | 1 | 1 |
|  |  | Purine Metabolism | 74 | 0.939 | 2 | 0.241 | 1 | 1 |
|  |  | Carnitine Synthesis | 22 | 0.279 | 1 | 0.247 | 1 | 1 |
|  |  | Cysteine Metabolism | 26 | 0.33 | 1 | 0.286 | 1 | 1 |
|  |  | Mitochondrial Beta-Oxidation of Medium Chain Saturated Fatty Acids | 27 | 0.343 | 1 | 0.295 | 1 | 1 |
|  |  | Pentose Phosphate Pathway | 29 | 0.368 | 1 | 0.313 | 1 | 1 |
|  |  | Starch and Sucrose Metabolism | 31 | 0.394 | 1 | 0.331 | 1 | 1 |
|  |  | Citric Acid Cycle | 32 | 0.406 | 1 | 0.34 | 1 | 1 |
|  |  | Fructose and Mannose Degradation | 32 | 0.406 | 1 | 0.34 | 1 | 1 |
|  |  | Aspartate Metabolism | 35 | 0.444 | 1 | 0.365 | 1 | 1 |
|  |  | Fatty Acid Biosynthesis | 35 | 0.444 | 1 | 0.365 | 1 | 1 |
|  |  | Nicotinate and Nicotinamide Metabolism | 37 | 0.47 | 1 | 0.382 | 1 | 1 |
|  |  | Porphyrin Metabolism | 40 | 0.508 | 1 | 0.406 | 1 | 1 |
|  |  | Sphingolipid Metabolism | 40 | 0.508 | 1 | 0.406 | 1 | 1 |
|  |  | Pyruvate Metabolism | 48 | 0.609 | 1 | 0.466 | 1 | 1 |
|  |  | Arginine and Proline Metabolism | 53 | 0.673 | 1 | 0.501 | 1 | 1 |
|  |  | Pyrimidine Metabolism | 59 | 0.749 | 1 | 0.54 | 1 | 1 |
|  |  | Bile Acid Biosynthesis | 65 | 0.825 | 1 | 0.576 | 1 | 1 |
| Tai Chi v.s. Brisk Walking | Baseline v.s. 12-month visit | Urea Cycle | 29 | 0.396 | 4 | 0.000428 | 0.042 | 0.042 |
|  |  | Ammonia Recycling | 32 | 0.438 | 3 | 0.00799 | 0.775 | 0.285 |
|  |  | Amino Sugar Metabolism | 33 | 0.451 | 3 | 0.00872 | 0.837 | 0.285 |
|  |  | Glucose-Alanine Cycle | 13 | 0.178 | 2 | 0.0124 | 1 | 0.305 |
|  |  | Alanine Metabolism | 17 | 0.232 | 2 | 0.021 | 1 | 0.412 |
|  |  | Glutamate Metabolism | 49 | 0.67 | 3 | 0.0258 | 1 | 0.422 |
|  |  | Warburg Effect | 58 | 0.793 | 3 | 0.0402 | 1 | 0.509 |
|  |  | Glycine and Serine Metabolism | 59 | 0.807 | 3 | 0.042 | 1 | 0.509 |
|  |  | Cysteine Metabolism | 26 | 0.355 | 2 | 0.0468 | 1 | 0.509 |
|  |  | Citric Acid Cycle | 32 | 0.438 | 2 | 0.0681 | 1 | 0.602 |
|  |  | Fructose and Mannose Degradation | 32 | 0.438 | 2 | 0.0681 | 1 | 0.602 |
|  |  | Aspartate Metabolism | 35 | 0.479 | 2 | 0.0799 | 1 | 0.602 |
|  |  | Gluconeogenesis | 35 | 0.479 | 2 | 0.0799 | 1 | 0.602 |
|  |  | Phenylacetate Metabolism | 9 | 0.123 | 1 | 0.117 | 1 | 0.791 |
|  |  | Malate-Aspartate Shuttle | 10 | 0.137 | 1 | 0.129 | 1 | 0.791 |
|  |  | Pyruvaldehyde Degradation | 10 | 0.137 | 1 | 0.129 | 1 | 0.791 |
|  |  | D-Arginine and D-Ornithine Metabolism | 11 | 0.15 | 1 | 0.141 | 1 | 0.814 |
|  |  | Taurine and Hypotaurine Metabolism | 12 | 0.164 | 1 | 0.153 | 1 | 0.83 |
|  |  | Arginine and Proline Metabolism | 53 | 0.725 | 2 | 0.161 | 1 | 0.83 |
|  |  | Spermidine and Spermine Biosynthesis | 18 | 0.246 | 1 | 0.221 | 1 | 1 |
|  |  | Alpha Linolenic Acid and Linoleic Acid Metabolism | 19 | 0.26 | 1 | 0.232 | 1 | 1 |
|  |  | Betaine Metabolism | 21 | 0.287 | 1 | 0.253 | 1 | 1 |
|  |  | Carnitine Synthesis | 22 | 0.301 | 1 | 0.264 | 1 | 1 |
|  |  | Transfer of Acetyl Groups into Mitochondria | 22 | 0.301 | 1 | 0.264 | 1 | 1 |
|  |  | Purine Metabolism | 74 | 1.01 | 2 | 0.268 | 1 | 1 |
|  |  | Glycerolipid Metabolism | 25 | 0.342 | 1 | 0.294 | 1 | 1 |
|  |  | Glycolysis | 25 | 0.342 | 1 | 0.294 | 1 | 1 |
|  |  | Oxidation of Branched Chain Fatty Acids | 26 | 0.355 | 1 | 0.304 | 1 | 1 |
|  |  | Phytanic Acid Peroxisomal Oxidation | 26 | 0.355 | 1 | 0.304 | 1 | 1 |
|  |  | Phenylalanine and Tyrosine Metabolism | 28 | 0.383 | 1 | 0.323 | 1 | 1 |
|  |  | Lysine Degradation | 30 | 0.41 | 1 | 0.342 | 1 | 1 |
|  |  | Starch and Sucrose Metabolism | 31 | 0.424 | 1 | 0.352 | 1 | 1 |
|  |  | Beta-Alanine Metabolism | 34 | 0.465 | 1 | 0.379 | 1 | 1 |
|  |  | Nicotinate and Nicotinamide Metabolism | 37 | 0.506 | 1 | 0.405 | 1 | 1 |
|  |  | Galactose Metabolism | 38 | 0.52 | 1 | 0.413 | 1 | 1 |
|  |  | Propanoate Metabolism | 42 | 0.574 | 1 | 0.446 | 1 | 1 |
|  |  | Methionine Metabolism | 43 | 0.588 | 1 | 0.454 | 1 | 1 |
|  |  | Histidine Metabolism | 43 | 0.588 | 1 | 0.454 | 1 | 1 |
|  |  | Pyruvate Metabolism | 48 | 0.656 | 1 | 0.492 | 1 | 1 |
|  |  | Pyrimidine Metabolism | 59 | 0.807 | 1 | 0.567 | 1 | 1 |
|  |  | Valine, Leucine and Isoleucine Degradation | 60 | 0.82 | 1 | 0.573 | 1 | 1 |
|  |  | Tryptophan Metabolism | 60 | 0.82 | 1 | 0.573 | 1 | 1 |
|  |  | Bile Acid Biosynthesis | 65 | 0.889 | 1 | 0.603 | 1 | 1 |
|  |  | Tyrosine Metabolism | 72 | 0.984 | 1 | 0.642 | 1 | 1 |

**Table S9 Associations between Pathway/Enrichment Analysis of Metabolomics and clinical presentations among 3 Groups**

| Rating Scales | Pathway/Annotations | Raw *P* values |
| --- | --- | --- |
| PATHWAY ANALYSIS | | |
| BBS | Citrate cycle (TCA cycle) | 0.037 |
| UPDRS | Citrate cycle (TCA cycle) | 0.002* |
| UPDRS – Part III | Citrate cycle (TCA cycle) | 0.014 |
| TUG | NA | NA |
| ENRICHMENT ANALYSIS | | |
| BBS | NA | NA |
| UPDRS | Beta Oxidation of Very Long Chain Fatty Acids | 0.033 |
|  | Citric Acid Cycle | 0.044 |
| UPDRS – Part III | Beta Oxidation of Very Long Chain Fatty Acids | 0.033 |
| TUG | NA | NA |
| BBS, Berg rating scale; NA, not available; TCA, tricarboxylic acid cycle; TUG, timed up and go; UPDRS, unified Parkinson's disease rating scale  * *P* values who remained less than 0.05 after false discovery rate (FDR) correction. | | |

**Table S10 Associations between *HIP2* mRNA level and clinical presentations in Tai Chi group**

|  | HIP2 | | With covariants | |
| --- | --- | --- | --- | --- |
|  | Wald | *P* value | Wald | *P* value |
| UPDRS | 4.79 | 0.029 | 12.25 | 0.00046 |
| UPDRS Part III | 3.27 | 0.071 | 7.89 | 0.005 |
| Timed Up and Go | 0.34 | 0.56 | 0.34 | 0.56 |
| Berg Rating Scale | 2.4 | 0.12 | 4.05 | 0.044 |
