# Supplementary material for: Mechanisms of motor symptom improvement by long-term Tai Chi training in Parkinson’s disease patients
Source: Transl Neurodegener. 2022 Feb 7;11:6. doi: 10.1186/s40035-022-00280-7 (PMC8819852; doi:10.1186/s40035-022-00280-7)
Supplement: Supplementary file 3 — Additional file 3: Fig. S1. Pathway analysis in Tai Chi group relative to the Control group from baseline to six-month visit; Fig. S2 Pathway analysis in Tai Chi group relative to the Control group from six-month visit to one-year visit; Fig. S3 Pathway analysis in Tai Chi group relative to the Control group from baseline to one-year visit; Fig. S4 Enrichment analysis in Tai Chi group relative to the Control group from baseline to six-month visit; Fig. S5 Enrichment analysis in Tai Chi group relative to the Control group from six-month visit to one-year visit; Fig. S6 Enrichment analysis in Tai Chi group relative to the Control group from baseline to one-year visit; Fig. S7 Comparison of the change of HIP2 mRNA level in the 3 groups. [file 40035_2022_280_MOESM3_ESM.docx]

**Additional File 3**


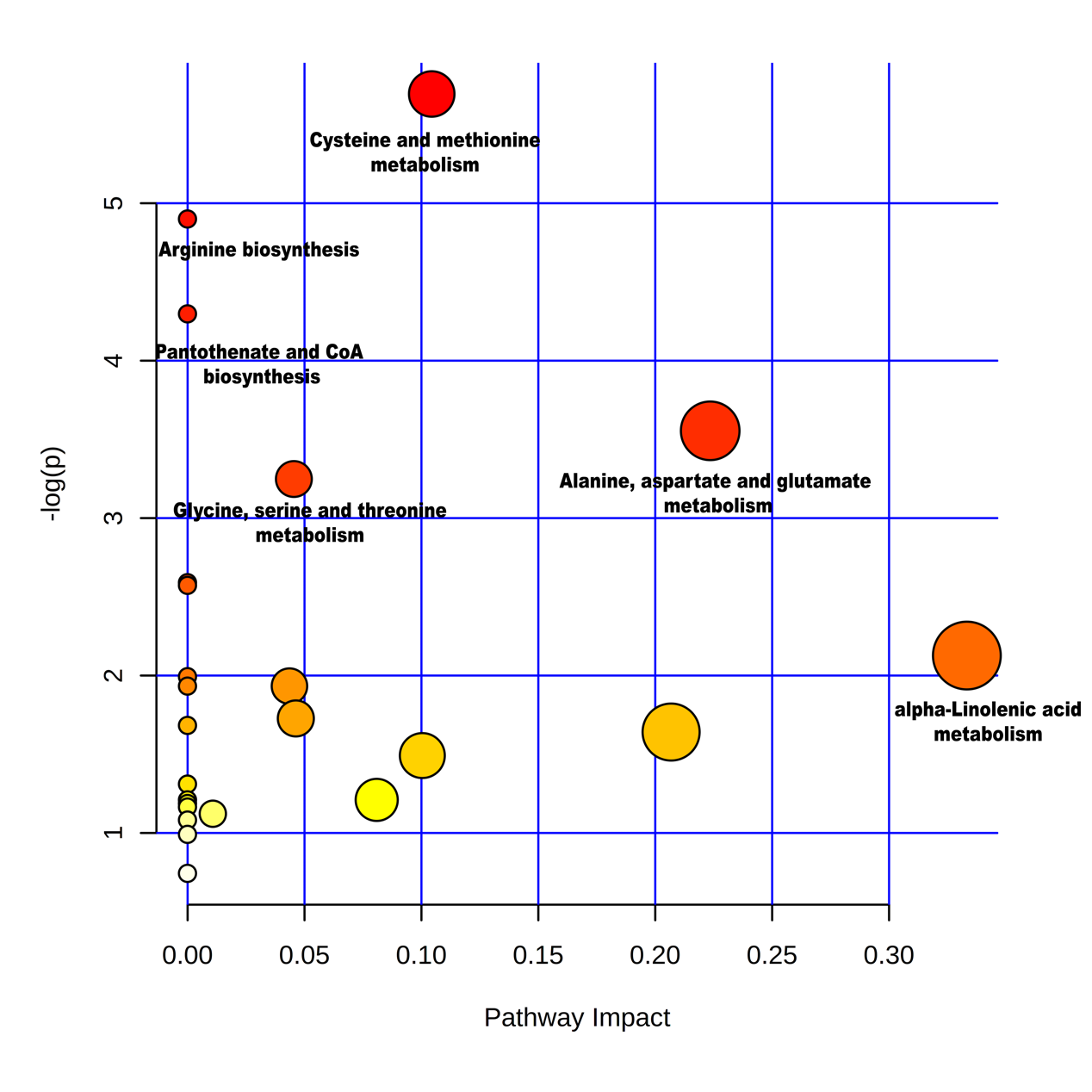


**Figure S1** Pathway analysis in Tai Chi group relative to the Control group from baseline to six-month visit


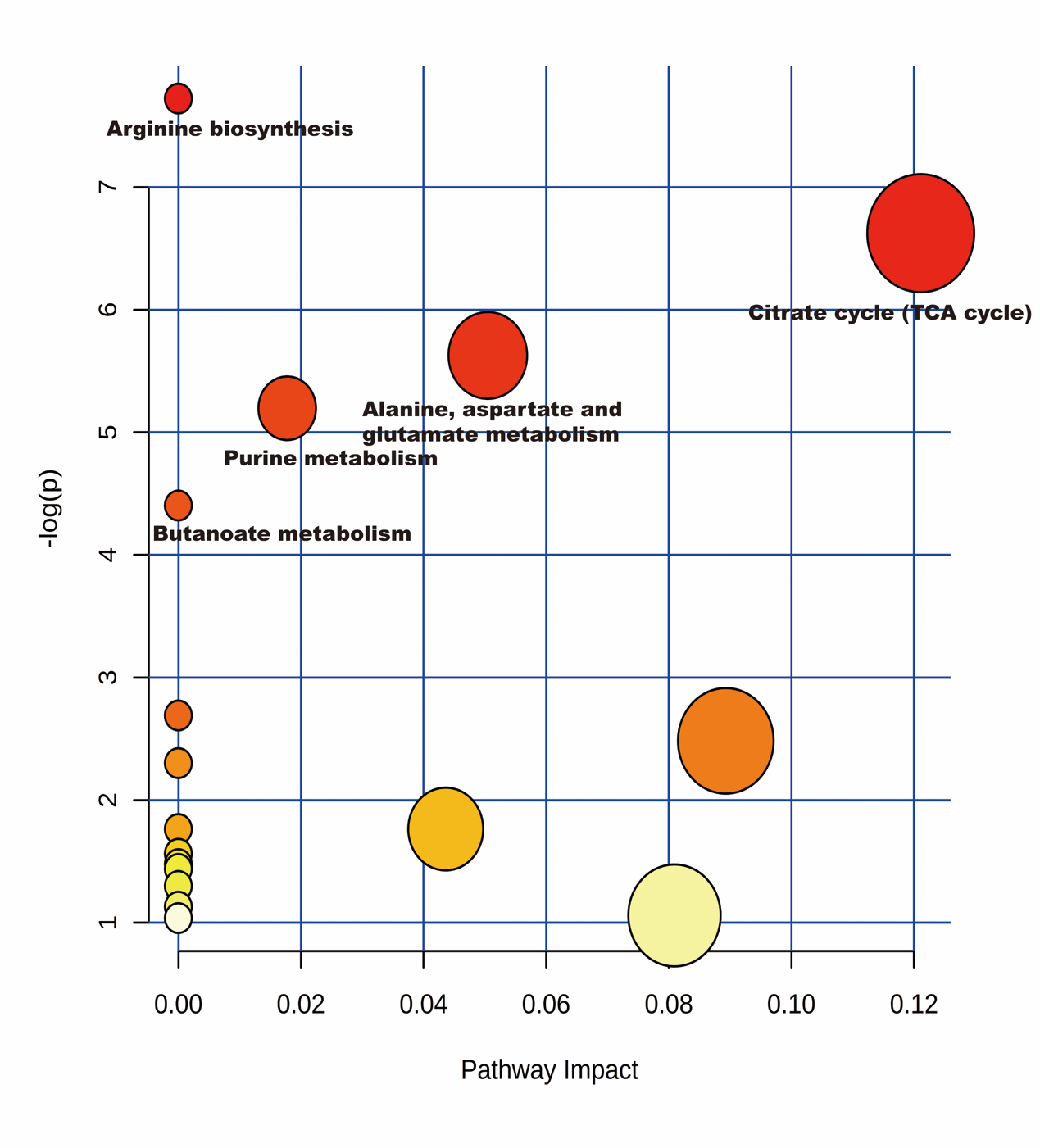


**Figure S2** Pathway analysis in Tai Chi group relative to the Control group from six-month visit to one-year visit


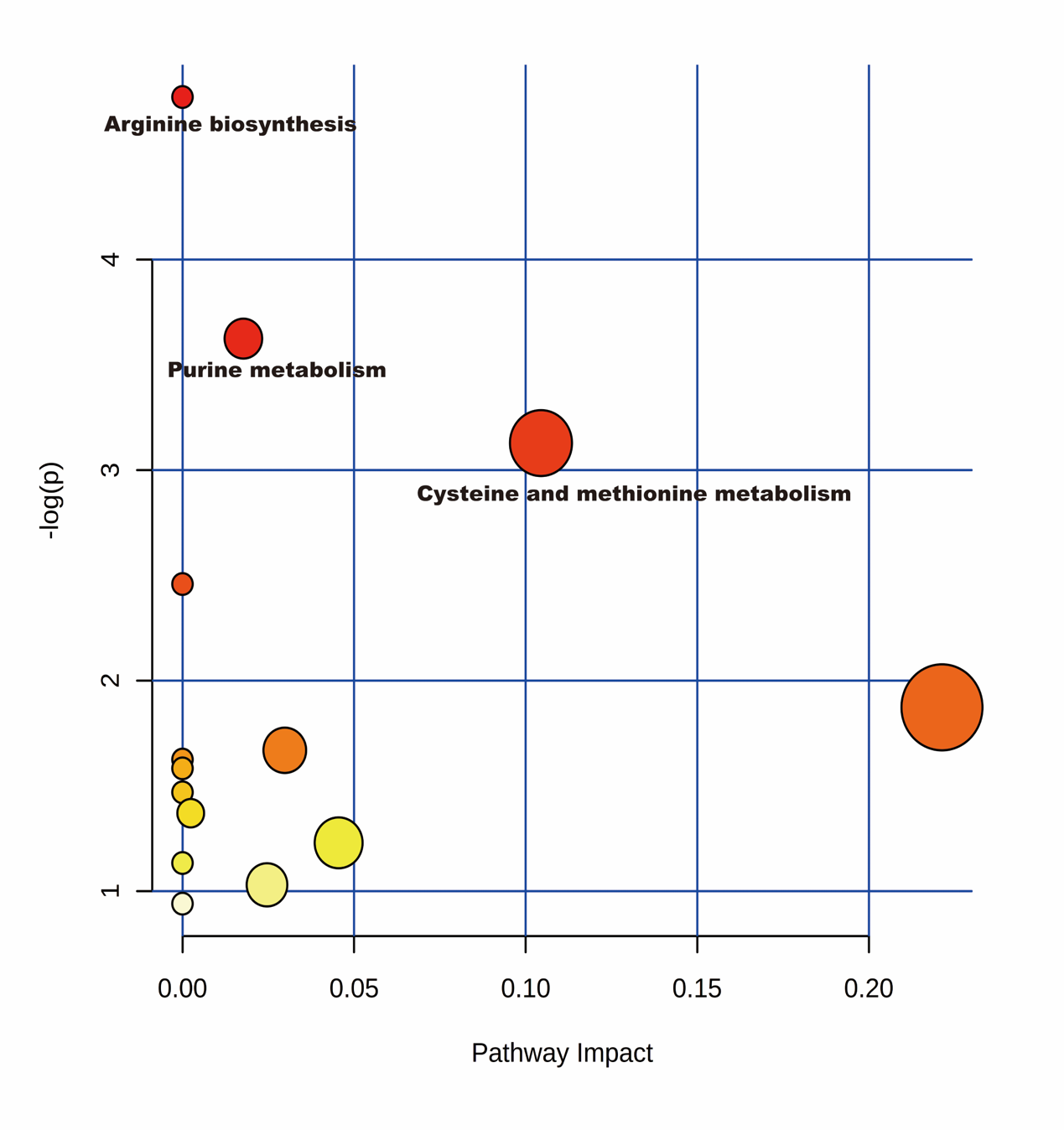


**Figure S3** Pathway analysis in the Tai Chi group relative to the Control group from baseline to one-year visit


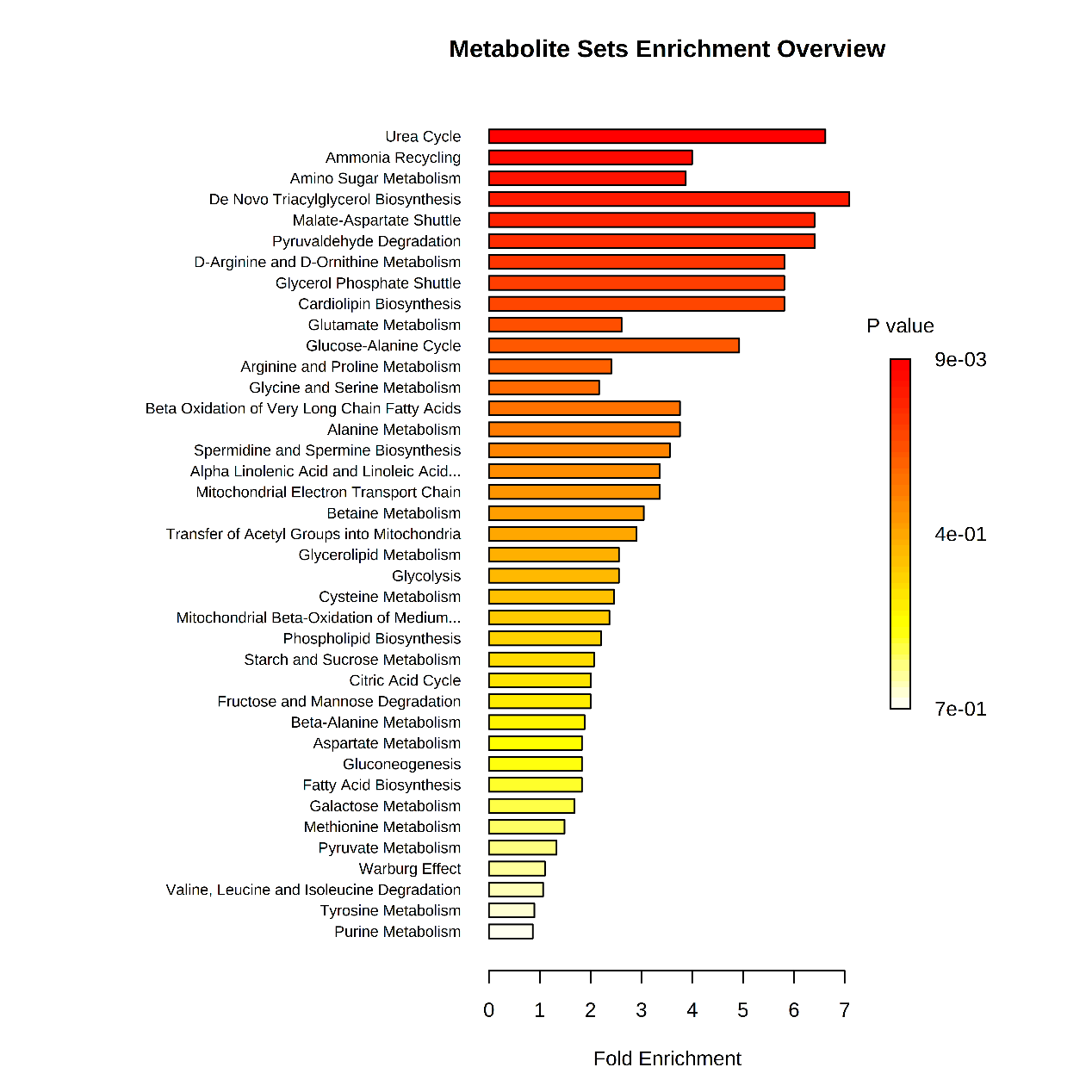


**Figure S4** Enrichment analysis in the Tai Chi group relative to the Control group from baseline to six-month visit


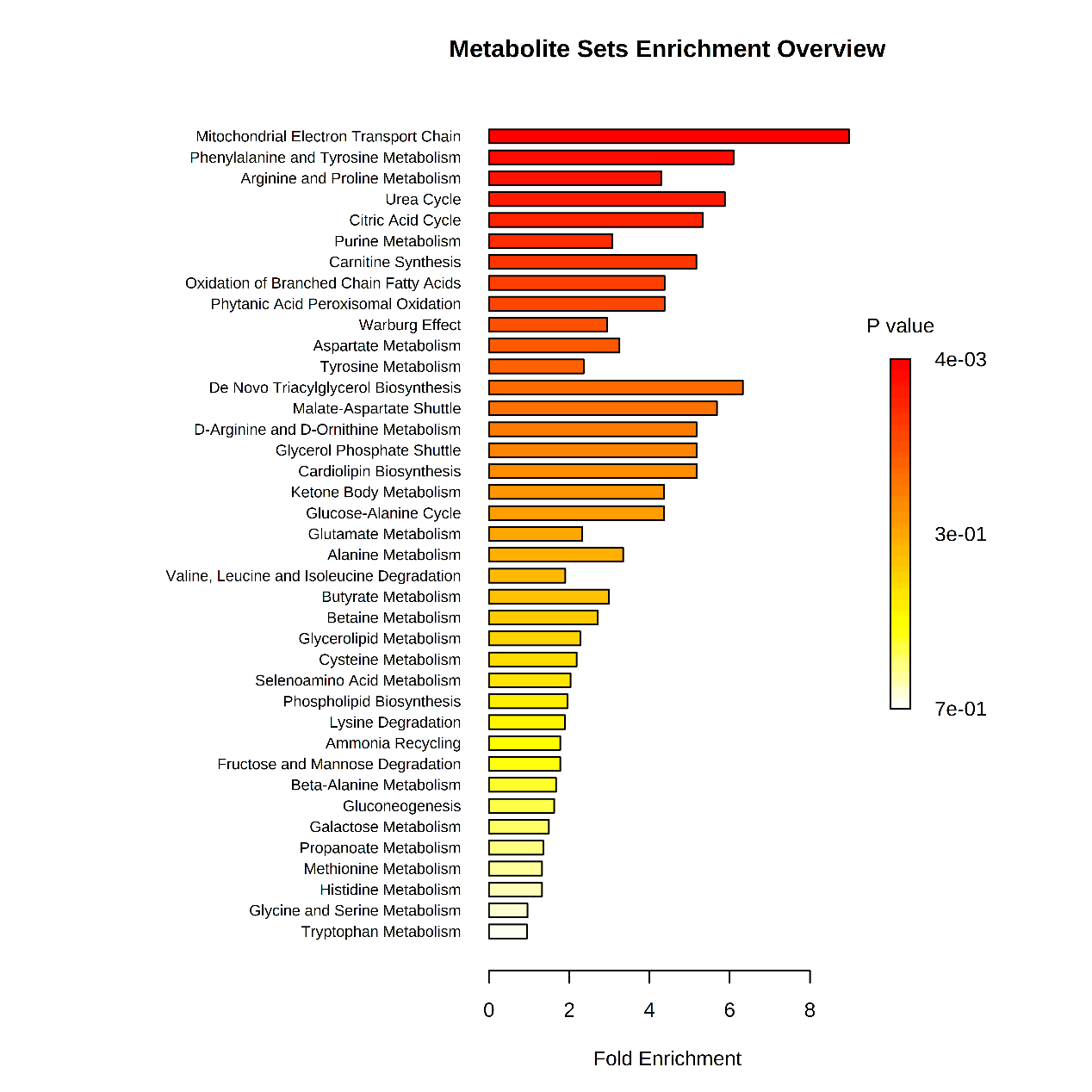


**Figure S5** Enrichment analysis in the Tai Chi group relative to the Control group from six-month visit to one-year visit


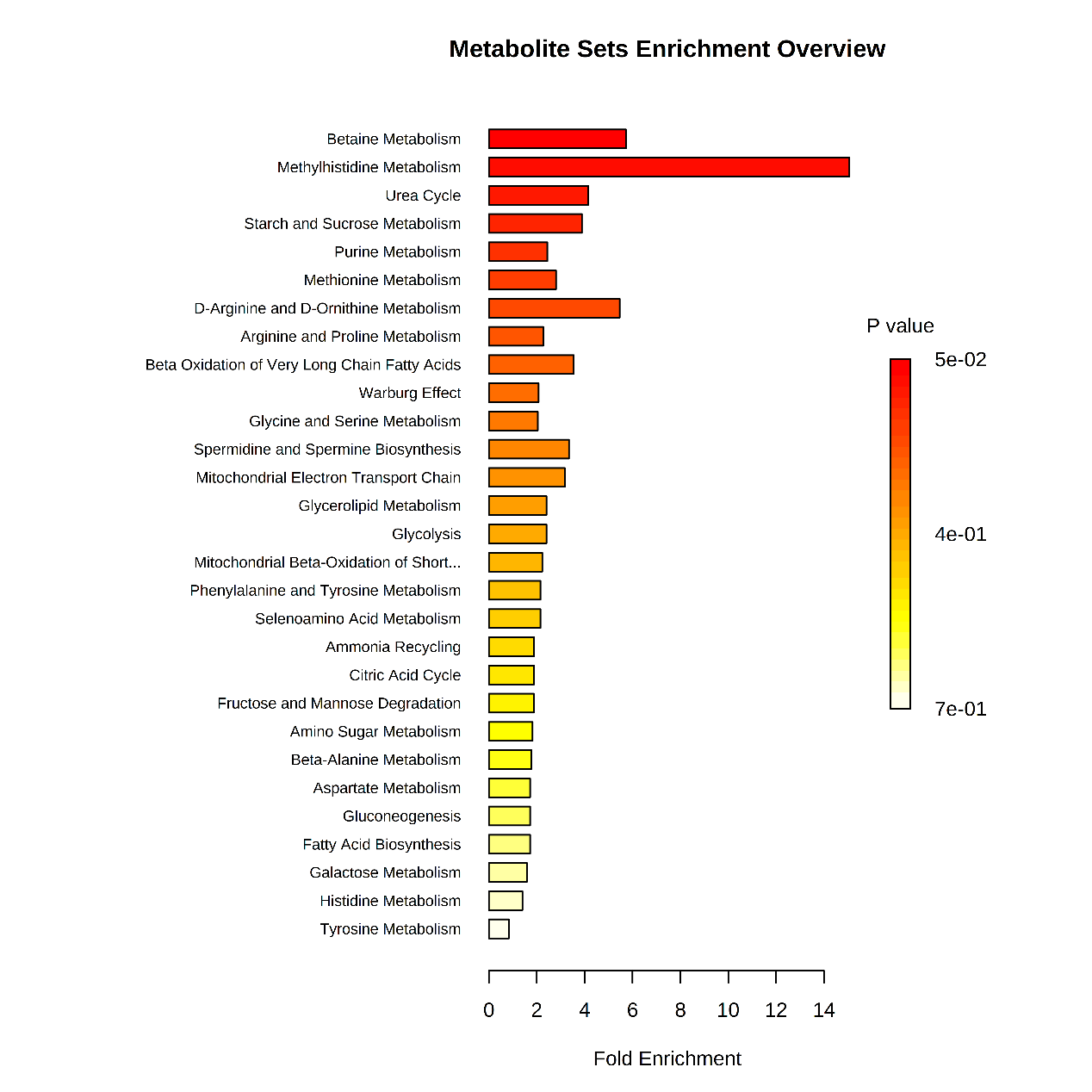


**Figure S6** Enrichment analysis in the Tai Chi group relative to the Control group from baseline to one-year visit


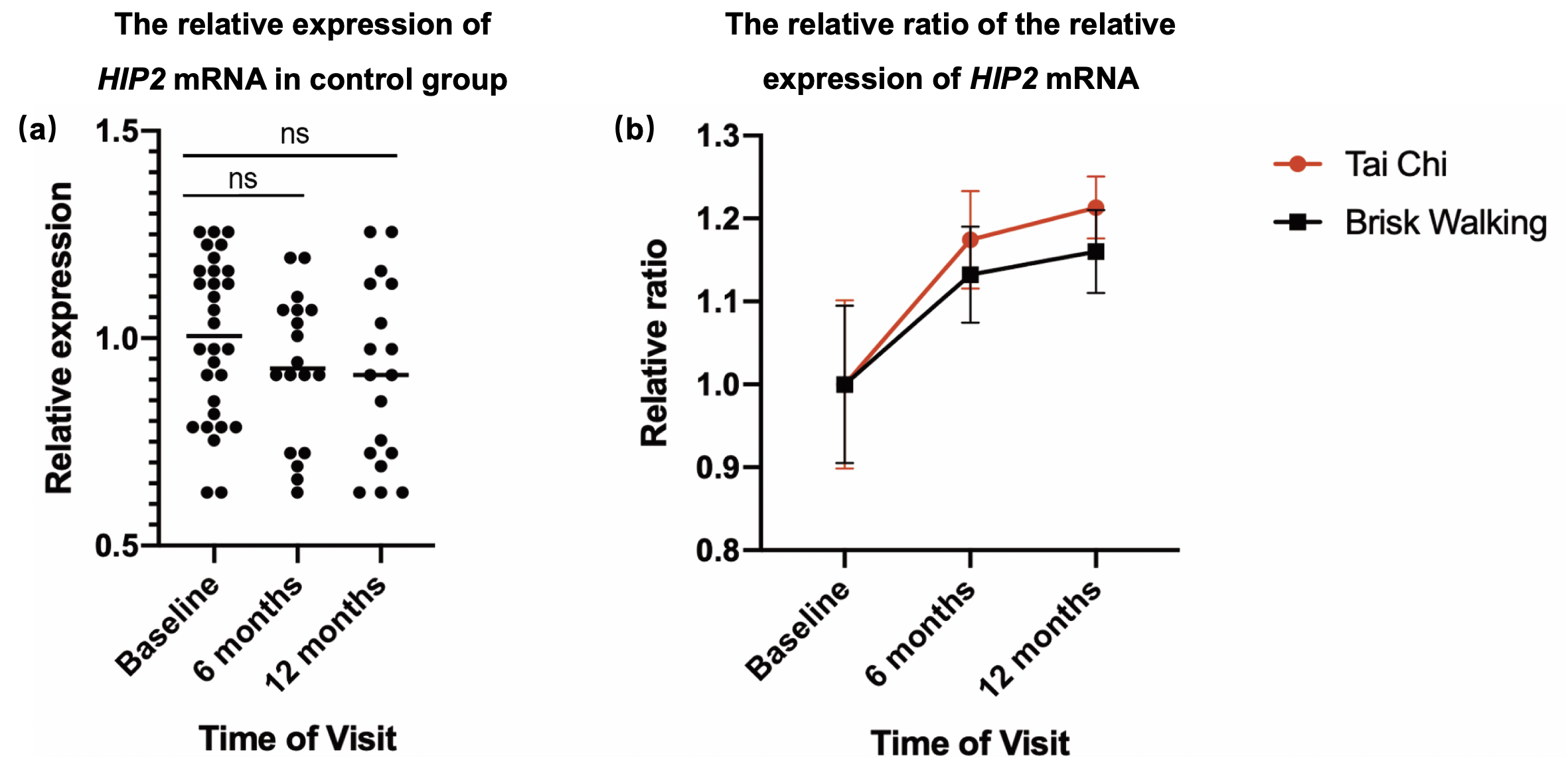


**Figure S7 Comparison between 3 groups in change of *HIP2* mRNA level.**

(**a**) There was no significant difference in the relative expression of *HIP2* mRNA among the 3 visits in the control group. The mean expression level at baseline was taken as the reference line. (b) The relative ratio of the relative expression of *HIP2* mRNA. The relative expression of *HIP2* mRNA were referenced to the mean expression of controls at the corresponding time of visit. The relative ratio was then referenced to the relative expression of baseline in each group. Data were shown as mean ± SEM.
